# Clinical health issues, reproductive hormones, and metabolic hormones associated with gut microbiome structure in African and Asian elephants

**DOI:** 10.1186/s42523-021-00146-9

**Published:** 2021-12-20

**Authors:** Mia M. Keady, Natalia Prado, Haw Chuan Lim, Janine Brown, Steve Paris, Carly R. Muletz-Wolz

**Affiliations:** 1 School for Systems Biology, George Mason University, Fairfax, VA USA; 2 Center for Conservation Genomics, Smithsonian National Zoo & Conservation Biology Institute, Washington, DC USA; 3 Center for Species Survival, Smithsonian National Zoo & Conservation Biology Institute, Front Royal, VA USA; 4 Department of Biology, Adelphi University, Garden City, NY USA

**Keywords:** Microbiome, Reproduction, Elephants, Gut–brain axis, Endocrinology, Thyroid, Metabolic, Captivity, Microbial endocrinology, Lyophilized samples

## Abstract

**Background:**

The gut microbiome is important to immune health, metabolism, and hormone regulation. Understanding host–microbiome relationships in captive animals may lead to mediating long term health issues common in captive animals. For instance, zoo managed African elephants (*Loxodonta africana*) and Asian elephants (*Elephas maximus*) experience low reproductive rates, high body condition, and gastrointestinal (GI) issues. We leveraged an extensive collection of fecal samples and health records from the Elephant Welfare Study conducted across North American zoos in 2012 to examine the link between gut microbiota and clinical health issues, reproductive hormones, and metabolic hormones in captive elephants. We quantified gut microbiomes of 69 African and 48 Asian elephants from across 50 zoos using Illumina sequencing of the 16S rRNA bacterial gene.

**Results:**

Elephant species differed in microbiome structure, with African elephants having lower bacterial richness and dissimilar bacterial composition from Asian elephants. In both species, bacterial composition was strongly influenced by zoo facility. Bacterial richness was lower in African elephants with recent GI issues, and richness was positively correlated with metabolic hormone total triiodothyronine (total T3) in Asian elephants. We found species-specific associations between gut microbiome composition and hormones: Asian elephant gut microbiome composition was linked to total T3 and free thyroxine (free T4), while fecal glucocorticoid metabolites (FGM) were linked to African elephant gut microbiome composition. We identified many relationships between bacterial relative abundances and hormone concentrations, including *Prevotella* spp., *Treponema* spp., and *Akkermansia* spp.

**Conclusions:**

We present a comprehensive assessment of relationships between the gut microbiome, host species, environment, clinical health issues, and the endocrine system in captive elephants. Our results highlight the combined significance of host species-specific regulation and environmental effects on the gut microbiome between two elephant species and across 50 zoo facilities. We provide evidence of clinical health issues, reproductive hormones, and metabolic hormones associated with the gut microbiome structure of captive elephants. Our findings establish the groundwork for future studies to investigate bacterial function or develop tools (e.g., prebiotics, probiotics, dietary manipulations) suitable for conservation and zoo management.

**Supplementary Information:**

The online version contains supplementary material available at 10.1186/s42523-021-00146-9.

## Background

The gut microbiota of captive animals is a useful system to understand host microbiota relationships [1]. Studying microbiota of captive animals offers an alternative approach to model systems (mice and humans) given the ability to re-sample individuals, manipulate aspects of their environment (e.g., diet or enrichment), and access to health and pedigree records. Manipulations of the gut microbiota may serve to improve the welfare of captive animals long-term, yet we are in the infancy of understanding host–microbiome relationships, especially in non-model systems [2–6]. In model systems, including mice and humans, the gut microbiome is an important factor in a number of health conditions including hormone regulation [6, 7], gastrointestinal disorders [8–10], and obesity [11–13]. In zoo insurance populations, staff, keepers, and veterinarians strive to provide the best care to captive animals, but unfortunately captive animals often experience health issues. For instance, African elephants (*Loxodonta africana*) and Asian elephants (*Elephas maximus*) experience gastrointestinal issues, low reproductive rates, high body condition, and lameness/stiffness in captivity [14–16]. Captive elephants provide a unique study system to examine connections between gut microbiota and clinical health concerns. We examined the gut microbiome alongside extensive clinical health and reproductive and metabolic hormone records of *L. africana* and *E. maximus* to advance our understanding of the captive elephant gut microbiome and its link to health conditions in captivity.

Monitoring the endocrine system is an essential piece to managing the health of captive animals [17]. The endocrine system is integral in metabolism, digestion, mood, reproduction, growth and development, among others physiological processes [18]. Endocrine glands and organs are located throughout the body and contribute to endocrine function. The endocrine system is primarily controlled by the hypothalamus gland located in the forebrain where it connects with the nervous system. Other endocrine glands and organs include pituitary and pineal glands (brain), thyroid and parathyroid glands (neck), thymus gland (between the lungs), adrenal glands (above the kidneys), pancreas, ovaries and testes. Hormones are secreted into the blood stream from these glands/organs and travel to organs or tissues that respond to hormonal signals. Hormone secretion is often involved in negative feedback mechanisms resulting in precise responses to physiological conditions (e.g., insulin and glucose). Yet, interference from endocrine disrupting chemicals, tumors, or gut microbiota can alter physiological functions. For instance, endocrine disrupting chemicals, such as phytoestrogen, have caused infertility in captive southern white rhinos consuming diets rich in phytoestrogens [19–21]. The ability of microbes to secrete and respond to hormones has gained greater attention, driving the newly recognized field of microbial endocrinology.

Microbial endocrinology studies the mechanism by which gut microbes communicate with the endocrine system [22, 23]. Proposed mechanisms of communication include bacteria (i) producing metabolites such as short chain fatty acids (SCFAs) that can bind to G-protein receptors on enteroendocrine-cells in the GI epithelium and influence feeding/satiation [24–28], (ii) producing neurotransmitters such as gamma-aminobutyric acid (GABA) which regulates corticosterone and psychological behavior [29], and (iii) producing compounds such as tryptophan which can be transformed into neurotransmitters like serotonin to affect mood [30–32]. These mechanisms of microbes producing metabolites, neurotransmitters, and bioactive compounds fall under the umbrella term, the gut–brain axis. The gut–brain axis is defined as the bi-directional communication between the central nervous system (CNS) and the enteric nervous system (ENS) [33, 34]. The gut–brain axis involves pathways in the CNS, ENS, endocrine system, hypothalamic pituitary adrenal (HPA) axis, and immune system [35]. Mechanisms of the brain influencing gut microbiota include altering mucus production, intestinal permeability, and immune function [33]. In model systems, the gut–brain axis has been at the forefront of microbiome research given how disruption to the gut–brain axis can affect stress, behavior, and a range of health issues [7]. Evidence reviewed in Neuman et al. [36] and Williams et al. [6] demonstrate connections between hormones and gut microbiomes in model species (mice, rats) and non-model species (rhinos, zebrafish, yellow-legged gulls, japanese flounder). Studying the gut microbiome of captive animals may reveal gut microbiota linked to longstanding health concerns.

The importance of the gut microbiome in health conditions in model species [13], humans [37], and wildlife [2, 3] suggests the gut microbiome may similarly be linked to health in elephants. Currently, 51.6% of female African elephants and 26.7% of female Asian captive elephants are acyclic or irregular [14], meaning they lack of a normal reproductive cycle. Acyclicity limits the ability of breeding programs to become self-sustaining. Acyclicity has been linked to chronically high levels of circulating prolactin (a pituitary disorder called hyperprolactinemia) with an increased probability of developing the longer the elephant is acyclic [14, 38]. Additionally, high body mass index (BMI) and high body condition scores (BCS) have been linked to acyclicity [39, 40]. Most captive elephants are overweight with 74% having a BCS of either a 4 or 5 (BCS: 1 = thinnest; 5 = fattest; ideal score = 3) [16]. Gastrointestinal issues (colic, bloat, abnormal feces) are the most common clinical event affecting 42% of the captive elephant population in North America [15]. Lameness/stiffness (reduced range of motion, favoring one or more limb, abnormal gait) affects 38% of the zoo population, though is more common in males and is associated to older age in captive elephants [15]. Lastly, metabolic hormones have been monitored in captive elephants and though not associated to acyclicity [41], may be important to elephant metabolism. Understanding and mediating health conditions in captivity is important to optimizing elephant welfare in captivity and studying the gut microbiome in these animals can provide a new tool to confront these health issues.

Here, we leveraged fecal samples and health data from an extensive collection of African and Asian elephant samples collected from 50 North American zoos to (i) quantify the role of host species and zoo facility on the elephant gut microbiome, (ii) assess the link between gut microbiomes and reproductive hormones, metabolic hormones, and clinical health variables (BCS, GI issues, lameness/stiffness, antibiotics, nonsteroidal anti-inflammatory drugs [NSAIDs], age), and (iii) identify bacterial taxa associated with reproductive and metabolic hormone concentrations.

## Methods

### Data collection

To assess the associations between the elephant gut microbiome, clinical health issues, and reproductive and metabolic hormones, we leveraged fecal and serum samples and health records from a large multi-institutional Elephant Welfare Study (EWS) conducted across North American zoos in 2012 [14–16, 42, 43]. We included a total of 117 elephants (69 African, 48 Asian) from the EWS housed across 50 North American zoos in 2012. We leveraged hormone data from serum and fecal samples, clinical data, and BCS previously collected and assessed during the EWS. We conducted a pilot study to support the use of lyophilized fecal samples for microbiome analysis (see Additional file 1).

#### Clinical data

During the EWS, 12 consecutive months of medical records were requested for each elephant to coincide with the collection of fecal and serum samples [15]. Records were examined and clinical events were defined as described by Edwards et al. [15]. We used the described records to determine if elephants in our study had a recent GI issue, lameness or stiffness, or were administered antibiotics or NSAIDs within 6 weeks preceding the fecal sample used in microbial analyses (Table 1). These ‘clinical health concerns’ were categorical variables described as present or absent 6 weeks prior to the fecal sample. Body condition scores (BCS) were used to assess body fat in captive elephants. Elephants were assigned a BCS (1 = thinnest; 5 = fattest) from standardized photographs for each elephant as described in Morfeld et al. [16]. We used BCS as a categorical variable in assessing its relationship with gut microbiome structure. The age of the elephant at the beginning of medical record collection in the 2012 EWS was included as a continuous variable in our linear mixed effects models.

**Table 1:** Overview of clinical health variables in captive African elephants and Asian elephants

Clinical health variable	Definition	African (n = 61)	Asian (n = 38)
Body Condition Score (BCS)	Scoring system to assess physical condition; 1 = thinnest; 5 = fattest [16]	*Categorical* BCS 3 n = 4 BCS 4 n = 29 BCS 5 n = 28	*Categorical* BCS < 3 n = 3 BCS 3 n = 5 BCS 4 n = 7 BCS 5 n = 23
Gastrointestinal (GI) Issues	Occurrence of colic, bloat, or abnormal feces within 6 weeks prior to fecal sample used in microbial analyses [15]	*Categorical* Y: n = 8 N: n = 53	*Categorical* Y: n = 3 N: n = 35
Lameness/stiffness	Record of reduced range of motion, favoring one or more limb, abnormal gait within 6 weeks prior to fecal sample used in microbial analyses [15]	*Categorical* Y: n = 5 N: n = 56	*Categorical* Y: n = 7 N: n = 31
Antibiotics and Non-Steroidal Anti-Inflammatory Drugs (NSAIDs)	Record of antibiotics or NSAIDs administered within 6 weeks prior the fecal sample used in microbial analyses [2]	*Categorical—Not included in analyses due to sample size* Y: n = 0 N: n = 61	*Categorical* Y: n = 3 N: n = 35
Age	The age of the elephant (in years) on date medical record review began [15]	*Continuous* Range: 26–52 Median: 33 Mean: 33.9	*Continuous* Range: 22–52 Median: 41 Mean: 40.0
Zoo	Zoo facility where elephant was housed during EWS [15]	*Categorical* n = 22	*Categorical* n = 25

This table details the clinical health variables assessed in a subset of zoos with two or more elephants

#### Serum and fecal hormone data

Blood and fecal samples were collected bi-weekly in 2012 for the EWS. Blood samples were collected without anesthesia in the morning hours from either an ear or leg vein. Blood was allowed to clot at room temperature, centrifuged (~ 1500 g) and the serum stored frozen at − 20 °C or colder [14]. Serum samples were shipped to the Endocrinology Research Laboratory at the Smithsonian Conservation Biology Institute Center for Species Survival (SCBI-CSS) on dry ice and permanently stored at − 20 °C until analyzed. Fecal samples were collected by keepers from the ground in the morning within 2 h of defecation, mixed to obtain homogeneity, 5–10 subaliquots (~ 50–100 g) placed into Whirl–Pak^®^ plastic bags, and frozen (− 20 °C) immediately [42]. Fecal samples were sent to SCBI-CSS where they were lyophilized (Labconco, Kansas City, MO) and stored frozen at − 20C until analyzed [42].

Serum and fecal samples were used to quantify concentrations of reproductive and metabolic hormones (see Table 2 for hormone descriptions). Reproductive hormones quantified from serum samples were: progestagens, prolactin (PRL), luteinizing hormone (LH), and follicle stimulating hormone (FSH) [14]. Progestagens are secreted from the ovaries and increase during ovulation, while PRL, LH and FSH are secreted from the anterior pituitary gland and regulate parts of the ovarian cycle. Metabolic hormones quantified from serum samples were: thyroid stimulating hormone (TSH), free thyroxine (Free T4), total thyroxine (Total T4), and total tri-iodothyronine (Total T3) [44]. Briefly, TSH is produced in the anterior pituitary, stimulates the thyroid gland to secrete pro-hormone T4, which is transformed into T3 in target organs. T4 and T3 circulate in plasma as either bound to transport protein or unbound. We included two measures of T4: (i). ‘Free T4’ being unbound, and (ii.) ‘Total T4’ being bound and unbound hormone. Free T4 can move into target organs and be transformed into T3. We used the yearly average of serum hormones (progestagens, prolactin, LH, FSH, and TSH, Free T4, Total T4, Total T3) for each elephant included in our study to test associations to gut microbiome structure. The yearly average for each serum hormone was selected because many hormones were assessed once a month in the EWS and could not be consistently matched to the date of the fecal sample used in microbiome analysis. We confirmed daily serum hormone values were significantly correlated to yearly averages in a subset of our data using a paired correlation test (Additional file 2: Table S1). This supported the use of yearly serum hormone averages available for all elephants in our dataset. Additionally, fecal samples were used to quantify fecal glucocorticoid metabolites (FGM) [42]. FGMs are involved in metabolism and often used as a measure of physiological stress [45–48]. FGM concentrations did correspond to the day of the microbiome sample and were included in our models as day values.

**Table 2:** Overview of reproductive and metabolic hormones in captive African elephants and Asian elephants

Reproductive hormones	Function	African (n = 61)	Asian (n = 38)
Progestagens (ng/ml)	Produced by the corpora lutea of ovary, involved in pregnancy and menstrual cycle, concentrations increase after ovulation	*Continuous* Range: 0.052–0.607 Median: 0.17 Mean: 0.21	*Continuous* Range: 0.052–0.562 Median: 0.25 Mean: 0.25
Prolactin PRL (ng/ml)	Produced by the anterior pituitary, promotes lactation, involved in normal follicular function, maintains homeostasis [118, 119]	*Continuous* Range: 2.44–105.24 Median: 11.57 Mean: 17.40	*Continuous* Range: 2.34–21.26 Median: 5.25 Mean: 6.30
Luteinizing hormone LH (ng/ml)	Produced by the anterior pituitary, initiates ovulation, develops and maintains corpus luteum	*Continuous* Range: 0.59–2.64 Median: 0.99 Mean: 1.08	*Continuous* Range: 0.47–3.45 Median: 1.24 Mean: 1.36
Follicle-stimulating hormone FSH (ng/ml)	Produced by the anterior pituitary, promotes follicular development	*Continuous* Range: 1.07–5.53 Median: 2.66 Mean: 2.73	*Continuous* Range: 2.84–7.38 Median: 4.11 Mean: 4.42

Metabolic hormones	Function	African (n = 61)	Asian (n = 38)
Fecal glucocorticoid metabolites FGM (ng/g)	Produced by the adrenal cortex; functions in metabolizing carbohydrates, proteins, and fats [45, 46], induces glucose synthesis [47]. Often used as a measure of chronic physiological stress in wildlife [48]	*Continuous* Range: 21.02–249.53 Median: 84.22 Mean: 93.68	*Continuous* Range: 20.92–328.29 Median: 108.20 Mean: 128.13
Thyroid stimulating hormone TSH (ng/ml)	Produced by the anterior pituitary, TSH stimulates the thyroid gland to produce T4, which can be transformed into T3 [120]	*Continuous* Range: 0.19–1.73 Median: 0.85 Mean: 0.88	*Continuous* Range: 0.12–1.88 Median: 1.04 Mean: 1.07
Total triiodothyronine Total T3 (ng/dl)		*Continuous* Range: 38.04–117.64 Median: 85.7 Mean: 85.52	*Continuous* Range: 52.06–165.89 Median: 95.97 Mean: 98.38
Total thyroxine Total T4 (µg/dl)	Produced by the thyroid gland, T4 and T3 control metabolism, homeostasis, and growth [121, 122]. T4 and T3 circulate in plasma bound to transport protein and unbound. Unbound hormone is referred to as “free”, whereas “total” measures bound and unbound hormone. Free T4 moves to target organs where it is transformed to T3	*Continuous* Range: 6.77–14.72 Median: 9.46 Mean: 9.75	*Continuous* Range: 6.48–12.64 Median: 10.02 Mean: 10.20
Free thyroxine Free T4 (ng/dl)		*Continuous* Range: 0.49–1.17 Median: 0.84 Mean: 0.83	*Continuous* Range: 0.44–1.23 Median: 0.71 Mean: 0.75

Description of general function and range of reproductive and metabolic hormones in African and Asian elephants (dataset includes zoos with two or more elephants)

#### Effect of lyophilization on gut microbiome communities

In order to use lyophilized fecal samples from the EWS study, we conducted a pilot study at the Smithsonian National Zoo to assess the effects of lyophilization and time until freezing on gut microbiome samples compared to fresh fecal samples (see Additional file 1: Figs. S1–S3). We assessed differences in the gut microbiome among seven Asian elephants in which fecal samples were collected (i) fresh, (ii) fresh then lyophilized, (iii) 6 h after defecation then lyophilized, and (iv) 10 h after defecation then lyophilized. We concluded that though abundance of certain bacterial phyla showed some evidence of variation between fresh and lyophilized samples, individual elephants can be identified through their gut microbiome profiles even when using lyophilized samples (see Additional file 1). This ultimately provided support for us to use the EWS samples. This conclusion is further supported by Blekhman et al., who also found individual signature remained in lyophilized fecal samples [49].

#### Microbiome sample selection

We examined gut microbiomes of 69 African elephants and 48 Asian elephants from a total of 50 facilities. We selected elephants and samples from the EWS based on the following criteria: (i) only female elephants were chosen, (ii) elephants deceased or transferred within the study window were excluded, (iii) individuals without BCS or clinical health data were excluded, (iv) elephants were included if decidedly cycling or acyclic (i.e., no pregnant, lactating, or irregular cycling elephants were included), (v) only adult elephants above the age of 22 (aged 22–52) were included, and (vi) all fecal samples were chosen within 6 weeks of each other from June 17, 2012–July 28, 2012.

### Molecular methods and sequencing

We extracted DNA from 0.05 g of lyophilized fecal samples using QIAamp PowerFecal DNA Kit (Qiagen, UK) following manufacture’s protocol. The guidelines for using 0.25 g of sample was reduced to 0.05 g due to sample lyophilization [49]. A negative control was included in each set of extractions (batch size: 18–36 samples extracted at once). We included a Zymobiomics Microbial community standard extraction control (Zymo, USA; Catalog No. D6300).

The 16S rRNA gene was targeted with primers, amplified and sequenced to identify bacteria in fecal samples. We used forward primer 515F-Y and reverse primer 939R to amplify the V3-V5 region of the 16S rRNA gene (Additional file 2: Table S2) [50, 51]. We performed polymerase chain reactions (PCR) in 25-ul reactions using 12.5 μl of 2X KAPA HiFiHotStart ReadyMix (Kapa Biosystems, USA), forward and reverse primers at 0.3 uM concentration, 1 μl of BSA at 20 mg/ml, and 2 μl of DNA. We amplified each sample in duplicate and included a negative control with each set of PCR reactions. PCR conditions were: 95 °C for 3 min, followed 25 cycles of 98 °C for 20 s, 62 °C for 15 s, 72 °C for 15 s, and a final extension (72 °C for 1 min). Specific i5 and i7 primers were added to each sample to conduct dual-indexing and to identify individual elephants post sequencing. We ran 10 cycles of index PCR using 25 μl reactions with, 12.5 μl of Kapa HiFi HotStart ReadyMix (Kapa Biosystems, USA), 3 μl of i5 and i7 primers, and 2 μl of amplicon PCR products. PCR conditions were: 95 °C for 3 min, 98 °C for 20 s, 62 °C for 15 s, and 72 °C for 15 s, and a final extension (72 °C for 1 min). After each PCR reaction, we verified DNA amplification and target amplicon size with gel electrophoresis and performed post-PCR clean-ups using Speed-beads (in a PEG/NaCl buffer) [52]. We quantified DNA concentration using Qubit 4 Fluorometric Quantification (InvitrogenThermoFisher Scientific, USA), and pooled samples in equimolar amounts. The desired amplicon size was selected using E-gel Size Select 2 (Invitrogen ThermoFisher, USA) and we confirmed the average amplicon size of 578 base pairs using Tape Station 4200 (Agilent, USA). We performed two MiSeq runs (2 × 300 bp) on an Illumina MiSeq at the Center for Conservation Genomics, Smithsonian National Zoo & Conservation Biology Institute.

### Sequence data processing

All data analysis was conducted in RStudio (v 1.1.463) for R (v3.5.2). Two sequencing runs were combined to achieve optimal sequencing depth following dada2 (v1.13.1) workflow for Big Data. We quality filtered data and identified unique bacterial taxa referred to as amplicon sequence variants (ASVs) using the dada2 package [53, 54]. ASVs were assigned taxonomy using Ribosomal Database Project classifier (RDP) [55]. A phylogenetic tree was built using FastTree [56] in QIIME2 [57]. We exported ASVs, the taxonomy table, and the meta data to be used in the phyloseq package for further analysis [58]. Decontam package (v1.1.2) was used to filter 35 potential contaminants using the combined method which assessed frequency and prevalence of potential contaminants [59]. We filtered to remove singletons, ASVs not classified as Bacteria, and ASVs classified as Cyanobacteria. The Zymobiomics Microbial community extraction standard was analyzed and we found genera in similar relative abundances as described by Zymo (Catalog No. D6300).

### Data analysis

#### Data overview

We had the following specific questions—(i) how does the gut microbiome differ by species and zoo facility, (ii) is the gut microbiome associated with clinical health concerns (BCS, GI issues, lameness/stiffness, antibiotics and NSAIDs, age), reproductive hormones, and metabolic hormones, and (iii) are there specific bacteria taxa associated with reproductive and metabolic hormone concentrations?

#### Microbiome composition by species and zoo facility

We first characterized the overall taxonomic patterns of the gut microbiome of African (n = 69) and Asian (n = 48) elephants. To present overall taxonomic patterns, we quantified the relative abundance of bacterial phyla in both African and Asian elephants. We assessed the core microbiome by identifying ASVs consistently present in the gut microbiome of African and Asian elephants (in > 80% of samples).

We tested if alpha and beta diversity differed between species and among zoos. For alpha diversity, we chose to use bacterial ASV richness and Phylogenetic Diversity (PD) as metrics for alpha diversity (diversity within a single community). Bacterial ASV richness is the number of bacterial taxa in the community and Faith’s PD represents the total tree branch length found in a sample [60]. To calculate Faith’s PD, we measured tree branch length with R package picante using the function *pd* to assign each elephant a phylogenetic diversity score [61]. We used ANOVA to test bacterial ASV richness and phylogenetic diversity as the response variable and species and zoo as the explanatory variables. For beta diversity, we used Bray–Curtis (abundance-weighted), Jaccard (presence–absence), and unweighted UniFrac (presence–absence phylogenetic distance) to measure differences in bacterial community composition between species and among zoos. Prior to conducting Bray–Curtis analyses, we performed proportion normalization on the raw sequence counts to correct for biases associated with unequal sequencing depth on this abundance-weighted metric [62, 63]. Variation in sequence coverage was minimal (5x difference in sequencing depth), so we did not normalize sequence coverage. All other alpha and beta diversity metrics should be minimally impacted by differences in sequencing coverage (Additional file 2: Fig. S4) [62, 63]. We used PERMANOVA (*adonis* function*;* vegan package) to test the beta diversity measures as the response and species and zoo as the explanatory variables. We performed PERMDISP (*betadisper* function; vegan package) to verify that variation in dispersion was not driving the significance [64]. When assessing dispersion among zoo facilities, we found zoos with only one individual or zoos co-housing both elephant species drove dispersion, and performed subsequent analyses eliminating these facilities to confirm significant bacterial compositional differences between zoos.

We tested the association between geographic distance among zoos and microbial dissimilarity in African and Asian elephants. We used the Mantel test (*mantel* function, vegan package) to test if gut microbiome communities were more similar among zoos near one another [64]. We created a distance matrix for zoos based on latitude and longitude (*geodist* fuction, geodist package) [65]. We merged bacterial ASVs from elephants at the same zoo and created distance matrices using Bray–Curtis, Jaccard and unweighted UniFrac measures (*distance* function, phyloseq package) [58]. We used 10,000 permutations and the spearman rank method due to non-normal distributions.

Finally, we subset to only zoos that co-housed both species to quantify the effect of species, zoo, and their interaction on the gut microbiome. We had five zoo facilities co-housing African and Asian elephants together (total sample size: African n = 6; Asian n = 9). We assessed differences in alpha diversity (species richness and phylogenetic diversity) using ANOVA with the alpha diversity metric as the response and species, zoo, and their interaction as explanatory variables. We assessed differences in beta diversity (Bray–Curtis, Jaccard, UniFrac) using PERMANOVA with the beta diversity measure as the response and species, zoo, and their interaction as explanatory variables. We extracted R^2^ values for explanatory variables from the PERMANOVA model results.

#### Relationship between gut microbiome, clinical health concerns, reproductive hormones, and metabolic hormones

We determined if measures of gut microbiome structure (alpha and beta diversity) were linked to clinical health issues, reproductive hormones, and metabolic hormones in African and Asian elephants (reviewed in Tables 1 and 2). We analyzed African and Asian elephants separately due to observed differences in bacterial community differences, physiological differences, and species-specific health concerns such as reproductive cycling [14]. Additionally, we used zoo as a random effect to account for the strong influence zoo has on the microbiome community (see “Results” section). This required dropping samples from zoos with only one elephant in our dataset, resulting in *n* = *61* African and *n* = *38* Asian elephants. We used clinical health concerns (BCS, GI issues, lameness/stiffness, antibiotics and NSAIDs, age), reproductive hormones (progestagens, PRL, LH, FSH), and metabolic hormones (TSH, total T4, free T4, total T3, FGM) as explanatory variables and alpha and beta diversity measures as response variables in our analyses (Tables 1, 2). In Asian elephants, we combined BCS of 1 and 2 together and combined recent administration of antibiotics or NSAIDs together due to low sample sizes (Table 1).

We used linear mixed effect models (*lmer* function; lmerTest package) with automatic backward selection (*step* function) for African and Asian elephants to test alpha diversity measures (bacterial ASV richness and phylogenetic diversity) as the response variable and clinical health variables (Table 1), reproductive hormones (Table 2), and metabolic hormones (Table 2) as explanatory variables with zoo facility as a random effect [66]. We report results from final models, which vary in inclusion of fixed and random effects based on significance of fixed and random effects (Additional file 2: Tables S5 and S6). Any significant explanatory variables were visualized by plotting raw values against response variables as a linear regression or boxplot. We assessed differences in bacterial community composition using beta diversity measures (Bray–Curtis, Jaccard’s, UniFrac) as response variables using PERMANOVAs. Clinical health variables, reproductive hormones, and metabolic hormones were explanatory variables, and we accounted for zoo facility effect by constraining permutations (*adonis2* function; vegan package) [64]. If PERMANOVA was significant, we performed PERMDISP (*betadisper* function; vegan package) to verify dispersion was not driving the significance [64]. In cases of highly unequal sample size, we randomly subsampled to balance the sample size. If we found that sample size drove dispersion differences and affected our statistical results of the PERMANOVA, we did not report its significance. We additionally fit vectors of significant variables onto a Multidimensional scaling (MDS) ordination to visualize shifts in the gut microbiome. To do this we used function *capscale* in the vegan package, specified an unconstrained model (null model), and fit significant explanatory variables onto the ordination (*envfit* function, vegan package) [64].

#### Relationship between specific gut bacteria, clinical health concerns, reproductive hormones, and metabolic hormones

In African and Asian elephants, we tested for relationships between relative abundance of bacterial ASVs, clinical health concerns, and hormone concentrations using two methods. First, we used a linear mixed effects model to identify bacterial ASVs associated to clinical health variables and hormone concentrations when accounting for zoo facility as a random effect (*lmer* function; lme4 package) [67]. We log-transformed bacterial relative abundances to improve normality. Second, we used TITAN (Threshold Indicator Taxa ANalysis) to identify bacterial taxa with non-linear relationships with hormone concentrations [68]. We chose this analysis because it also allowed us to identify hormone concentrations where bacterial taxa relative abundance sharply increased or decreased. We used the suggested *purity* and *reliability* cutoff of > 0.95 to identify bacterial taxa [68]. Z-scores represent the magnitude of change the bacterial taxa have in response to the hormone gradient. In both analyses we removed ASVs with a sum across samples of less than 5% abundance and ASVs present in less than five samples. This resulted in 257 ASVs across 61 African elephants and 161 ASVs across 38 Asian elephants.

## Results

### Taxonomic patterns and core microbiome

**Fig. 1:**
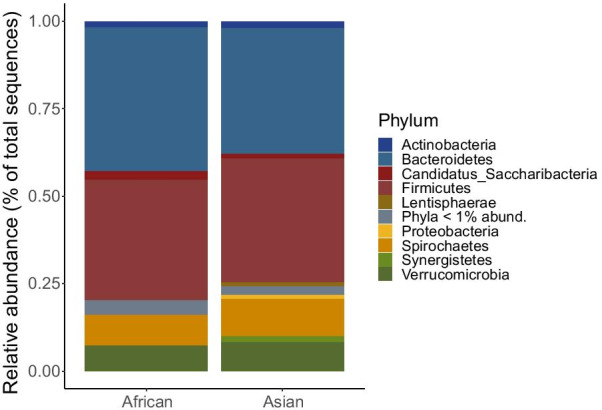
Stacked bar plot depicting relative abundance of dominant bacteria phyla in African (n = 69) and Asian (n = 48) elephants. Phyla with an average relative abundance < 1% grouped together

After filtering, we had a total of 2,875,591 high quality bacterial sequences from 117 elephant samples (69 = African, 48 = Asian). There was an average of 24,478 sequences per sample (range of 13,966–67,234 sequences). We detected a total of 6781 ASVs from 15 bacterial phyla. There were four dominant phyla across both African and Asian elephants (Fig. 1; African: Bacteroidetes: 41% mean relative abundance, n = 1136 ASVs; Firmicutes: 35%, n = 2816 ASVs; Spirochaetes: 9% n = 316 ASVs; Verrucomicrobia: 7%, n = 403 ASVs; Asian: Bacteroidetes: 36%, n = 811 ASVs; Firmicutes 36%, n = 2169; Spirochaetes: 10.1%, n = 220 ASVs, Verrucomicrobia: 8%, n = 347 ASVs). We found seven ASVs that made up the core African gut microbiome and 32 ASVs that made up the core Asian gut microbiome (present in > 80% samples; Additional file 2: Tables S3 and S4). Most bacterial ASVs in the core microbiome were unique to each elephant species, expect for three core ASVs were shared by both elephant species: *Treponema* sp. (ASV1), *Faecalitalea* sp. (ASV2), and *Bulliedia* sp. (ASV 64).

### Host species and zoo facility influence microbiome composition

**Fig. 2:**
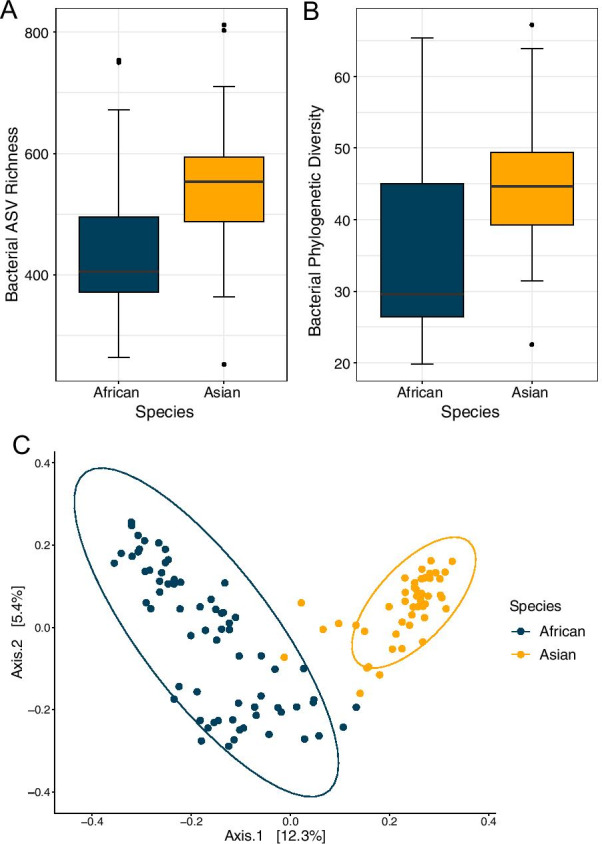
Alpha and beta diversity differ between African (n = 69) and Asian (n = 48) elephant host species. **A** Box plot of bacterial ASV richness and **B** bacterial phylogenetic diversity between host species. Asian elephants had greater bacterial ASV richness and phylogenetic diversity than African elephants (ANOVAs *p* < 0.001). **C** PCoA of Bray–Curtis distances (95% confidence ellipses shown per species) showing that African and Asian elephants have distinct gut bacterial communities in compositions (PERMANOVA *p* = 0.001) and dispersion (PERMDISP *p* < 0.001)

**Fig. 3:**
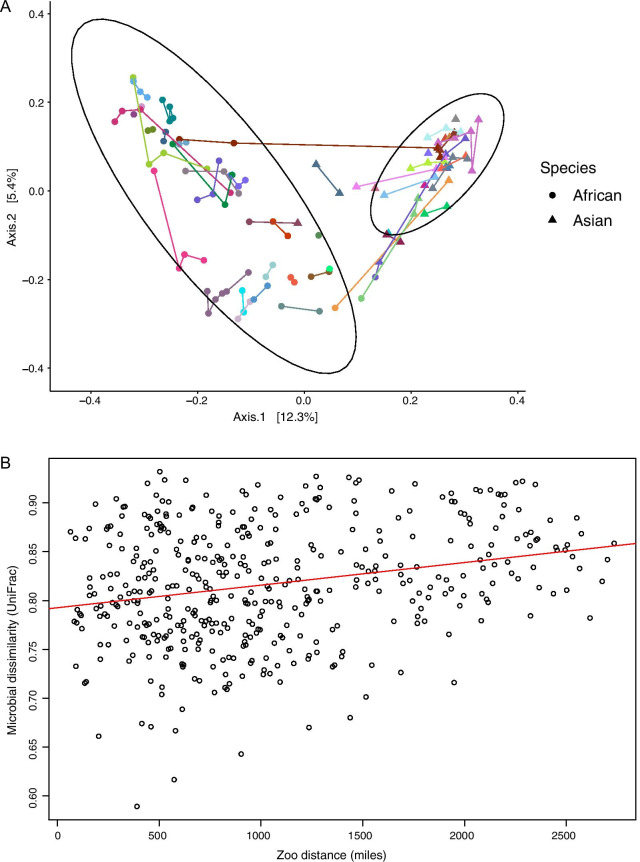
Elephant gut composition influenced by zoo facility. **A** PCoA of Bray–Curtis distances (95% confidence ellipses shown for species) showing African and Asian elephant gut microbiome compositions with zoo facilities represented as unique colors with line linkages, and host species represented by shape (circle = African elephant, triangle = Asian elephant) (PERMANOVA *p* = 0.001; zoo explained 65.15% variance). **B** African elephant gut microbiomes are more similar among zoos near one another (Mantel test unweighted UniFrac *p* = 0.0157, r = 0.218)

We found strong relationships between elephant species and zoo facilities with the gut microbiome. Asian elephants had greater bacterial ASV richness and phylogenetic diversity than African elephants (Fig. 2A; square root transformed bacterial ASV richness ANOVA *F*_1,66_ = 48.49, *p* < 0.001; phylogenetic diversity ANOVA *F*_1,66_ = 35.51, *p* < 0.001). Elephants from different zoos also varied in bacterial ASV richness and phylogenetic diversity (square root transformed bacterial ASV richness ANOVA *F*_49,66_ = 2.58, *p* < 0.001; phylogenetic diversity ANOVA *F*_49,66_ = 2.81, *p* < 0.001). We found distinct gut microbiome compositions between host species in both bacterial community composition (Fig. 2B; PERMANOVA Bray–Curtis Pseudo F_1,66_ = 27.36, R^2^ = 10.21%, *p* = 0.001; Jaccard’s Pseudo F_1,66_ = 16.16, R^2^ = 7.77%, *p* = 0.001; UniFrac Pseudo F_1,66_ = 21.42, R^2^ = 9.52%, *p* = 0.001), and bacterial community dispersion (PERMDISP Bray–Curtis F_1, 115_ = 70.99, *p* < 0.001; Jaccard’s F_1, 115_ = 80.89 *p* < 0.001; UniFrac F_1, 115_ = 60.29, *p* < 0.001). We also found distinct microbiome compositions by zoo facility (Fig. 3A; PERMANOVA Bray–Curtis Pseudo F_49,66_ = 3.56, R^2^ = 65.15%, *p* = 0.001; Jaccard’s Pseudo F_49,66_ = 2.57, R^2^ = 60.50%, *p* = 0.001; UniFrac Pseudo F_49,66_ = 2.81, R^2^ = 61.15%, *p* = 0.001). Zoo facility greatly influenced the gut microbiome and explained 65.15%, 60.50%, 61.15% (Bray–Curtis, Jaccard, and UniFrac respectively) of the variance in gut microbial community composition compared to 10.21%, 7.77%, and 9.52% explained by host species. In African elephants we found the closer zoos were to each other, the more similar their gut microbiome communities were (Fig. 3B; Mantel test UniFrac *p* = 0.0157, r = 0.218). This association was not detected in Asian elephants (Mantel test UniFrac *p* = 0.65).

When we held zoo environment constant (data subset to four zoo facilities that co-housed both African [n = 6] and Asian [n = 9] elephants), we still observed elephant species having dissimilar microbiome composition, but this depended on the zoo (Additional file 2: Fig. S5; PERMANOVA zoo by species interaction: Bray–Curtis Pseudo F_4,5_ = 1.55, R^2^ = 23.7%, *p* = 0.01; Jaccard Pseudo F_4,5_ = 1.35, R^2^ = 25.4%, *p* = 0.009; UniFrac Pseudo F_4,5_ = 1.56, R^2^ = 26%, *p* = 0.002). However, we found no effect of zoo, host species, or their interaction on bacterial ASV richness or phylogenetic diversity; African and Asian elephants co-housed at the five zoos had similar bacterial ASV richness and phylogenetic diversity (ANOVA bacterial ASV richness *p* > 0.05; phylogenetic diversity *p* > 0.05).

### Relationship between gut microbiome structure and clinical health concerns, reproductive hormones, and metabolic hormones

**Fig. 4:**
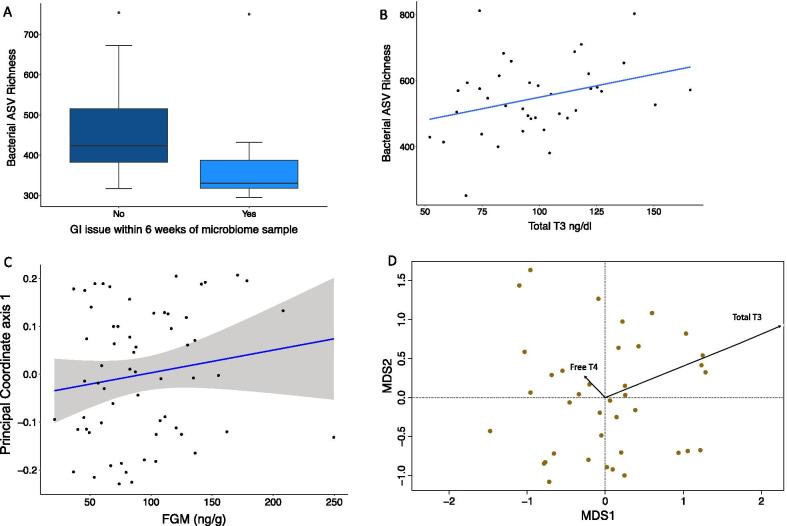
Associations of microbiome structure with GI issues and hormone concentrations in African and Asian elephants. **A** African elephants with recent GI issues had lower bacterial ASV richness (LMM *p* = 0.042, N = no GI issue [n = 83]; Y = GI issues 6 weeks prior to fecal collection [n = 8]). **B** In Asian elephants, bacterial richness increased with yearly averages of total T3 (Wald X2 *p* = 0.0441). **C** The gut microbiome composition of African elephants was linked to changes in FGM concentration. Principal coordinate axis 1 from Unifrac distances plotted against FGM concentrations. **D** Thyroid hormones, total T3 and free T4, were significantly associated with gut microbiome composition of Asian elephants. Vectors on the MDS plot depict the correlation of total T3 and free T4 with the pattern in ordination space using Bray–Curtis distances. The length of the vector signifies the strength of the correlation (e.g. shorter arrows represent weaker correlations)

In African elephants, we found recent GI issues resulted in lower bacterial ASV richness, and the gut microbiome composition was linked to specific reproductive and metabolic hormones. African elephants with recent GI issues had significantly lower bacterial ASV richness than elephants without a recent GI issue (Fig. 4A; Linear Mixed-effects Model [LMM] log transformed bacterial ASV richness, t_54.81_ =  − 2.082, *p* value = 0.042). We did not detect any significant relationships between hormones and health issues and bacterial phylogenetic diversity (LMM *p* value > 0.05). The African elephant gut microbiome composition (beta diversity) was linked to FGM using Unifrac measure, but not among other composition measures (Bray–Curtis, Jaccard) (Fig. 4C; results summarized in Table 3).

**Table 3:** Summary of PERMANOVA model statistics for all beta diversity measures (Bray Curtis, Jaccard, Unifrac) and clinical health issues, reproductive hormones, and metabolic hormones in African elephants

Variable	df	Bray–Curtis			Jaccard			Unifrac		
		R2	Pseudo F-stat	*p* value	R2	Pseudo F-stat	*p* value	R2	Pseudo F-stat	*p* value
Progestagens (ng/ml)	1	0.023	1.493	0.756	0.020	1.307	0.859	0.020	1.325	0.625
PRL (ng/ml)	1	0.026	1.718	0.401	0.022	1.388	0.383	0.027	1.809	0.516
LH (ng/ml)	1	0.020	1.310	0.488	0.020	1.270	0.114	0.020	1.313	0.207
FSH (ng/ml)	1	0.019	1.257	0.676	0.019	1.235	0.579	0.019	1.222	0.564
**FGM (ng/g)**	1	0.019	1.248	0.138	0.018	1.180	0.188	**0.019**	**1.229**	0.013*
Total T4 (ug/dl)	1	0.020	1.302	0.962	0.020	1.285	0.922	0.019	1.262	0.584
Total T3 (ng/dl)	1	0.027	1.797	0.994	0.025	1.571	0.989	0.026	1.736	0.946
Free T4 (ng/dl)	1	0.030	1.972	0.334	0.026	1.650	0.265	0.035	2.287	0.407
TSH (ng/ml)	1	0.020	1.300	0.974	0.019	1.217	0.972	0.021	1.357	0.608
BCS	2	0.041	1.329	0.101	0.038	1.197	0.157	0.041	1.345	0.160
GI_6wks	1	0.019	1.219	0.423	0.018	1.161	0.541	0.019	1.253	0.487
LameStiff_6wks	1	0.016	1.040	0.383	0.016	1.009	0.585	0.018	1.173	0.055
Age	1	0.017	1.095	0.644	0.017	1.110	0.389	0.019	1.234	0.440
Residual	46	0.703	NA	NA	0.721	NA	NA	0.698	NA	NA

Bold text indicates variables with significant *p* values

The Asian elephant gut microbiome was linked to metabolic hormones. We found bacterial ASV richness to be greater in elephants with higher yearly averages of total T3 (Fig. 4B; LM bacterial ASV richness *p* value = 0.0441, adjusted R^2^ = 8.3%). We did not detect any significant relationships between hormones and health issues and bacterial phylogenetic diversity (LM *p* value > 0.05). The Asian elephant gut microbiome composition was linked to total T3 and free T4 (Fig. 4D; Results summarized in Table 4).

**Table 4:** Summary of PERMANOVA model statistics for all beta diversity measures (Bray Curtis, Jaccard, Unifrac) and clinical health issues, reproductive hormones, and metabolic hormones in Asian elephants

Variable	df	Bray–Curtis			Jaccard			Unifrac		
		R2	Pseudo F-stat	*p* value	R2	Pseudo F-stat	*p* value	R2	Pseudo F-stat	*p* value
Progestagens (ng/ml)	1	0.025	1.027	0.689	0.027	1.087	0.351	0.026	1.050	0.597
PRL (ng/ml)	1	0.029	1.197	0.176	0.028	1.119	0.180	0.029	1.162	0.107
LH (ng/ml)	1	0.048	1.997	0.646	0.040	1.618	0.545	0.042	1.703	0.717
FSH (ng/ml)	1	0.030	1.254	0.833	0.029	1.155	0.830	0.028	1.143	0.768
FGM (ng/g)	1	0.043	1.803	0.664	0.036	1.449	0.716	0.034	1.372	0.902
Total T4 (ug/dl)	1	0.027	1.142	0.362	0.027	1.083	0.421	0.029	1.157	0.663
**Total T3 (ng/dl)**	**1**	**0.044**	**1.855**	0.004*	**0.040**	**1.610**	0.001*	**0.046**	**1.861**	0.006*
**Free T4 (ng/dl)**	**1**	**0.033**	**1.365**	0.006*	**0.030**	**1.223**	0.008*	0.031	1.244	0.830
TSH (ng/ml)	1	0.022	0.921	0.545	0.024	0.948	0.499	0.024	0.955	0.553
BCS	3	0.083	1.155	0.364	0.083	1.106	0.388	0.079	1.058	0.956
GI_6wks	1	0.030	1.243	0.082	0.029	1.181	0.053	0.026	1.054	0.363
LameStiff_6wks	1	0.032	1.327	0.382	0.031	1.226	0.214	0.032	1.292	0.163
Age	1	0.024	1.000	0.797	0.025	0.997	0.687	0.022	0.872	0.891
NSAID/AntiBio	1	0.031	1.293	0.468	0.029	1.169	0.589	0.029	1.186	0.661
Residual	21	0.501	NA	NA	0.523	NA	NA	0.522	NA	NA

Bold text indicates variables with significant *p* values

### Relationship between specific gut bacteria, clinical health concerns, reproductive hormones, and metabolic hormones

We were interested in linear relationships between bacterial ASV abundance and hormone concentrations that may be indicative of communication along the gut–brain axis. Using linear mixed effects models, we found significant relationships between bacterial ASVs and reproductive and metabolic hormones in African and Asian elephants. In African elephants, we found bacterial ASV abundances had significant linear relationships with PRL, FGM, LH, and FSH (Additional file 3: Figs. S6–S9; Additional file 4). We also found bacterial ASV abundances with significant relationships to clinical health concerns: BCS, lameness/stiffness, and age (Additional file 3: Figs. S10–S12; Additional file 4). In Asian elephants, we found significant linear relationships between bacterial ASV relative abundances and all reproductive and metabolic hormones and all clinical health concerns (Additional file 3: Figs. S13–S26; Additional file 5). Many relationships between ASVs and hormones were unique, though we did find certain bacterial ASVs with multiple associations to hormones and clinical health variables. Taxonomic levels with more than one association to a hormone or health variable included orders *Clostridiales* and *Cytophagales*, family *Rikenellaceae*, and genera *Prevotella* spp., *Paraprevotella* spp., *Porphyromonadaceae* spp., and *Treponema* spp.

**Fig. 5:**
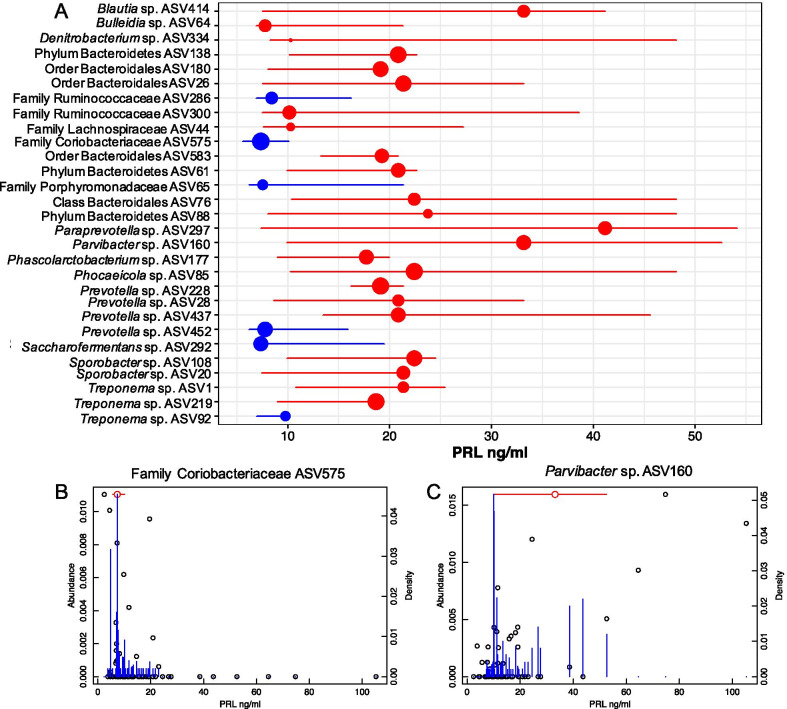
Threshold Indicator Taxa Analysis (TITAN) for bacterial taxa in response to prolactin concentrations. **A** We identified bacterial changepoints in association with prolactin (PRL) concentrations, with a collective positive changepoint identified at 17.7 ng/ml. Circles represent bacterial taxa changepoints with red circles representing a positive response to prolactin (z+) and blue circles representing a negative (z−) response to prolactin. The size of the circle reflects the magnitude of the Indicator Species Analysis statistic for the bacterial ASV. Horizontal lines represent the 5th and 95th percentiles among 500 bootstrap replicates. Examples of bacterial taxon abundance and density with a **B** negative response and **C** positive response to increasing prolactin concentrations. Blue lines represent bootstrap replicates for identifying change point. Red line indicates change point

We were also interested in non-linear relationships that may exist between hormone concentration and bacterial taxa. Threshold Indicator Taxa Analysis (TITAN) allowed us to identify (i) bacterial ASVs involved in non-linear relationships and (ii) pinpoint hormone concentrations where bacterial taxa abundance sharply increased or decreased. In African elephants, the relative abundance of several bacterial ASVs corresponded to progestagens, PRL, total T3, and free T4 thresholds (Additional file 2: Table S7; Additional file 3: Figs. S27–S30). The clearest change in positively associated bacterial abundance occurred at 17.7 ng/ml of prolactin (Fig. 5). In Asian elephants, we identified bacterial ASVs whose relative abundance corresponded to LH, FGM, total T3, and free T4 at certain thresholds (Additional file 2: Table S8; Additional file 3: Figs. S31–S34). Nine bacterial ASVs corresponded to hormone thresholds in both African and Asian elephants including genera *Paraprevotella* sp., *Prevotella* sp., *Rikenellaceae* sp., *Phocaeicola* spp., and *Treponema* spp. Within host species, certain bacterial ASVs corresponded to thresholds of at least two hormones. In African elephants these included ASVs from Phylum Candidatus *Saccharibacteria* and *Bacteroidetes*, order *Clostridiales*, and genera *Phocaeicola* sp. *Parvibacter* sp., *Holdemania* sp, *Prevotella* sp., *Ruminococcus* sp., *Sphaerochaeta* sp. and *Treponema* sp. In Asian elephants, specific ASVs in Phylum *Bacteroidetes*, family *Sphingobacteriaceae*, and genera *Dehalobacter* sp., and *Ruminococcus* sp. were associated to thresholds of at least two hormones.

## Discussion

Zoo managed animals provide an opportunity to better understand the basic biology of many species, yet individuals are often susceptible to health issues and low reproductive success. Studying the gut microbiome of captive animals in relation to health concerns is a necessary first step to evaluating the potential for incorporating microbial therapy into captive management. Additionally, captive animals serve as a comparative study system to further our understanding of host–microbiome relationships given the ability to re-sample the same individuals and availability of often extensive health and pedigree records [2, 4, 6]. This study explored the connection between the gut microbiome and health, reproductive, and metabolic concerns in zoo-managed female African and Asian elephants across North America. Our study shows that African and Asian elephants host distinct gut microbiome communities and demonstrates that zoo facility has a strong influence on the elephant gut microbiome community. These findings indicate that both host species-specific traits and environment strongly impact the gut microbiome of captive elephants. We found that associations of health issues and reproductive and metabolic hormones with gut microbiome structure differed between the two elephant species, indicating the importance of species-specific studies and the challenge to generalize broadly across species. However, we did find that some reproductive and metabolic hormones (PRL, LH, FSH, FGM) had linear relationships with specific bacterial taxa in both species. We also found certain genera (e.g. *Prevotealla* spp. and *Treponema* spp.) had relationships with multiple hormones. Relationships between bacterial taxa and FGM have been seen in other studies (gorilla and rhino) [3, 69], suggesting a more general connection between FGM and gut microbiota of captive mammals, while connections with reproductive hormones are less established in captive mammals and non-model systems.

African and Asian elephants harbored distinct bacterial communities, yet shared similar abundances of bacteria phyla. The similarity in phyla among the two host species likely reflects a similar diet among captive African and Asian elephants in North America, while the difference in bacterial species may reflect the influence of the host on bacterial selection. Host genetics, diet, and environment are all known to influence the gut microbiome; previous studies have demonstrated that host species influence microbial composition at finer scales of taxonomic ranks (family, genera, ASVs), while diet influences the abundance of phyla [70–73]. We show the gut microbiome of captive African and Asian elephants is dominated by four phyla: Bacteroidetes, Firmicutes, Spirochaetes, and Verrucomicrobia. Our study found a greater presence of Spirochaetes and Verrucomicrobia, and less Proteobacteria than previous studies on elephants and hind gut fermenters [3, 74–77]. The captive environment may contribute to these differences in dominant phyla, as Spirochaetes and Verrucomicrobia were more abundant in captive animals than wild animals [78]. Differences in the relative abundance of bacterial phyla have been hypothesized to be linked to dietary differences in wild African elephants (*L. africana* and *L.cyclosis*), with *L. cyclosis* eating more fruit and having an increase in Proteobacteria [74]. Wild African and Asian elephants are generally accepted to be mixed feeders, consuming both grasses and browse (shoots, forbs, twigs, and leaves), though season and location influence variations in diet [79–82]. In captivity, hay is the recommended primary food source for both host species [82], and we suggest the similar relative abundance of phyla observed in captive African and Asian elephants reflects this similar diet. Nonetheless, at finer taxonomic scales the gut microbiome differed greatly between the two species indicating that even with similar diets, host-specific factors are playing a strong role in regulating gut bacteria.

Zoo facility had the strongest effect in our study explaining ~ 62% of variation in microbiome composition in both African and Asian elephants. The majority of the captive elephant diet is based on hay, with hay type varying across geographic regions [82]. Therefore, we examined if there was a geographic pattern to the differences in gut microbiomes among zoos. We found that African elephants at zoos near one another had more similar gut microbiomes suggesting that climate or nutritional factors (hay type) may influence gut microbiome composition. However, we did not observe this pattern in Asian elephants; zoo facility still had a strong effect on gut microbiome composition, but did not show any geographic pattern. The similarity of zoo effects suggests that Asian and African elephants both show localized changes of the gut microbiome to particular environments, potentially through incorporating new microbes or microbial genes from the environment to adjust to dietary needs (aid in digestion of novel plants) [83–85]. However, the lack of a geographic pattern in Asian elephants suggests they may have relatively greater regulation of their gut microbiome and allow fewer environmental bacteria to be part of the community than African elephants. This could be due to African elephants being more sensitive to social stress in captivity than Asian elephants [42], which may interfere with host regulation over the gut microbiome [86]. Facility effects are common in microbiome studies [51, 87–89] and should be considered in study design and analysis of zoo animals.

The gut microbiome structure was associated to different health issues in African and Asian elephants. In African elephants, we found lower bacterial richness in elephants with a recent GI issues (previous 6 weeks) than those without, but we did not observe this in Asian elephants. Gastrointestinal issues (discomfort, colic, bloat, or abnormal feces) are the most prevalent clinical event in captive elephants, though Asian elephants are 65% less likely to experience GI issues [15]. African elephants have a shorter GI tract than most herbivores, including Asian elephants [90], and these differences in GI morphology may affect sensitivity to diets [2] and predisposition to GI issues in African elephants [15]. GI issues and microbiome community structure have been connected in other captive animals including doucs [2], red wolves [4], and horses [91]. Links between GI issue and microbiomes can result from a variety of issues including dysbiosis [91] or a lack of essential microbes needed for digestion [92]. In African elephants, we did not see shifts in gut microbiome composition typical of dysbiosis and do not believe that to be related to GI issues in this case. We suggest that a lack of specific microbes in certain individuals could be responsible for GI issues as certain digestive functions could be missing. If certain microbes and their digestive function are absent, parts of the elephant diet may be difficult to break down or their ability to cope with dietary changes may be more difficult. Fecal transplants may be a useful strategy from more bacterial rich and healthy individuals to individuals with GI issues to change the microbiome and accommodate a variety of diets [92] and warrants further study.

Metabolic hormones and gut microbiome structure were linked in Asian elephants, specifically total T3 and free T4. Total T3 and free T4 were associated to the gut bacterial composition of Asian elephants and total T3 was positively correlated with bacterial richness in Asian elephants. T3 and T4 work in concert to monitor metabolism, with greater levels thought to be indicative of higher metabolic rates [93–95]. Free T4 circulates unbound and moves to target organs where it is converted to T3 through deiodinase enzymes (Reviewed in Luongo et al.,) [96, 97]. Microbiota may be able to regulate this enzymatic transformation through binding T3 and T4 in the gut, preventing deiodinase activity [98], and thus influencing the abundance and activity of T3 and T4 in the body. The positive relationship between total T3 and bacterial richness in Asian elephants may reflect a relationship between energy intake and bacterial richness. However, a majority of elephants in our study were overweight (BCS > 4 & 5), thus thyroid hormone concentrations reflect those from overweight individuals. In human studies, increases in TSH and T3 have been linked to obesity [99], but the etiology is not well understood. Obese humans also have altered gut microbiome structure, with most studies showing a decrease in bacterial richness [100]. We did not find associations between BCS and bacterial richness or composition, though we found numerous relationships between BCS and specific bacterial ASV abundances that could be further investigated. Thus, more work is required to understand the interaction of metabolic hormones, energy intake and the gut microbiome in elephants. Our study used yearly averages of metabolic and reproductive hormones (excluding FGM) due to the data available from the IMLS study. Hormones fluctuate throughout reproductive cycles and in response to energy intake. We confirmed yearly averages correlated with daily values in a subset of our data (see Additional file 2). When possible, we suggest using daily values and to use care when interpreting data from yearly averages.

We found several bacterial taxa with linear and non-linear relationships with thyroid hormones. For example, *Treponema* sp. (ASV 139) had linear relationships with free T4 and total T3 in Asian elephants. *Treponema* spp. also had associations to reproductive hormones; *Treponema* sp. (ASV 219) had non-linear relationships with PRL in African elephants and LH in Asian elephants. These hormones are secreted from the anterior pituitary and regulate ovulation. Taken together, *Treponema* species were associated to both metabolic and reproductive hormones and could be of interest for future studies using genomic techniques to assess function. *Treponema* species are regularly found in animals gut microbiomes, including wild African elephants [74]. Likewise, *Treponema* sp. (ASV 1) was the most abundant ASV in our study. Bekele et al. found ruminant *Treponema* species to differ between diet, and suggest bacterial phylotypes may be diet specific [101]. Some *Treponema* species are pathogenic [102], thus requiring further work to understand the diversity and function of *Treponema* species in animal gut microbiomes. Our findings support the connection between thyroid hormones and microbiota, yet additional research is necessary to understand the intricacies of the gut–thyroid axis and the potential impacts for elephants.

The gut bacterial composition of African and Asian elephants was not connected to reproductive hormones, though we found the abundance of certain bacterial taxa had linear and non-linear relationships with reproductive hormones. We are in the early stages of understanding the complex nature of hormone–microbiota relationships, yet shifts in the gut microbiome associated to reproductive stage in eastern black rhinos [3] and Phayre’s leaf monkeys [103] suggest a link between gut bacterial composition and reproductive health across host species. Below, we highlight bacteria taxa that may be of interest in future studies regarding reproductive microbiomes including genera *Prevotella* (associated to both reproductive and metabolic hormones) and *Akkermansia* spp.

We found bacterial ASVs in the genera *Prevotella* were associated to reproductive and metabolic hormones in African and Asian elephants. For example, we found *Prevotella* sp. (ASV 28) had non-linear associations with PRL, progestagens, and total T3 in African elephants and with LH in Asian elephants. In African elephants, several other bacterial taxa in the *Prevotella* genus had non-linear relationships with progestagens, PRL, total T3, and free T4. Our results suggest *Prevotella* species may play an important role in reproduction and metabolism and could be a genus of interest for targeted research. Previous research has shown bacterial species in the *Prevotella* genus, specifically *Prevotella intermedius*, to take up estradiol and progesterone and further increase *Prevotella* abundance [104]. In southern white rhinos, *Prevotella* spp. were associated to decreased fertility [21], while in eastern black rhinos, breeding, pregnancy, and post-parturition were correlated to four other bacterial genera, though we did not detect these same genera in our study [3]. *Prevotella* spp. have also been associated to high carbohydrate diets [105], and are present in greater abundances in captive primates, at levels resembling human gut microbiomes [106]. Targeted research on *Prevotella* species could elucidate their relationship with reproductive and metabolic hormones by monitoring their abundance when making husbandry changes such as administering hormone contraceptives, diet changes, or exercise plans.

Bacteria associated to prolactin may be of interest in future studies due to the high incidence of hyperprolactinemia in captive African elephants in North America [14, 38, 43]. In African elephants, prolactin had linear correlations with 17 bacterial taxa and non-linear relationships with 31 bacterial taxa. We found two ASVs in the genus *Akkermansia* sp. (ASV 127; ASV 268) whose abundance was positively correlated with prolactin in African elephants, suggesting that *Akkermansia* spp. could be involved in mechanisms governing prolactin concentrations. *Akkermansia* species have been associated to GI issues in captive colobine primates, and *Akkermansia muciniphila* is recognized as a beneficial microbe in humans involved in immunity, metabolism, and prevention of obesity [107–110]. Further, *A. muciniphila* has been detected in higher abundances in females than males suggesting a possible connection with female specific hormones [111], such as prolactin. We did not find *A. muciniphila* species in elephants, thus further research is necessary to determine the identity and role of specific *Akkermansia* spp. detected in elephants. Specifically, if other *Akkermansia* spp. have similar roles in countering obesity, given the prevalence of obesity and hyperprolactinemia in the elephant zoo population and their connection to acyclicity. We also found numerous bacterial taxa with non-linear associations to prolactin and identified a sharp change in bacterial relative abundance at ~ 17 ng/ml of prolactin (see Fig. 5). This suggests there are increases in bacterial relative abundance in hyperprolactinemic elephants that are not associated with lower concentrations of prolactin (hyperprolactinemia categorized as 15 ng/ml or greater) [38, 112]. We do not know if bacterial abundance could contribute to the cause of hyperprolactinemia or be a by-product of it. The cause of hyperprolactinemia in elephants is unknown, making bacterial taxa associated to prolactin ideal for laboratory studies in model species to better understand these relationships.

Of the metabolic hormones included in our study, FGM has received the most attention in captive animals. FGMs are a measure of adrenal cortex activity and are frequently used to study stress in wildlife studies due to the non-invasive nature, though FGM should be interpreted cautiously [113, 114] due to the metabolic functions of glucocorticoids [45, 46] and non-stress related factors affecting FGM levels. Nevertheless, stress and gut microbiota are in communication with one another via the gut–brain axis, yet the mechanism is not well understood [7, 115]. This communication has been demonstrated in model species [116] and recently in wild and captive animals [3, 69]. For instance, correlations between bacterial abundance and FGM were observed in wild gorillas [69], while slight correlations were found with FGM in captive rhinos [3]. We found FGM was linked to gut bacterial composition in African elephants, and was associated to bacterial abundance in both African and Asian elephants. We found four bacterial taxa associated to FGM in African elephants and 16 in Asian elephants. FGM concentrations reflect recent glucocorticoid activity, indicating changes in FGM in African elephants can shift gut microbiome composition. In captive African elephants, increased FGM has been connected to social variables, such as mixed-sex herds (presence of a bull with females), while lower FGM and FGM variability has been associated to enrichment and choice of indoor and outdoor space [42]. Social variables influencing FGM may explain changes in the gut bacterial composition of African elephants and be important to African elephant gut bacterial composition, given FGM was the only significant variable linked to the African elephant gut bacterial composition.

## Conclusions

We present one of the first comprehensive examinations of linkages between the gut microbiome and clinical health variables, reproductive hormones and metabolic hormones in captive animals. Our findings underscore the strong combined effects of host species regulation and environmental effects on gut microbiomes. Our sampling across multiple zoo facilities is one of the most extensive to date for any captive animal species, and highlights the critical need to consider facility to facility variation in gut microbiome studies. Between elephant species, we recovered different patterns in the relationships between gut microbiome composition, health, and reproductive and metabolic issues, highlighting that even closely related species and their gut microbiomes respond to the captive environment in different ways. We suggest future studies use targeted fecal collection in accordance with common husbandry practices (i.e. contraceptive use, diet change, enrichment/exercise plan) to untangle directionality between gut microbiomes and reproductive and metabolic hormones. We identify certain bacterial taxa including genera *Prevotella*, *Treponema*, and *Akkermansia* that are ideal targets for follow-up studies using culturing methods and other genomic techniques to better understand their functions [117] and potential for creating probiotics that may someday be a tool for conservation and zoo management.

## Supplementary Information

**Additional file 1.** Pilot study methods, results, and figures: effects of lyophilization on fecal sample microbiome structure.

**Additional file 2.** Additional figures and tables.

**Additional file 3.** Linear and non-linear correlations of bacterial taxa relative abundance and reproductive and metabolic hormones in captive African and Asian elephants.

**Additional file 4.** Linear mixed model results for African elephants.

**Additional file 5.** Linear mixed model results for Asian elephants.

## Data Availability

The datasets generated and analyzed during the current study are available in the NCBI SRA repository (BioProject ID: PRJNA731172). Final files for analyses (feature table, taxonomy table, phylogenetic tree, and metadata file) and R code are available on figshare: https://doi.org/10.6084/m9.figshare.c.5433837.v3.

## Abbreviations

EWS: Elephant Welfare Study
SCBI-CSS: Smithsonian Conservation Biology Institute-Center for Species Survival
ASVs: Amplicon sequence variants
PCR: Polymerase chain reaction
HPA: Hypothalamic pituitary adrenal
LH: Luteal hormone
FSH: Follicular stimulating hormone
PRL: Prolactin
Total T4: Total thyroxine
Free T4: Free thyroxine
Total T3: Total triiodothyronine
TSH: Thyroid stimulating hormone
FGM: Fecal glucocorticoid metabolites
GI: Gastrointestinal
BCS: Body condition score
