# Supplementary material for: Clinical health issues, reproductive hormones, and metabolic hormones associated with gut microbiome structure in African and Asian elephants
Source: Anim Microbiome. 2021 Dec 20;3:85. doi: 10.1186/s42523-021-00146-9 (PMC8686393; doi:10.1186/s42523-021-00146-9)
Supplement: Supplementary file 1 — Additional file 1. Pilot study methods, results, and figures: effects of lyophilization on fecal sample microbiome structure. [file 42523_2021_146_MOESM1_ESM.docx]

**Additional File 1:** Pilot study methods, results, and figures: Effects of lyophilization on fecal sample microbiome structure

**This file includes:**

Supplementary methods for pilot study assessing effects of lyophilization on fecal microbiome samples

Supplementary results for pilot study on effects of lyophilization on fecal microbiome samples

Figure S1. Pilot study: Minimal effects of time and lyophilization on alpha diversity measures.

Figure S2. Pilot study: Microbial community identity remained across sample types.

Figure S3. Pilot study: Relative abundance plot depicting 16 bacterial phyla across sample type and time.

### Pilot study: Effect of Lyophilization on gut microbial communities

**METHODS**

We conducted a pilot study to assess the effects of lyophilization and time until

freezing on microbial communities. We collected one fecal from each Asian elephant at the National Zoo, Washington DC (n = 7). We collected samples (i) fresh (Fresh), (ii) fresh then lyophilized (LYO_0), (iii) 6 hours after collection then lyophilized (LYO_6), and (iv) 10 hours after collection then lyophilized (LYO_10). Animal keepers monitored elephants to document time of defecation and time of fecal bolus collection. Time of defecation to time of collection varied (12 -160 minutes) due to moving animals out of enclosure to access fecal bolus. We collected fresh and fresh to-be lyophilized samples by subsampling bolus immediately upon collection. We placed remaining fecal bolus in open, sanitized tubs and collected subsamples at designated study times (6 and 10 hours after collection). We kept samples on dry ice until transported by car to George Mason University – Science and Technology Campus where samples were stored at -20°C.

We extracted DNA and amplified the 16S rRNA gene following the same protocol outline in the ‘Molecular Methods and Sequencing’ section of this paper. We included a negative control in each set of extractions, as well a negative control with each set of PCR reactions. Zymobiomics microbial community DNA standard (Catalog No D6300; Zymo, USA) was included as a positive control when pooling samples. We performed a MiSeq run (2 x 300bp) on an Illumina MiSeq at the Evolutionary Genomics Lab, George Mason University. All data analysis was conducted in RStudio (v 1.1.463) for R (v3.5.2). We quality filtered data and identified unique bacterial taxa referred to as amplicon sequence variants using dada2 package (ASVs; Callahan et al., 2016, Callahan et al., 2017). Taxonomy was assigned to ASVs using Ribosomal Database Project classifier (RDP; Wang et al., 2007). A phylogenetic tree was built from bacterial ASVs in QIIME2 (Bolyen et al., 2018). We exported ASVs, the taxonomy table, and the meta data to be used in the phyloseq package for further analysis (McMurdie and Holmes, 2013). We filtered 14 potential contaminants using decontam package (v1.1.2) with the combined method which assesses frequency and prevalence of DNA (Davis et al., 2018). We removed singletons from our dataset prior to further analysis.

We assessed differences between sample types and time until freezing with alpha and beta diversity measures and compared relative abundance of dominant bacterial phyla. Alpha diversity measures, bacterial ASV richness and phylogenetic diversity, were used as response variables in a Bayesian linear mixed model (blmer function; blme package) with sample type as an explanatory variable and animal ID as a random effect (Chung et al., 2013). We compared alpha diversity models to a null model using ANOVA and conducted a pairwise post hoc test using package ‘emmeans’ (Lenth, 2020). Beta diversity measures, Bray-Curtis, Jaccard and UniFrac, were used as response variables in a PERMANOVA with sample type and animal ID as explanatory variables. Lastly, we tested for differential abundance in bacterial phyla among sample types. We used function *testDA* (package DAtest) to identify methods with the lowest false positive rates for our dataset (Russel et al 2018). We reported bacterial phyla that were significant in at least two of the three methods: Linear regression (*DA.lma* function), ANOVA (*DA.aoa* function), and Quasi-poisson generalized linear model (*DA.qpo* function). Post-hoc tests were conducted to determine which groups significantly differed from one another using functions *DA.lsmeans* for linear models (lma & qpo) and *DA.TukeyHSD* for ANOVA (aoa).

**RESULTS**

#### Effect of lyophilization on fecal microbial communities

In our pilot study we found that lyophilized samples, regardless of time until freezing, did not dramatically differ from fresh samples in microbiome diversity structure. We compared fresh samples to lyophilized samples with a range of times until freezing (sample types: (i) fresh and frozen, (ii) fresh, frozen and lyophilized, (iii) freeze after 6 hours and lyophilized, (iv) freeze after 10 hours and lyophilized). We found fresh samples and lyophilized samples generally had similar alpha diversity within individual elephants: (i) bacterial ASV richness was similar among samples within an individual, except the collection at 6 hours and lyophilized (LYO_6) had greater species richness than fresh samples (Figure S1a; EMM p = 0.028), and (ii) phylogenetic diversity was similar among all sample types (Figure S1b; ANOVA model comparison p >0.05). Fresh samples and lyophilized samples did not differ from one another in bacterial composition (Figure S2; beta diversity: PERMANOVA Bray-Curtis, Jaccard, and UniFrac p > 0.05), and explained a small percentage of the variation in composition (7.6%, 8.4% and 9.3%, Bray-Curtis, Jaccard, and UniFrac respectively); individual identity was retained in all sample types, regardless of sample processing. Gut bacterial composition differed among individuals (Figure 1; PERMANOVA Bray-Curtis, Jaccard, and UniFrac p = 0.001), with individual identity explaining 78.6%, 58%, and 53.3% of compositional variation respectively. Bacterial phyla abundance remained relatively even among sample types, however we found Phylum Lentisphaerae was significantly more abundant in lyophilized samples compared to fresh samples (Figure S3; *DA.lma*, *DA.aoa*, and *DA.qpo* p < 0.014). Other phyla marginally increased or decreased across sample types, including Candidatus Saccharibacteria, Planctomycetes, Fibrobacteres and Synergistetes, though were not significant in two out of three tests. Future use of lyophilized samples should consider the potential influence lyophilization has on the abundance of certain bacterial phyla. We concluded individual identity remained in lyophilized fecal samples and provided support for us to use the EWS samples. This conclusion is further supported by a study by Blekhman et al., (2016), who also found individual signature remained in lyophilized fecal samples.

**Supplemental Figures**

**
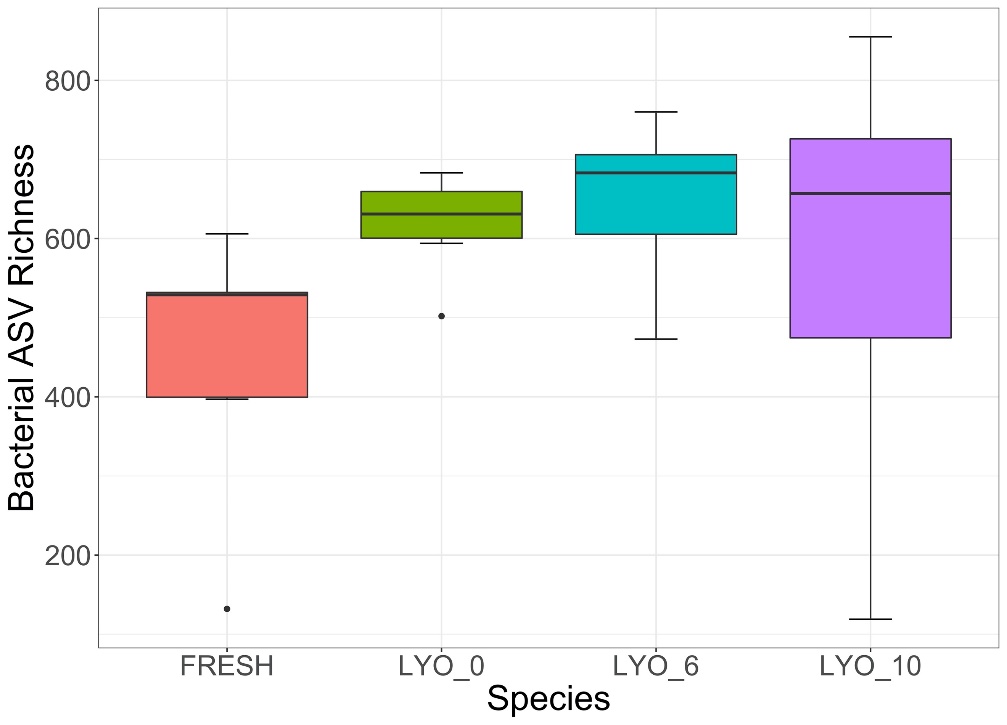

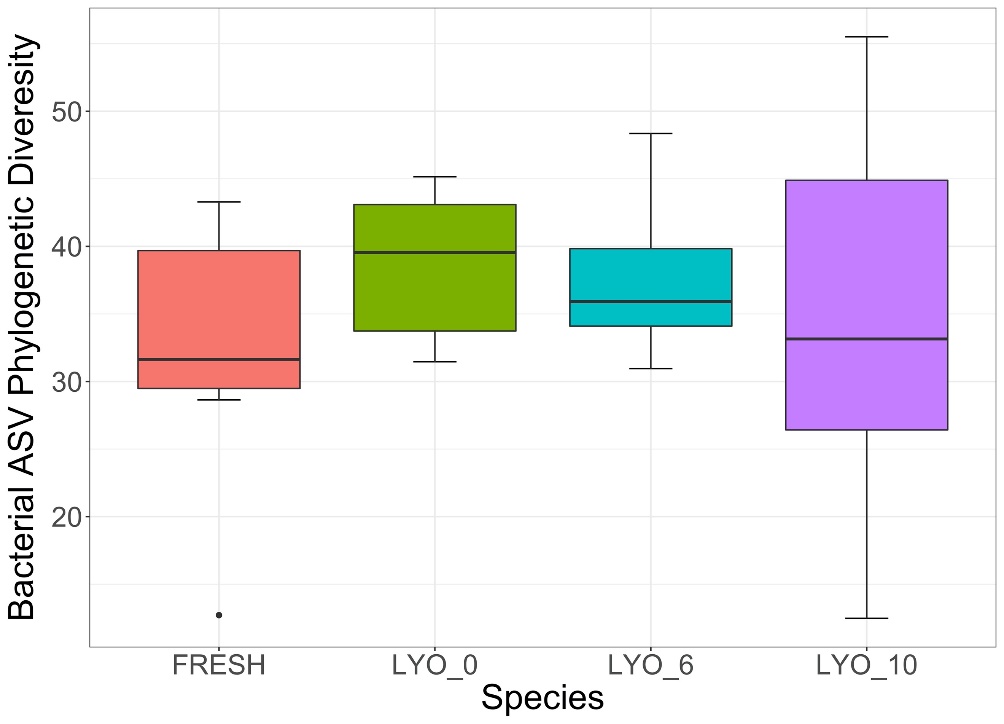
**

**Figure S1. Pilot study: Minimal effects of time and lyophilization on alpha diversity measures. (A)** Bacterial ASV richness was similar among sample types, except samples collected at 6 hours and lyophilized (LYO_6) had greater species richness than fresh samples (ANOVA p = 0.025; EMMs p = 0.028). **(B)** Bacterial ASV phylogenetic diversity was similar among all sample types (ANOVA p = 0.5891).

Figure S2. Pilot Study: Microbial community identity remained across sample types.

No significant differences across sample types supported the use of previously lyophilized samples from EWS for microbiome analysis in our main study (PERMANOVA Bray-Curtis p > 0.05). Sample types were as follows: ‘FRESH’ refers to fecal samples frozen after collection; LYO 0, LYO 6, and LYO 10 refer to the number of hours samples sat out until frozen, followed by lyophilization.

**Figure S3. Pilot study: Relative abundance plot depicting bacterial phyla across sample type and time.** ‘FRESH’ refers to fecal samples frozen after collection. LYO 0, LYO 6, and LYO 10 refer to the number of hours samples sat out until frozen, followed by lyophilization. We found Phylum Lentisphaerae was more abundant in lyophilized samples than fresh samples (using package DAtest) (Russel et al 2018).

References:

Chung Y, Rabe-Hesketh S, Dorie V, Gelman A, Liu J. A nondegenerate penalized likelihood estimator for variance parameters in multilevel models. Psychometrika. 2013;78(4):685–709.

Lenth R. emmeans: Estimated Marginal Means, aka Least-Squares Means. R package version 1.47. 2020.

Russel J, Thorsen J, Brejnrod AD, Bisgaard H, Sørensen SJ, Burmølle M. DAtest: a framework for choosing differential abundance or expression method. bioRxiv. 2018.
