# Supplementary material for: Clinical health issues, reproductive hormones, and metabolic hormones associated with gut microbiome structure in African and Asian elephants
Source: Anim Microbiome. 2021 Dec 20;3:85. doi: 10.1186/s42523-021-00146-9 (PMC8686393; doi:10.1186/s42523-021-00146-9)
Supplement: Supplementary file 2 — Additional file 2. Additional figures and tables. [file 42523_2021_146_MOESM2_ESM.docx]

**Additional file 2:** Supplementary figures and tables

**This file contains:**

Figure S4. Rarefaction plot depicting number of ASVs per African and Asian elephant samples

Figure S5. Host species differed in gut microbiome composition depending on zoo

Table S1. Correlations between day and yearly averages of serum hormone concentrations

Table S2. Forward and reverese primers used to amplify the 16S rRNA gene

Table S 3. Core gut microbiome bacterial ASVs of captive female African elephant (in >80% of samples)

Table S4. Core gut microbiome bacterial ASVs of captive female Asian elephant (in >80% of samples)

Table S5. Summary of LMM model statistics for alpha diversity measures and clinical health issues, reproductive hormones, and metabolic hormones in African elephants

Table S6. Summary of LMM model statistics for alpha diversity measures and clinical health issues, reproductive hormones, and metabolic hormones in Asian elephants

Table S7. Non-linear relationships from Threshold Indicator Taxa Analysis (TITAN) between bacterial ASV relative abundance and reproductive and metabolic hormones in African elephants

Table S8. Non-linear relationships from Threshold Indicator Taxa Analysis (TITAN) between bacterial ASV relative abundance and reproductive and metabolic hormones in Asian elephants

**
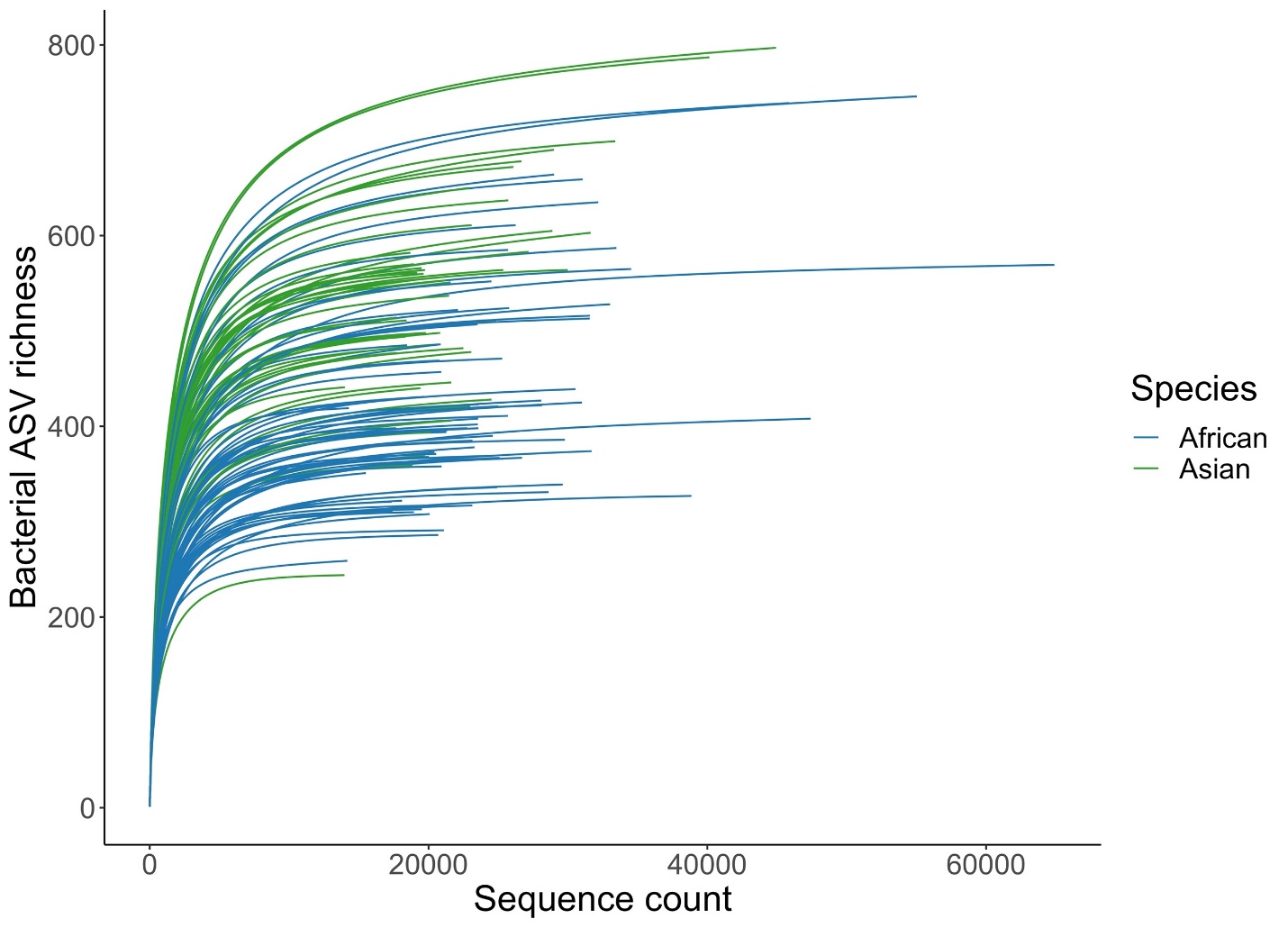
**

**Figure S4. Rarefaction plot depicting number of ASVs per African and Asian elephant samples.** Rarefaction plot depicts adequate sequence coverage for each sample with average sequence reads of 24,478 sequences per sample.

**Figure S5. Host species differed in gut microbiome composition depending on zoo**.

Bray Curtis PCoA plot highlighting zoo facilities co-housing African and Asian elephants.

(PERMANOVA Bray-Curtis zoo by species interaction F_4,5_ = 1.55, p = 0.001; R^2^ = 23.7%; African n = 6; Asian n = 9).

**Table S1. Correlations between day and yearly averages of serum hormone concentrations.** We found day hormone values significantly correlated to yearly hormone averages in a subset of our data (African elephants n = 34 and Asian elephants n = 17). We tested the strength of association using Pearson’s correlation coefficient (*cor.test* function, stats package).

| African Elephants | | |
| --- | --- | --- |
| Hormone | p-value | Pearson’s correlation coefficient |
| PRL | < 0.001 | 0.908 |
| Progestagen | 0.04 | 0.345 |
| LH | 0.002 | 0.497 |
| FSH | < 0.001 | 0.81 |
| T3 | < 0.001 | 0.76 |
| T4 | < 0.001 | 0.84 |
| TSH | < 0.001 | 0.851 |
| Asian Elephants | | |
|  | p-value | Pearson’s correlation coefficient |
| PRL | 0.027 | 0.535 |
| Progestagen | 0.005 | 0.648 |
| LH | < 0.001 | 0.775 |
| FSH | <0.001 | 0.777 |
| T3 | <0.001 | 0.933 |
| T4 | <0.001 | 0.829 |
| TSH | <0.001 | 0.769 |

Table S2. Forward and reverese primers used to amplify the 16S rRNA gene. Bolded text represets the sequencing primer binding site.

| Primer | Sequence |
| --- | --- |
| I2S-515F (Forward) | TCGTCGGCAGCGTC**AGATGTGTATAAGAGACAG**GTGYCAGCMGCCGCGGTAA |
| 12S-939R (Reverse) | GTCTCGTGGGCTCGG**AGATGTGTATAAGAGACAG**CTTGTGCGGGCCCCCGTCAATTC |

Table S 3. Core gut microbiome bacterial ASVs of captive female African elephant (in >80% of samples)

| ASV | Phylum | Class | Order | Family | Genus | % AVG RA | % SD RA |
| --- | --- | --- | --- | --- | --- | --- | --- |
| ASV2 | Firmicutes | Erysipelotrichia | Erysipelotrichales | Erysipelotrichaceae | *Faecalitalea* | 1.97 | 3.12 |
| ASV31 | Verrucomicrobia | Subdivision5 | -- | -- | *--* | 0.83 | 0.75 |
| ASV15 | Firmicutes | Clostridia | Clostridiales | Ruminococcaceae | *Sporobacter* | 0.80 | 1.03 |
| ASV1 | Spirochaetes | Spirochaetia | Spirochaetales | Spirochaetaceae | *Treponema* | 0.61 | 0.93 |
| ASV178 | Proteobacteria | Deltaproteobacteria | Desulfovibrionales | Desulfovibrionaceae | *Bilophila* | 0.24 | 0.38 |
| ASV64 | Firmicutes | Erysipelotrichia | Erysipelotrichales | Erysipelotrichaceae | *Bulleidia* | 0.24 | 0.30 |
| ASV197 | Verrucomicrobia | Subdivision5 | -- | -- | *--* | 0.24 | 0.27 |

**Table S4. Core gut microbiome bacterial ASVs of captive female Asian elephant (in >80% of samples)**

| ASV | Phylum | Class | Order | Family | Genus | % Avg RA | % SD RA |
| --- | --- | --- | --- | --- | --- | --- | --- |
| ASV1 | Spirochaetes | Spirochaetia | Spirochaetales | Spirochaetaceae | *Treponema* | 2.12 | 2.16 |
| ASV6 | Bacteroidetes | Bacteroidia | Bacteroidales | Prevotellaceae | *--* | 1.36 | 1.41 |
| ASV5 | Spirochaetes | Spirochaetia | Spirochaetales | Spirochaetaceae | *Treponema* | 1.29 | 1.49 |
| ASV29 | Synergistetes | Synergistia | Synergistales | Synergistaceae | *--* | 0.84 | 0.92 |
| ASV2 | Firmicutes | Erysipelotrichia | Erysipelotrichales | Erysipelotrichaceae | *Faecalitalea* | 0.77 | 1.69 |
| ASV80 | Bacteroidetes | Bacteroidia | Bacteroidales | -- | *--* | 0.59 | 0.69 |
| ASV47 | Verrucomicrobia | Subdivision5 | -- | -- | *--* | 0.58 | 0.36 |
| ASV11 | Bacteroidetes | Bacteroidia | Bacteroidales | Prevotellaceae | *Paraprevotella* | 0.54 | 0.61 |
| ASV20 | Firmicutes | Clostridia | Clostridiales | Ruminococcaceae | *Sporobacter* | 0.47 | 0.36 |
| ASV41 | Bacteroidetes | Bacteroidia | Bacteroidales | -- | *--* | 0.39 | 0.92 |
| ASV54 | Firmicutes | Clostridia | Clostridiales | Ruminococcaceae | *--* | 0.38 | 0.26 |
| ASV37 | Verrucomicrobia | Subdivision5 | -- | -- | *--* | 0.37 | 0.29 |
| ASV147 | Firmicutes | Clostridia | Clostridiales | Ruminococcaceae | *--* | 0.35 | 0.35 |
| ASV64 | Firmicutes | Erysipelotrichia | Erysipelotrichales | Erysipelotrichaceae | *Bulleidia* | 0.35 | 0.40 |
| ASV52 | Firmicutes | Negativicutes | Selenomonadales | Acidaminococcaceae | *Phascolarcto- bacterium* | 0.34 | 0.22 |
| ASV40 | Verrucomicrobia | Subdivision5 | -- | -- | *--* | 0.32 | 0.27 |
| ASV101 | Firmicutes | Clostridia | Clostridiales | Lachnospiraceae | *--* | 0.29 | 0.21 |
| ASV112 | Spirochaetes | Spirochaetia | Spirochaetales | Spirochaetaceae | *Treponema* | 0.29 | 0.23 |
| ASV21 | Firmicutes | Clostridia | Clostridiales | -- | *--* | 0.28 | 0.27 |
| ASV140 | Verrucomicrobia | Subdivision5 | -- | -- | *--* | 0.25 | 0.19 |
| ASV237 | Planctomycetes | Planctomycetia | Planctomycetales | Planctomycetaceae | *--* | 0.24 | 0.26 |
| ASV44 | Firmicutes | Clostridia | Clostridiales | Lachnospiraceae | *--* | 0.22 | 0.26 |
| ASV73 | Bacteroidetes | Bacteroidia | Bacteroidales | -- | *--* | 0.22 | 0.20 |
| ASV220 | Firmicutes | Clostridia | Clostridiales | Lachnospiraceae | *Clostridium_XlVa* | 0.20 | 0.20 |
| ASV83 | Bacteroidetes | Bacteroidia | Bacteroidales | -- | *--* | 0.19 | 0.21 |
| ASV189 | Verrucomicrobia | Subdivision5 | -- | -- | *--* | 0.18 | 0.22 |
| ASV139 | Spirochaetes | Spirochaetia | Spirochaetales | Spirochaetaceae | *Treponema* | 0.18 | 0.17 |
| ASV222 | Firmicutes | Clostridia | Clostridiales | Clostridiales_Incertae_  Sedis_XIII | *Anaerovorax* | 0.16 | 0.13 |
| ASV150 | Bacteroidetes | Bacteroidia | Bacteroidales | Rikenellaceae | *Rikenella* | 0.15 | 0.14 |
| ASV260 | Bacteroidetes | Bacteroidia | Bacteroidales | Prevotellaceae | *Paraprevotella* | 0.10 | 0.08 |
| ASV689 | Firmicutes | Clostridia | Clostridiales | Ruminococcaceae | *Sporobacter* | 0.08 | 0.06 |
| ASV741 | Firmicutes | Clostridia | Clostridiales | Ruminococcaceae | *--* | 0.05 | 0.06 |

**Table S5. Summary of LMM model statistics for alpha diversity measures and clinical health issues, reproductive hormones, and metabolic hormones in African elephants.** Backward model selection was used to identify the final model.

| Full Model: | lmer(Alpha~ P4 + PRL + LH + FSH + FecalCort + total t4 + total T3 + free t4 + TSH + BCS + GI_6wks + LameStiff_6wks + Age + (1\|ZooID), REML = F) |
| --- | --- |

| Final Model: | lmer(SRlog ~ GI_6wks + (1\|ZooID) | | | |
| --- | --- | --- | --- | --- |
| **Species Richness (SR)** |  | | | |
| *Explanatory Variables* | *Eliminated* | *df* | *F value* | *p value* |
| Progestagens (ng/ml) | 1 | 1, 50.095 | 0.028 | 0.868 |
| Age | 2 | 1, 47.175 | 0.188 | 0.666 |
| Free T4 (ng/dl) | 3 | 1, 60.983 | 0.337 | 0.564 |
| BCS | 4 | 2, 41.801 | 0.686 | 0.509 |
| FSH (ng/ml) | 5 | 1, 46.817 | 0.479 | 0.492 |
| TSH (ng/ml) | 6 | 1, 51.350 | 0.738 | 0.394 |
| LameStiff_6wks | 7 | 1, 47.900 | 0.690 | 0.410 |
| FGM (ng/g) | 8 | 1, 48.134 | 0.878 | 0.353 |
| Total T4 (ug/dl) | 9 | 1, 54.542 | 1.034 | 0.314 |
| Total T3 (ng/dl) | 10 | 1, 60.723 | 1.742 | 0.192 |
| LH (ng/ml) | 11 | 1, 56.670 | 1.639 | 0.206 |
| PRL (ng/ml) | 12 | 1, 52.763 | 1.884 | 0.176 |
| **GI_6wks** | **0** | **1, 54.809** | **4.336** | **0.042*** |

| Final Model: | lmer(PD ~ (1\|ZooID) | | | |
| --- | --- | --- | --- | --- |
| **Phylogenetic Diversity (PD)** |  | | | |
| *Explanatory Variables* | *Eliminated* | *df* | *F value* | *p value* |
| Free T4 (ng/dl) | 1 | 1, 55.084 | 0.001 | 0.979 |
| LH (ng/ml) | 2 | 1, 60.859 | 0.007 | 0.936 |
| TSH (ng/ml) | 3 | 1, 54.924 | 0.005 | 0.946 |
| Total T4 (ug/dl) | 4 | 1, 54.635 | 0.013 | 0.911 |
| FGM (ng/g) | 5 | 1, 50.053 | 0.022 | 0.882 |
| Progestagens (ng/ml) | 6 | 1, 47.552 | 0.101 | 0.752 |
| LameStiff_6wks | 7 | 1, 49.419 | 0.238 | 0.628 |
| PRL (ng/ml) | 8 | 1, 51.571 | 0.687 | 0.411 |
| Total T3 (ng/dl) | 9 | 1, 60.216 | 0.670 | 0.416 |
| GI_6wks | 10 | 1, 48.542 | 1.160 | 0.287 |
| BCS | 11 | 2, 42.478 | 1.233 | 0.302 |
| Age | 12 | 1, 46.762 | 2.252 | 0.140 |
| FSH (ng/ml) | 13 | 1, 52.207 | 3.213 | 0.079 |

**Table S6. Summary of LMM model statistics for alpha diversity measures and clinical health issues, reproductive hormones, and metabolic hormones in Asian elephants.** Backward model selection was used to identify the final model.

| Full Model: | lmer(SR ~ P4.ng.ml +PRL.ng.ml + LH.ng.ml + FSH.ng.ml + Fecal.Cort.day.ng.g + tot.T4.µg.dl + tot.t3.ng.dl + free.t4.ng.dl + TSH.ng.ml + BCS + GI_6wks + LameStiff_6wks + Age.July.2012 + NSAID_Anti + (1\|ZooID), |
| --- | --- |

| Final Model: | lmer(SR ~ tot.t3.ng.dl) | | | |
| --- | --- | --- | --- | --- |
| **Species Richness (SR)** |  | | | |
| *Explanatory Variables* | *Eliminated* | *df* | *F value* | *p value* |
| GI_6wks | 1 | 1 | 0.020 | 0.890 |
| TSH (ng/ml) | 2 | 1 | 0.047 | 0.830 |
| LameStiff_6wks | 3 | 1 | 0.261 | 0.615 |
| Progestagens (ng/ml) | 4 | 1 | 0.169 | 0.685 |
| LH (ng/ml) | 5 | 1 | 0.295 | 0.592 |
| BCS | 6 | 3 | 0.686 | 0.568 |
| FGM (ng/g) | 7 | 1 | 0.607 | 0.442 |
| NSAIDs & Antibiotics | 8 | 1 | 0.923 | 0.344 |
| Total T4 (ug/dl) | 9 | 1 | 1.425 | 0.242 |
| Age | 10 | 1 | 1.039 | 0.316 |
| Free T4 (ng/dl) | 11 | 1 | 1.297 | 0.263 |
| PRL (ng/ml) | 12 | 1 | 1.971 | 0.169 |
| FSH (ng/ml) | 13 | 1 | 3.616 | 0.065 |
| **Total T3 (ng/dl)** | 0 | 1 | 4.351 | **0.044*** |

| Final Model | PD ~ 1 | | | |
| --- | --- | --- | --- | --- |
| **Phylogenetic Diveresity (PD)** |  | | | |
| *Explanatory Variables* | *Eliminated* | *df* | *F value* | *p value* |
| LameStiff_6wks | 1 | 1 | 0.008 | 0.931 |
| LH (ng/ml) | 2 | 1 | 0.044 | 0.836 |
| TSH (ng/ml) | 3 | 1 | 0.083 | 0.776 |
| Total T4 (ug/dl) | 4 | 1 | 0.462 | 0.503 |
| Free T4 (ng/dl) | 5 | 1 | 0.195 | 0.663 |
| PRL (ng/ml) | 6 | 1 | 0.419 | 0.523 |
| FGM (ng/g) | 7 | 1 | 0.359 | 0.554 |
| BCS | 8 | 3 | 1.082 | 0.373 |
| NSAIDs & Antibiotics | 9 | 1 | 0.616 | 0.439 |
| GI_6wks | 10 | 1 | 0.111 | 0.741 |
| FSH (ng/ml) | 11 | 1 | 0.810 | 0.375 |
| **Progestagens (ng/ml)** | 12 | 1 | 3.094 | 0.088 |
| Age | 13 | 1 | 2.516 | 0.122 |
| Total T3 (ng/dl) | 14 | 1 | 3.863 | 0.057 |

**Table S7. Non-linear relationships from Threshold Indicator Taxa Analysis (TITAN) between bacterial ASV relative abundance and reproductive and metabolic hormones in African elephants**

| Taxonomy Assignment | Hormone |
| --- | --- |
| Bacteroidetes_Bacteroidia_Bacteroidales_NA_NA_ASV304 | P4 |
| Bacteroidetes_NA_NA_NA_NA_ASV440 | P4 |
| Bacteroidetes_Bacteroidia_Bacteroidales_Bacteroidales_incertae_sedis_Phocaeicola_ASV85 | P4 |
| Bacteroidetes_Bacteroidia_Bacteroidales_NA_NA_ASV76 | P4 |
| Bacteroidetes_Bacteroidia_Bacteroidales_Prevotellaceae_Prevotella_ASV383 | P4 |
| Bacteroidetes_Bacteroidia_Bacteroidales_Prevotellaceae_Prevotella_ASV452 | P4 |
| Bacteroidetes_Bacteroidia_Bacteroidales_Prevotellaceae_Prevotella_ASV28 | P4 |
| Candidatus_Saccharibacteria_NA_NA_NA_NA_ASV472 | P4 |
| Candidatus_Saccharibacteria_NA_NA_NA_NA_ASV249 | P4 |
| Firmicutes_Clostridia_Clostridiales_Ruminococcaceae_Ruminococcus_ASV356 | P4 |
| Firmicutes_Clostridia_Clostridiales_Ruminococcaceae_Oscillibacter_ASV321 | P4 |
| Spirochaetes_Spirochaetia_Spirochaetales_Spirochaetaceae_Treponema_ASV479 | P4 |
| Spirochaetes_Spirochaetia_Spirochaetales_Spirochaetaceae_Sphaerochaeta_ASV330 | P4 |
| Synergistetes_Synergistia_Synergistales_Synergistaceae_NA_ASV193 | P4 |
| Synergistetes_Synergistia_Synergistales_Synergistaceae_Synergistes_ASV442 | P4 |
| Verrucomicrobia_Verrucomicrobiae_Verrucomicrobiales_Verrucomicrobiaceae_Akkermansia_ASV268 | P4 |
| Verrucomicrobia_Subdivision5_NA_NA_NA_ASV31 | P4 |
| Actinobacteria_Actinobacteria_Coriobacteriales_Coriobacteriaceae_Parvibacter_ASV160 | PRL |
| Actinobacteria_Actinobacteria_Coriobacteriales_Coriobacteriaceae_NA_ASV575 | PRL |
| Actinobacteria_Actinobacteria_Coriobacteriales_Coriobacteriaceae_Denitrobacterium_ASV334 | PRL |
| Bacteroidetes_Bacteroidia_Bacteroidales_NA_NA_ASV583 | PRL |
| Bacteroidetes_NA_NA_NA_NA_ASV138 | PRL |
| Bacteroidetes_NA_NA_NA_NA_ASV61 | PRL |
| Bacteroidetes_NA_NA_NA_NA_ASV88 | PRL |
| Bacteroidetes_Bacteroidia_Bacteroidales_Prevotellaceae_Paraprevotella_ASV297 | PRL |
| Bacteroidetes_Bacteroidia_Bacteroidales_Bacteroidales_incertae_sedis_Phocaeicola_ASV85 | PRL |
| Bacteroidetes_Bacteroidia_Bacteroidales_NA_NA_ASV26 | PRL |
| Bacteroidetes_Bacteroidia_Bacteroidales_NA_NA_ASV76 | PRL |
| Bacteroidetes_Bacteroidia_Bacteroidales_Prevotellaceae_Prevotella_ASV437 | PRL |
| Bacteroidetes_Bacteroidia_Bacteroidales_Prevotellaceae_Prevotella_ASV228 | PRL |
| Bacteroidetes_Bacteroidia_Bacteroidales_Prevotellaceae_Prevotella_ASV452 | PRL |
| Bacteroidetes_Bacteroidia_Bacteroidales_Prevotellaceae_Prevotella_ASV28 | PRL |
| Bacteroidetes_Bacteroidia_Bacteroidales_Porphyromonadaceae_NA_ASV65 | PRL |
| Bacteroidetes_Bacteroidia_Bacteroidales_NA_NA_ASV180 | PRL |
| Firmicutes_Erysipelotrichia_Erysipelotrichales_Erysipelotrichaceae_Holdemania_ASV376 | PRL |
| Firmicutes_Erysipelotrichia_Erysipelotrichales_Erysipelotrichaceae_Bulleidia_ASV64 | PRL |
| Firmicutes_Negativicutes_Selenomonadales_Acidaminococcaceae_Phascolarctobacterium_ASV177 | PRL |
| Firmicutes_Clostridia_Clostridiales_Ruminococcaceae_Saccharofermentans_ASV292 | PRL |
| Firmicutes_Clostridia_Clostridiales_Ruminococcaceae_NA_ASV286 | PRL |
| Firmicutes_Clostridia_Clostridiales_Ruminococcaceae_NA_ASV300 | PRL |
| Firmicutes_Clostridia_Clostridiales_Ruminococcaceae_Sporobacter_ASV20 | PRL |
| Firmicutes_Clostridia_Clostridiales_Ruminococcaceae_Sporobacter_ASV108 | PRL |
| Firmicutes_Clostridia_Clostridiales_Lachnospiraceae_Blautia_ASV414 | PRL |
| Firmicutes_Clostridia_Clostridiales_Lachnospiraceae_NA_ASV44 | PRL |
| Spirochaetes_Spirochaetia_Spirochaetales_Spirochaetaceae_Treponema_ASV92 | PRL |
| Spirochaetes_Spirochaetia_Spirochaetales_Spirochaetaceae_Treponema_ASV1 | PRL |
| Spirochaetes_Spirochaetia_Spirochaetales_Spirochaetaceae_Treponema_ASV5 | PRL |
| Spirochaetes_Spirochaetia_Spirochaetales_Spirochaetaceae_Treponema_ASV219 | PRL |
| Actinobacteria_Actinobacteria_Coriobacteriales_Coriobacteriaceae_Parvibacter_ASV160 | Total T3 |
| Bacteroidetes_Bacteroidia_Bacteroidales_Porphyromonadaceae_NA_ASV234 | Total T3 |
| Bacteroidetes_Bacteroidia_Bacteroidales_Porphyromonadaceae_NA_ASV329 | Total T3 |
| Bacteroidetes_NA_NA_NA_NA_ASV138 | Total T3 |
| Bacteroidetes_NA_NA_NA_NA_ASV61 | Total T3 |
| Bacteroidetes_Bacteroidia_Bacteroidales_Prevotellaceae_Paraprevotella_ASV126 | Total T3 |
| Bacteroidetes_Bacteroidia_Bacteroidales_Prevotellaceae_Paraprevotella_ASV11 | Total T3 |
| Bacteroidetes_Bacteroidia_Bacteroidales_Bacteroidales_incertae_sedis_Phocaeicola_ASV94 | Total T3 |
| Bacteroidetes_Bacteroidia_Bacteroidales_Prevotellaceae_NA_ASV600 | Total T3 |
| Bacteroidetes_Bacteroidia_Bacteroidales_Prevotellaceae_Prevotella_ASV241 | Total T3 |
| Bacteroidetes_Bacteroidia_Bacteroidales_Prevotellaceae_Prevotella_ASV199 | Total T3 |
| Bacteroidetes_Bacteroidia_Bacteroidales_Prevotellaceae_Prevotella_ASV207 | Total T3 |
| Bacteroidetes_Bacteroidia_Bacteroidales_Prevotellaceae_Prevotella_ASV562 | Total T3 |
| Bacteroidetes_Bacteroidia_Bacteroidales_NA_NA_ASV398 | Total T3 |
| Bacteroidetes_Bacteroidia_Bacteroidales_Prevotellaceae_Prevotella_ASV28 | Total T3 |
| Bacteroidetes_NA_NA_NA_NA_ASV182 | Total T3 |
| Bacteroidetes_Bacteroidia_Bacteroidales_Rikenellaceae_NA_ASV13 | Total T3 |
| Candidatus_Saccharibacteria_NA_NA_NA_NA_ASV179 | Total T3 |
| Candidatus_Saccharibacteria_NA_NA_NA_NA_ASV67 | Total T3 |
| Candidatus_Saccharibacteria_NA_NA_NA_NA_ASV289 | Total T3 |
| Candidatus_Saccharibacteria_NA_NA_NA_NA_ASV249 | Total T3 |
| Firmicutes_Clostridia_Clostridiales_Clostridiales_Incertae_Sedis_XIII_Mogibacterium_ASV335 | Total T3 |
| Firmicutes_Clostridia_Clostridiales_Clostridiales_Incertae_Sedis_XIII_Anaerovorax_ASV417 | Total T3 |
| Firmicutes_Erysipelotrichia_Erysipelotrichales_Erysipelotrichaceae_Faecalitalea_ASV250 | Total T3 |
| Firmicutes_Erysipelotrichia_Erysipelotrichales_Erysipelotrichaceae_Holdemania_ASV376 | Total T3 |
| Firmicutes_Clostridia_Clostridiales_NA_NA_ASV327 | Total T3 |
| Firmicutes_Clostridia_Clostridiales_Ruminococcaceae_Ruminococcus_ASV382 | Total T3 |
| Firmicutes_Clostridia_Clostridiales_Lachnospiraceae_NA_ASV16 | Total T3 |
| Spirochaetes_Spirochaetia_Spirochaetales_Spirochaetaceae_Treponema_ASV273 | Total T3 |
| Spirochaetes_Spirochaetia_Spirochaetales_Spirochaetaceae_Treponema_ASV5 | Total T3 |
| Spirochaetes_Spirochaetia_Spirochaetales_Spirochaetaceae_Treponema_ASV62 | Total T3 |
| Spirochaetes_Spirochaetia_Spirochaetales_Spirochaetaceae_Sphaerochaeta_ASV288 | Total T3 |
| Spirochaetes_Spirochaetia_Spirochaetales_Spirochaetaceae_Sphaerochaeta_ASV518 | Total T3 |
| Verrucomicrobia_Subdivision5_NA_NA_NA_ASV282 | Total T3 |
| Bacteroidetes_NA_NA_NA_NA_ASV307 | Free T4 |
| Bacteroidetes_Bacteroidia_Bacteroidales_Prevotellaceae_Prevotella_ASV223 | Free T4 |
| Bacteroidetes_Bacteroidia_Bacteroidales_Prevotellaceae_Prevotella_ASV562 | Free T4 |
| Bacteroidetes_Bacteroidia_Bacteroidales_Prevotellaceae_Prevotella_ASV9 | Free T4 |
| Bacteroidetes_Bacteroidia_Bacteroidales_Porphyromonadaceae_NA_ASV169 | Free T4 |
| Bacteroidetes_NA_NA_NA_NA_ASV187 | Free T4 |
| Bacteroidetes_Bacteroidia_Bacteroidales_Rikenellaceae_Rikenella_ASV141 | Free T4 |
| Bacteroidetes_NA_NA_NA_NA_ASV351 | Free T4 |
| Bacteroidetes_Bacteroidia_Bacteroidales_NA_NA_ASV36 | Free T4 |
| Bacteroidetes_Bacteroidia_Bacteroidales_Rikenellaceae_NA_ASV79 | Free T4 |
| Bacteroidetes_Bacteroidia_Bacteroidales_Rikenellaceae_NA_ASV372 | Free T4 |
| Elusimicrobia_Endomicrobia_Candidatus_Endomicrobium_NA_NA_ASV606 | Free T4 |
| Firmicutes_Negativicutes_Selenomonadales_Acidaminococcaceae_Phascolarctobacterium_ASV52 | Free T4 |
| Firmicutes_Clostridia_Clostridiales_NA_NA_ASV327 | Free T4 |
| Firmicutes_Clostridia_Clostridiales_Ruminococcaceae_Ruminococcus_ASV311 | Free T4 |
| Firmicutes_Clostridia_Clostridiales_Ruminococcaceae_Ruminococcus_ASV382 | Free T4 |
| Firmicutes_Clostridia_Clostridiales_Lachnospiraceae_NA_ASV58 | Free T4 |
| Firmicutes_Clostridia_Clostridiales_Lachnospiraceae_NA_ASV412 | Free T4 |
| Spirochaetes_Spirochaetia_Spirochaetales_Spirochaetaceae_Sphaerochaeta_ASV518 | Free T4 |
| Verrucomicrobia_Subdivision5_NA_NA_NA_ASV540 | Free T4 |

**Table S8. Non-linear relationships from Threshold Indicator Taxa Analysis (TITAN) between bacterial ASV relative abundance and reproductive and metabolic hormones in Asian elephants**

| Taxonomic Assignment | Hormone |
| --- | --- |
| Bacteroidetes_NA_NA_NA_NA_ASV61 | LH |
| Bacteroidetes_Bacteroidia_Bacteroidales_Prevotellaceae_Paraprevotella_ASV11 | LH |
| Bacteroidetes_Bacteroidia_Bacteroidales_Prevotellaceae_Prevotella_ASV28 | LH |
| Bacteroidetes_NA_NA_NA_NA_ASV243 | LH |
| Bacteroidetes_Bacteroidia_Bacteroidales_Porphyromonadaceae_Falsiporphyromonas_ASV624 | LH |
| Bacteroidetes_Bacteroidia_Bacteroidales_NA_NA_ASV145 | LH |
| Bacteroidetes_NA_NA_NA_NA_ASV77 | LH |
| Bacteroidetes_NA_NA_NA_NA_ASV89 | LH |
| Bacteroidetes_Sphingobacteriia_Sphingobacteriales_Sphingobacteriaceae_NA_ASV30 | LH |
| Firmicutes_Clostridia_Clostridiales_Peptococcaceae_1_Dehalobacter_ASV72 | LH |
| Firmicutes_Clostridia_Clostridiales_Peptococcaceae_1_Dehalobacter_ASV50 | LH |
| Firmicutes_Clostridia_Clostridiales_Ruminococcaceae_Ruminococcus_ASV124 | LH |
| Firmicutes_Clostridia_Clostridiales_Lachnospiraceae_NA_ASV224 | LH |
| Spirochaetes_Spirochaetia_Spirochaetales_Spirochaetaceae_Treponema_ASV144 | LH |
| Spirochaetes_Spirochaetia_Spirochaetales_Spirochaetaceae_Treponema_ASV219 | LH |
| Verrucomicrobia_Subdivision5_NA_NA_NA_ASV310 | LH |
| Verrucomicrobia_Subdivision5_NA_NA_NA_ASV189 | LH |
| Armatimonadetes_Armatimonadetes_gp2_NA_NA_NA_ASV143 | FGM |
| Bacteroidetes_Bacteroidia_Bacteroidales_Bacteroidales_incertae_sedis_Phocaeicola_ASV94 | FGM |
| Bacteroidetes_Bacteroidia_Bacteroidales_NA_NA_ASV172 | FGM |
| Bacteroidetes_Bacteroidia_Bacteroidales_NA_NA_ASV128 | FGM |
| Spirochaetes_Spirochaetia_Spirochaetales_Spirochaetaceae_Treponema_ASV166 | FGM |
| Spirochaetes_Spirochaetia_Spirochaetales_Spirochaetaceae_Treponema_ASV112 | FGM |
| Bacteroidetes_NA_NA_NA_NA_ASV61 | Total T3 |
| Bacteroidetes_NA_NA_NA_NA_ASV88 | Total T3 |
| Bacteroidetes_Bacteroidia_Bacteroidales_Bacteroidales_incertae_sedis_Phocaeicola_ASV85 | Total T3 |
| Bacteroidetes_NA_NA_NA_NA_ASV63 | Total T3 |
| Bacteroidetes_Bacteroidia_Bacteroidales_Porphyromonadaceae_NA_ASV385 | Total T3 |
| Bacteroidetes_Bacteroidia_Bacteroidales_Rikenellaceae_Mucinivorans_ASV236 | Total T3 |
| Bacteroidetes_Bacteroidia_Bacteroidales_Rikenellaceae_NA_ASV13 | Total T3 |
| Bacteroidetes_Sphingobacteriia_Sphingobacteriales_Sphingobacteriaceae_NA_ASV30 | Total T3 |
| Firmicutes_Clostridia_Clostridiales_Peptococcaceae_1_Dehalobacter_ASV72 | Total T3 |
| Firmicutes_Clostridia_Clostridiales_NA_NA_ASV253 | Total T3 |
| Firmicutes_Clostridia_Clostridiales_Ruminococcaceae_Ruminococcus_ASV124 | Total T3 |
| Lentisphaerae_Lentisphaeria_Victivallales_Victivallaceae_Victivallis_ASV521 | Total T3 |
| Spirochaetes_Spirochaetia_Spirochaetales_Spirochaetaceae_Treponema_ASV1 | Total T3 |
| Spirochaetes_Spirochaetia_Spirochaetales_Spirochaetaceae_Treponema_ASV480 | Total T3 |
| Spirochaetes_Spirochaetia_Spirochaetales_Spirochaetaceae_Treponema_ASV69 | Total T3 |
| Spirochaetes_Spirochaetia_Spirochaetales_Spirochaetaceae_Treponema_ASV135 | Total T3 |
| Synergistetes_Synergistia_Synergistales_Synergistaceae_Cloacibacillus_ASV113 | Total T3 |
| Verrucomicrobia_Subdivision5_NA_NA_NA_ASV47 | Total T3 |
| Bacteroidetes_Bacteroidia_Bacteroidales_Porphyromonadaceae_NA_ASV495 | Free T4 |
