# Supplementary material for: Clinical health issues, reproductive hormones, and metabolic hormones associated with gut microbiome structure in African and Asian elephants
Source: Anim Microbiome. 2021 Dec 20;3:85. doi: 10.1186/s42523-021-00146-9 (PMC8686393; doi:10.1186/s42523-021-00146-9)
Supplement: Supplementary file 3 — Additional file 3. Linear and non-linear correlations of bacterial taxa relative abundance and reproductive and metabolic hormones in captive African and Asian elephants. [file 42523_2021_146_MOESM3_ESM.docx]

**Additional file 3:** Linear and non-linear correlations of bacterial taxa relative abundance and reproductive and metabolic hormones in captive African and Asian elephants.

**This file includes:**

*LINEAR CORRELATIONS*

Figure S6. Bacterial taxa relative abundance correlated with PRL in African elephants

Figure S7. Bacterial taxa relative abundance correlated with LH in African elephants

Figure S8. Bacterial taxa relative abundance correlated with FSH in African elephants

Figure S9. Bacterial taxa relative abundance correlated with FGM in African elephants

Figure S10. Bacterial taxa relative abundance correlated with age in African elephants

Figure S11. Bacterial taxa relative abundance correlated with BCS in African elephants

Figure S12. Bacterial taxa relative abundance correlated with lameness/stiffness in African elephants

Figure S13. Bacterial taxa relative abundance correlated with progestagen in Asian elephants

Figure S14. Bacterial taxa relative abundance correlated with PRL in Asian elephants

Figure S15. Bacterial taxa relative abundance correlated with LH in Asian elephants

Figure S16. Bacterial taxa relative abundance correlated with FSH in Asian elephants

Figure S17. Bacterial taxa relative abundance correlated with FGM in Asian elephants

Figure S18. Bacterial taxa relative abundance correlated with total T3 in Asian elephants

Figure S19. Bacterial taxa relative abundance correlated with free T4 in Asian elephants

Figure S20. Bacterial taxa relative abundance correlated with total T4 in Asian elephants

Figure S21. Bacterial taxa relative abundance correlated with TSH in Asian elephants

Figure S22. Bacterial taxa relative abundance correlated with BCS in Asian elephants

Figure S23. Bacterial taxa relative abundance correlated with recent GI issues in Asian elephants

Figure S24. Bacterial taxa relative abundance correlated with recent lameness/stiffness in Asian elephants

Figure S25. Bacterial taxa relative abundance correlated with recent antibiotics & NSAID use in Asian elephants

Figure S26. Bacterial taxa relative abundance correlated with age in Asian elephants

*THRESHOLD INDICATOR TAXA ANALYSIS (TITAN)*

Figure S27. Non-linear relationships between bacterial ASV relative abundance and progestagen in African elephants

Figure S28. Non-linear relationships between bacterial ASV relative abundance and free T4 in African elephants

Figure S29. Non-linear relationships between bacterial ASV relative abundance and total T3 in African elephants

Figure S30. Non-linear relationships between bacterial ASV relative abundance and total TSH in African elephants

Figure S31. Non-linear relationships between bacterial ASV relative abundance and LH in Asian elephants

Figure S32. Non-linear relationships between bacterial ASV relative abundance and FGM in Asian elephants

Figure S33. Non-linear relationships between bacterial ASV relative abundance and total T3 in Asian elephants

Figure S34. Non-linear relationships between bacterial ASV relative abundance and free T4 in Asian elephants


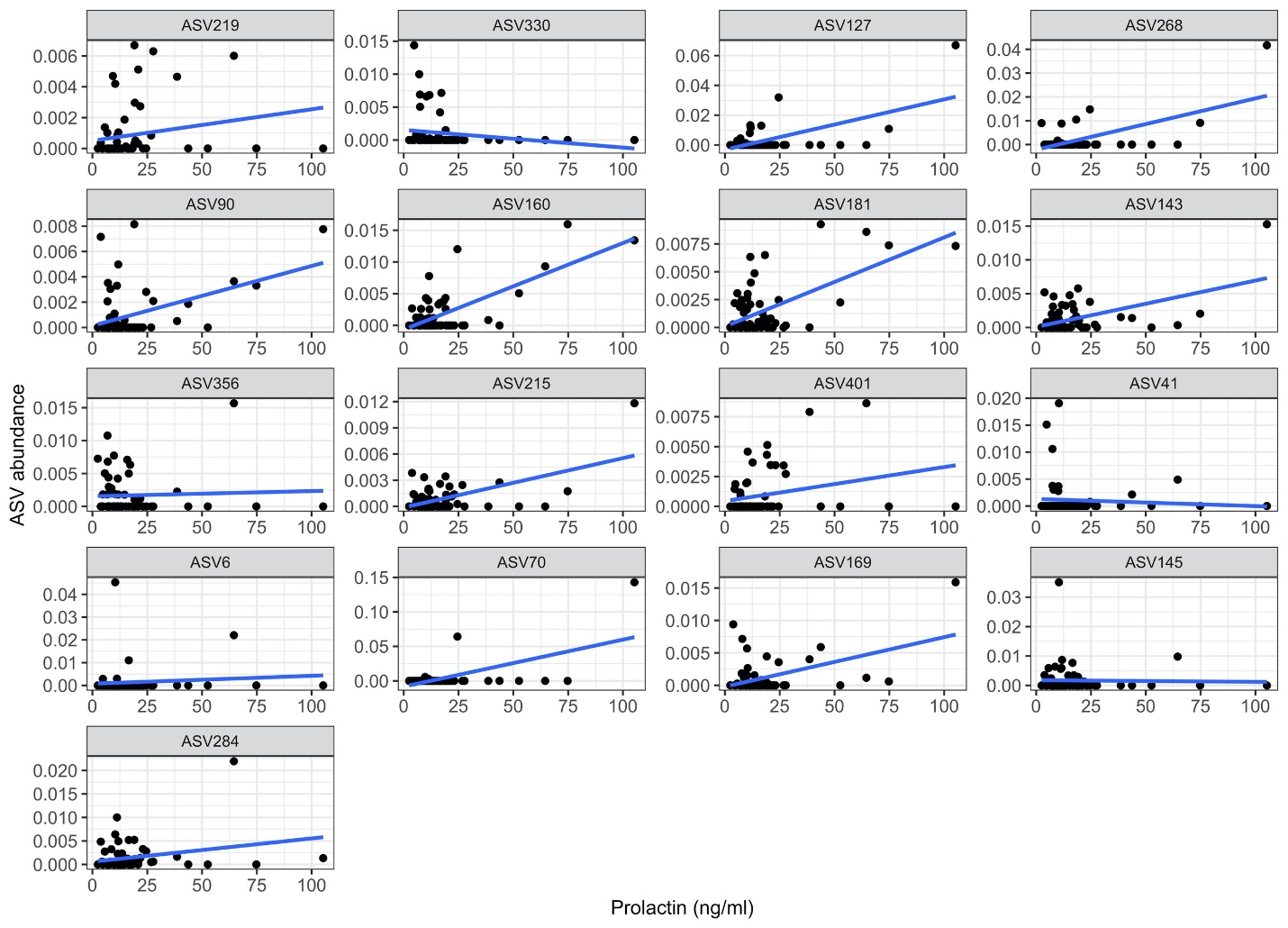


**Figure S6. Bacterial taxa relative abundance correlated with PRL in African elephants**


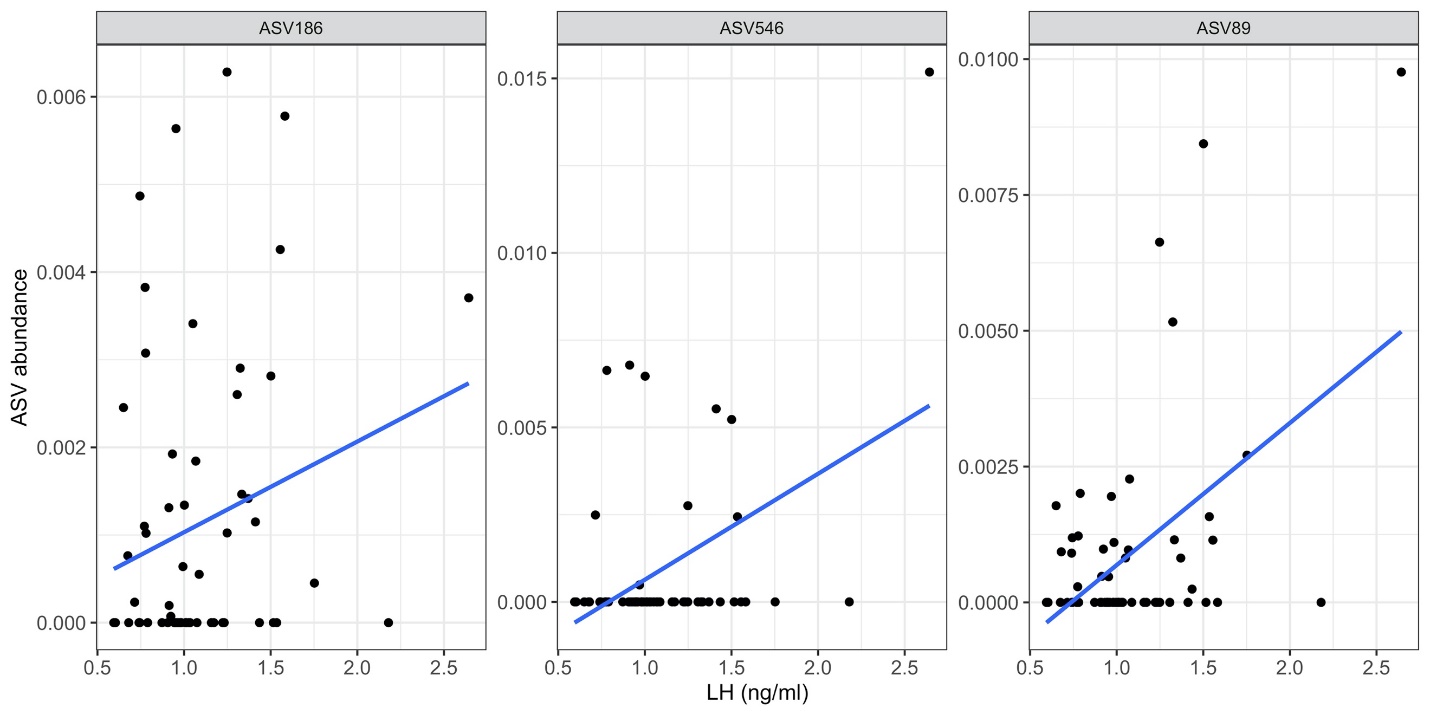


**Figure S7. Bacterial taxa relative abundance correlated with LH in African elephants**

**
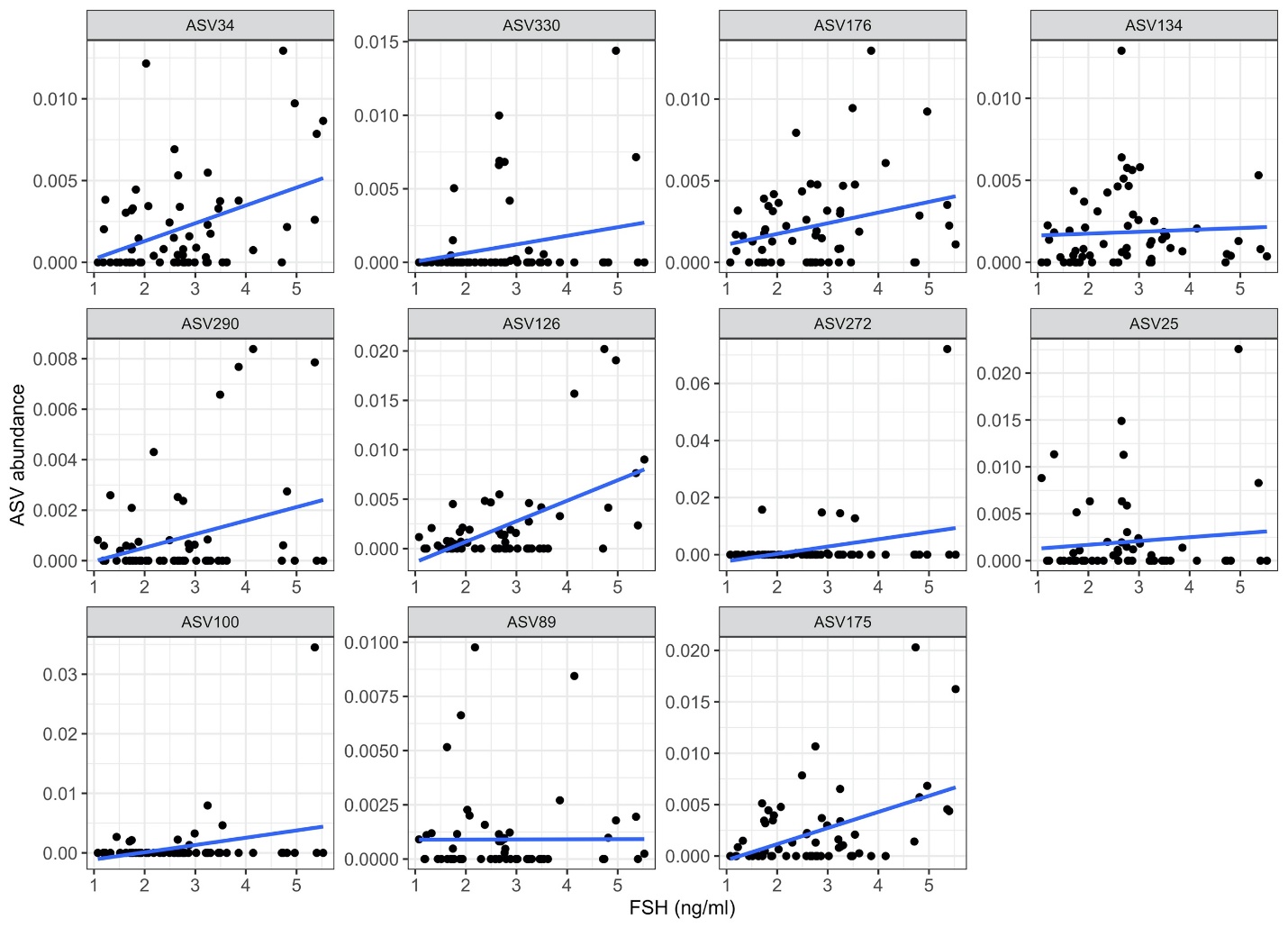
**

**Figure S8. Bacterial taxa relative abundance correlated with FSH in African elephants**


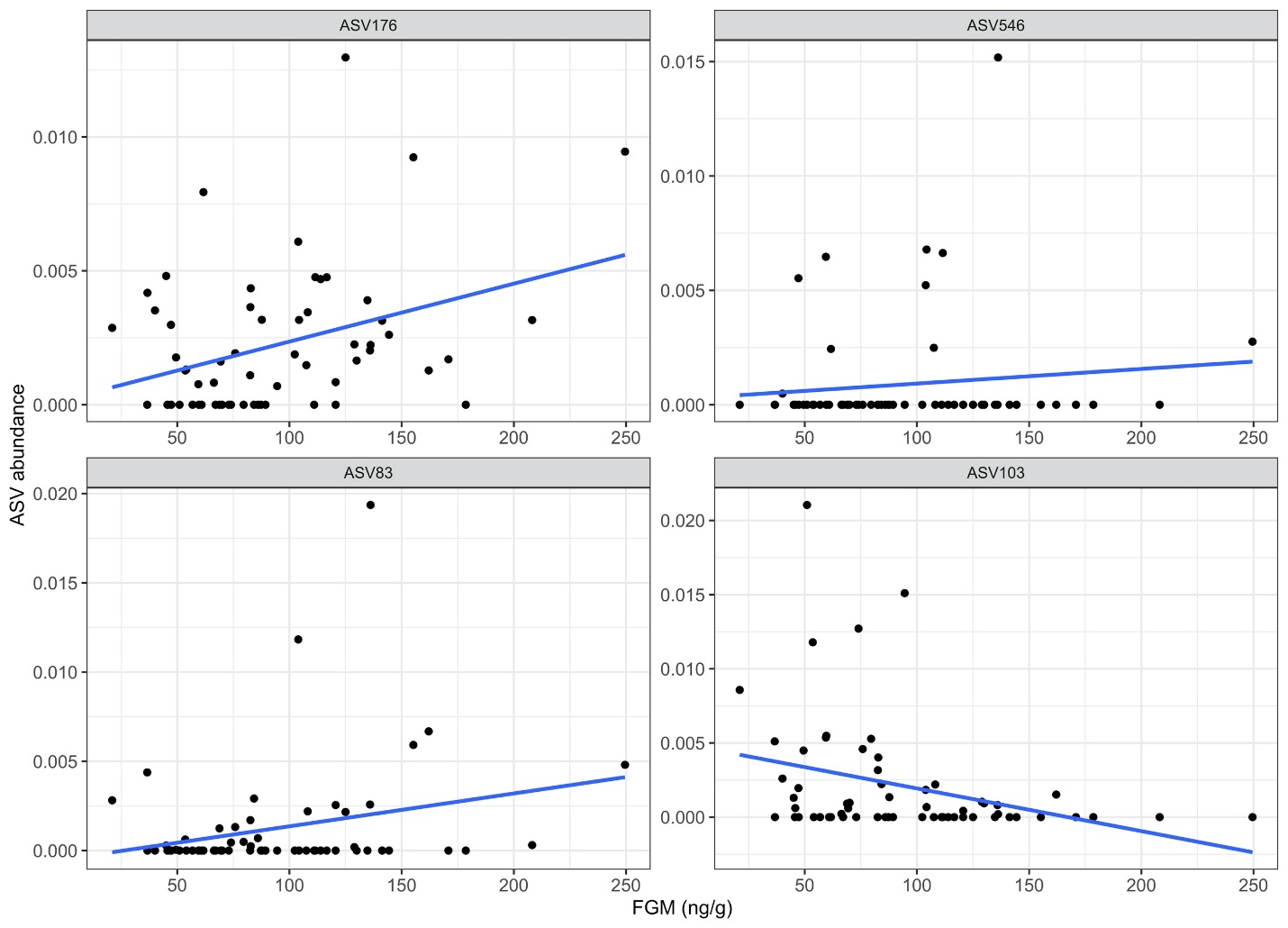


**Figure S9. Bacterial taxa relative abundance correlated with FGM in African elephants**


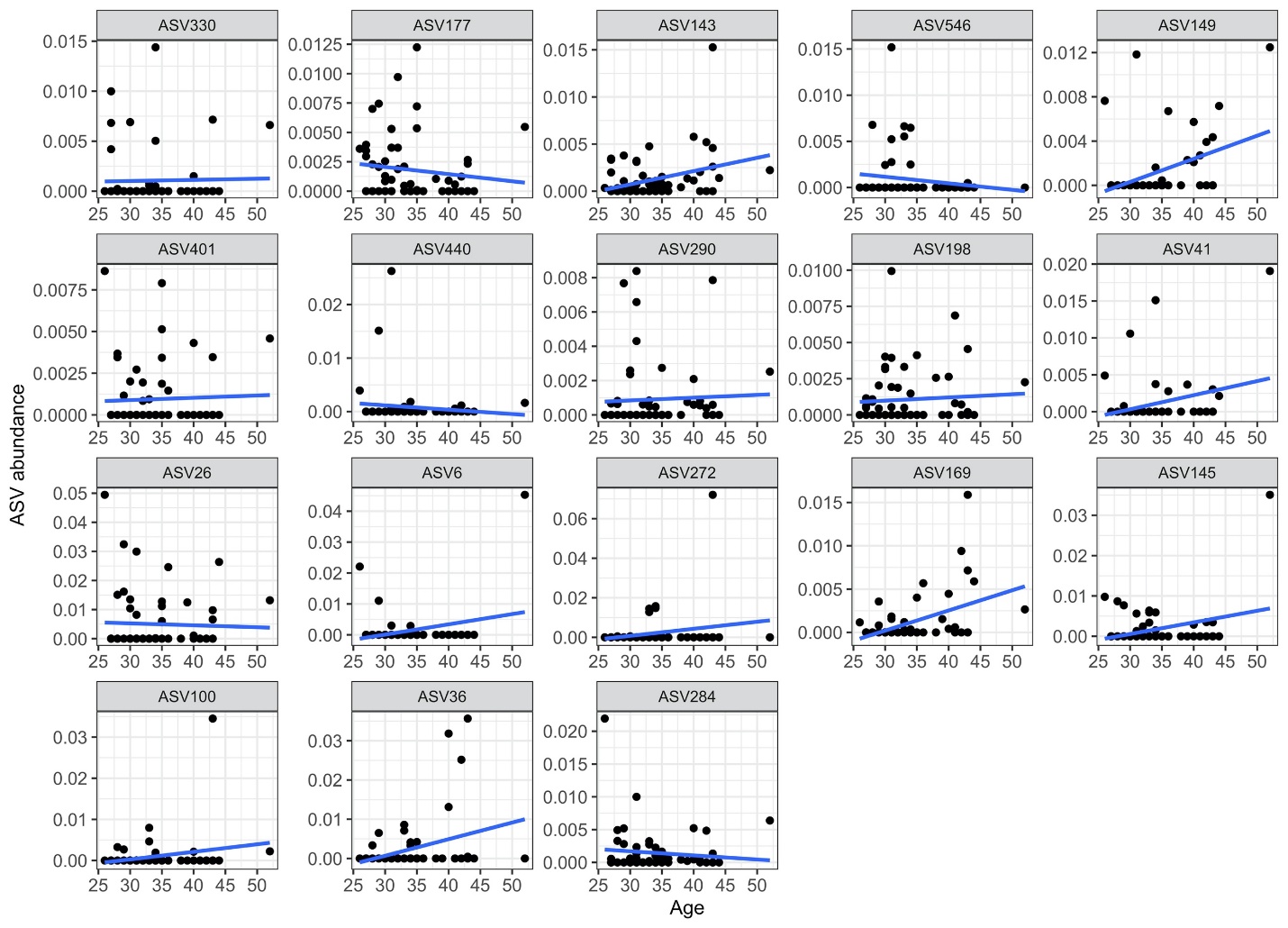


**Figure S10. Bacterial taxa relative abundance correlated with age in African elephants**

**
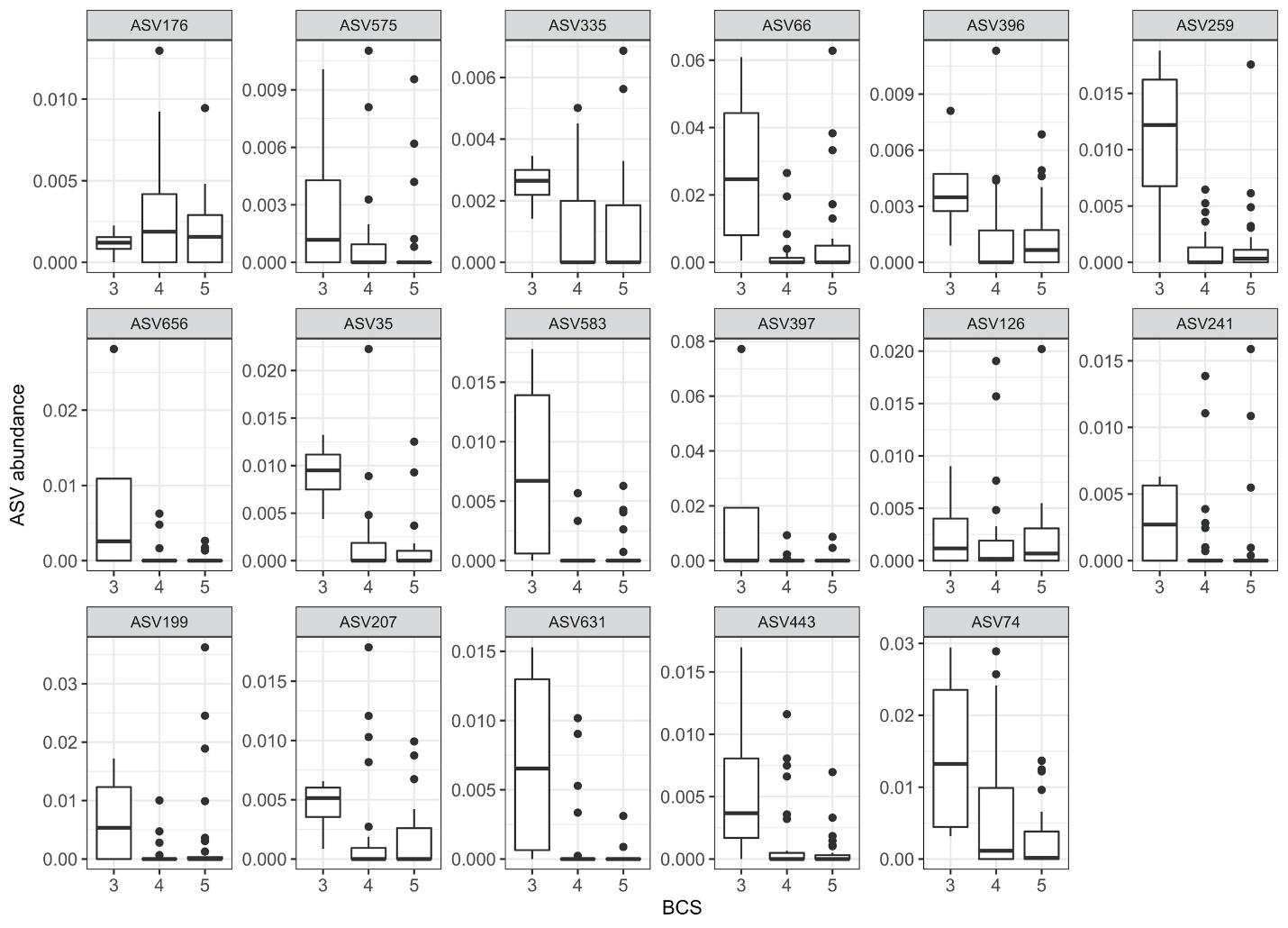
**

**Figure S11. Bacterial taxa relative abundance correlated with BCS in African elephants**

**
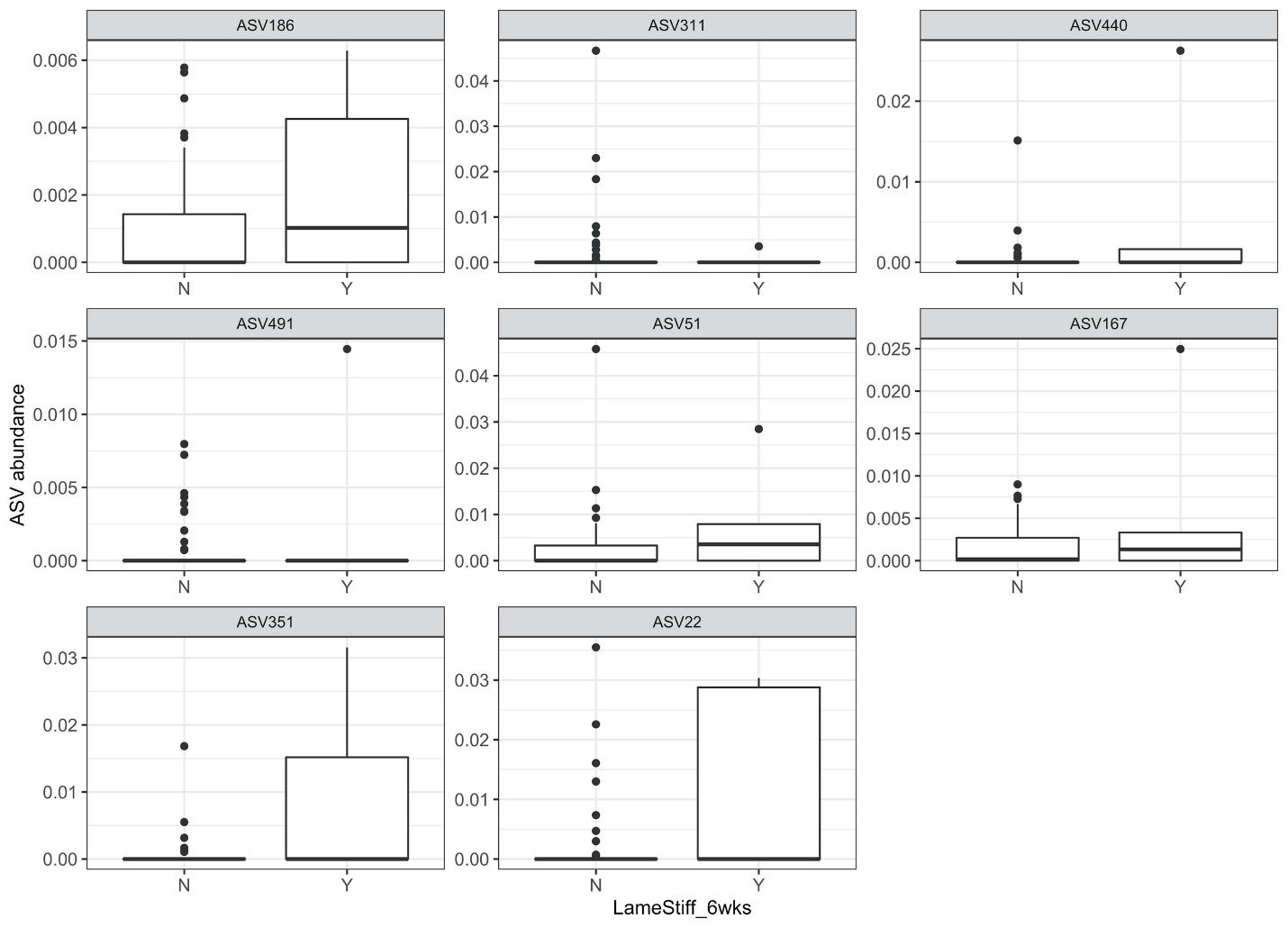
**

**Figure S12. Bacterial taxa relative abundance correlated with lameness/stiffness in African elephants**


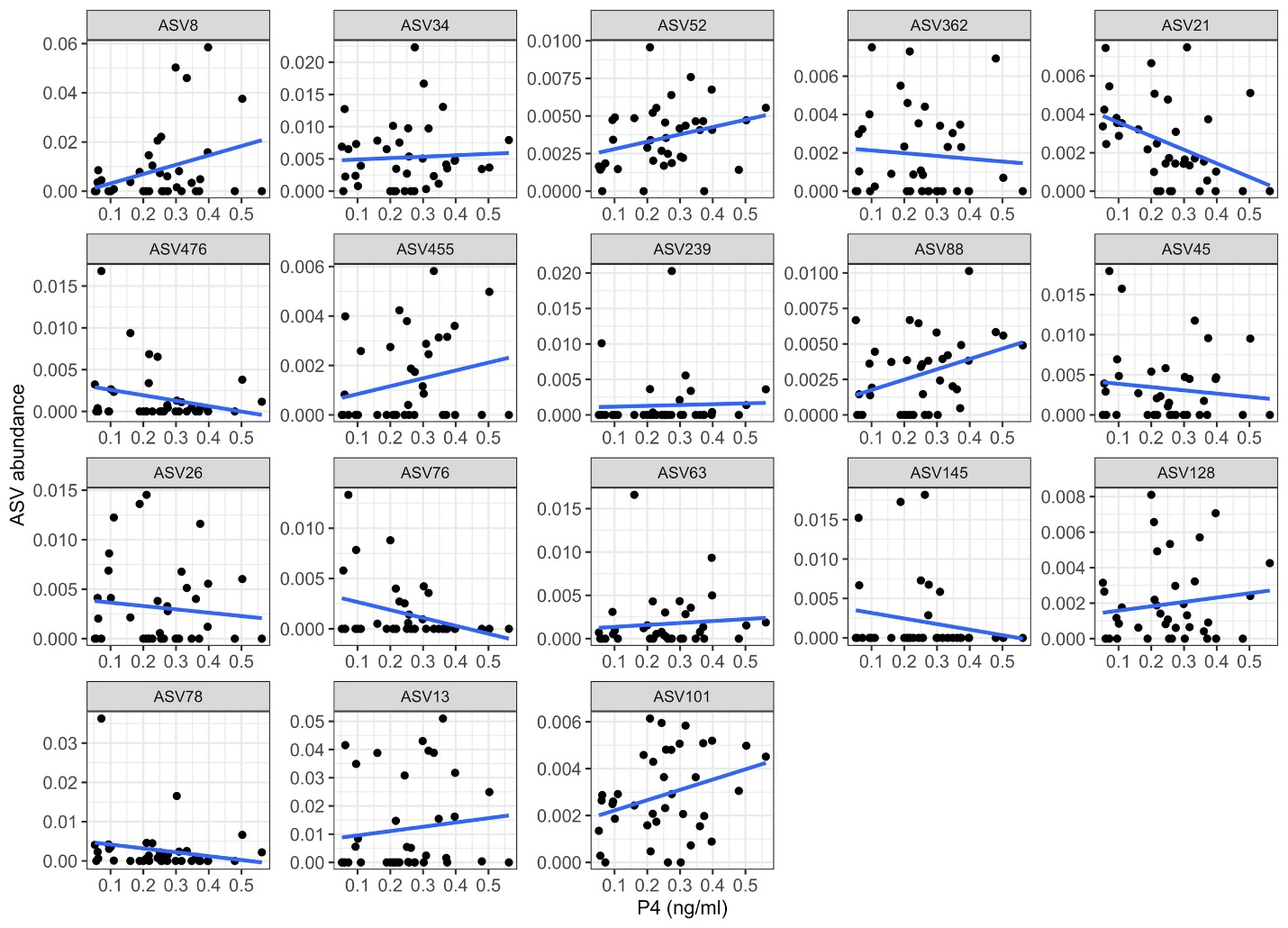


**Figure S13. Bacterial taxa relative abundance correlated with progestagen in Asian elephants**


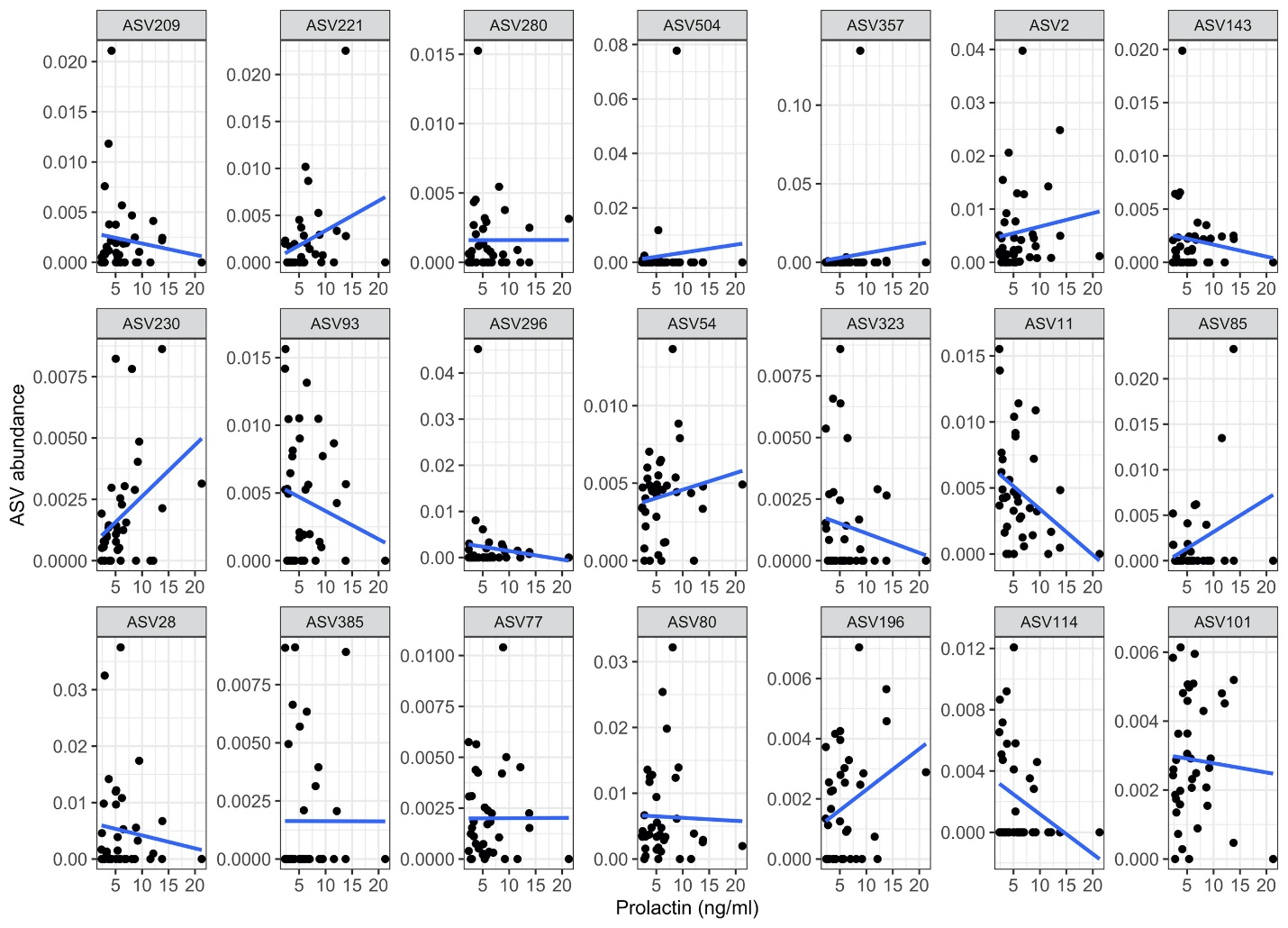


**Figure S14. Bacterial taxa relative abundance correlated with prolactin in Asian elephants**


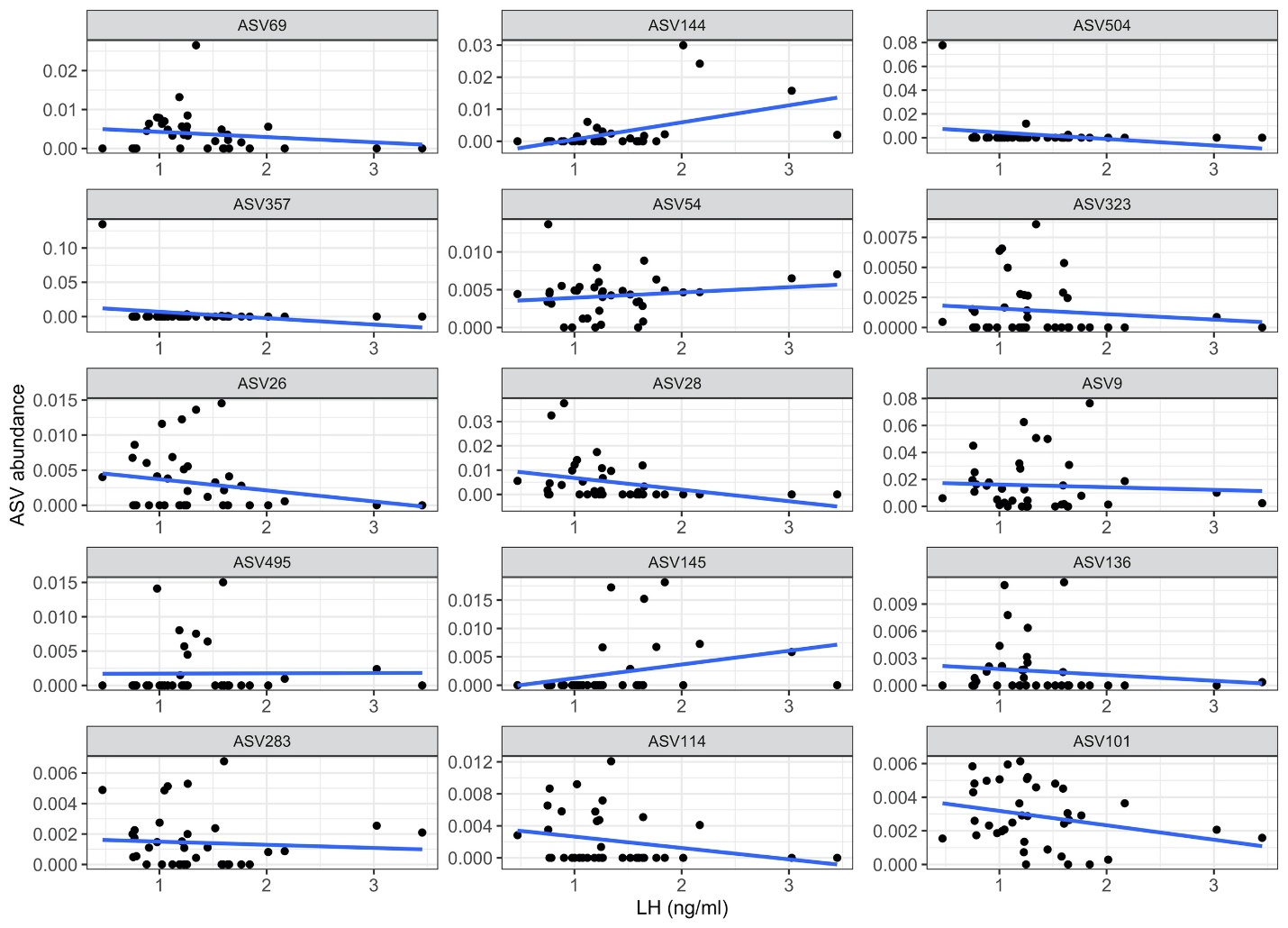


**Figure S15. Bacterial taxa relative abundance correlated with LH in Asian elephants**


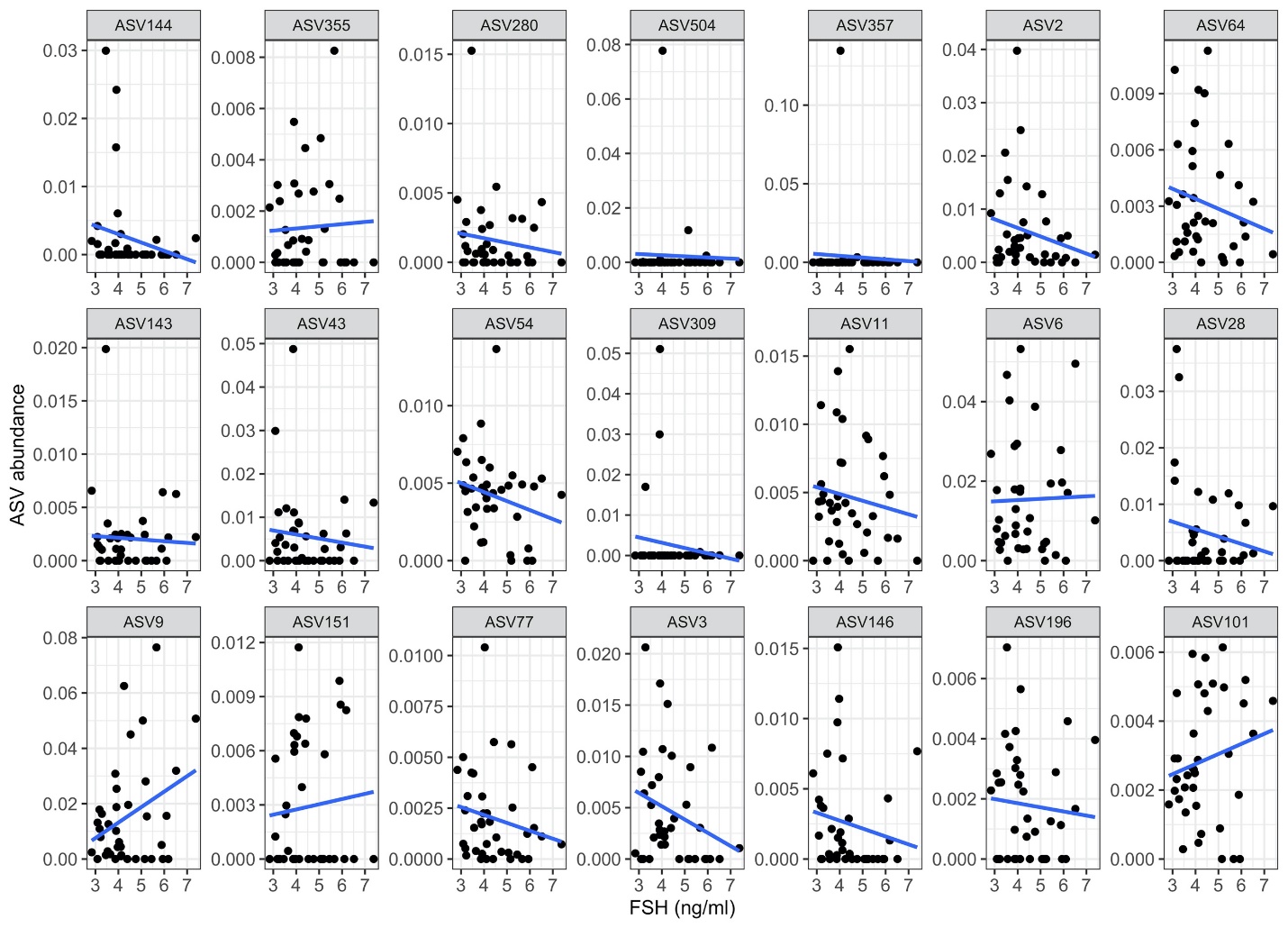


**Figure S16. Bacterial taxa relative abundance correlated with FSH in Asian elephants**


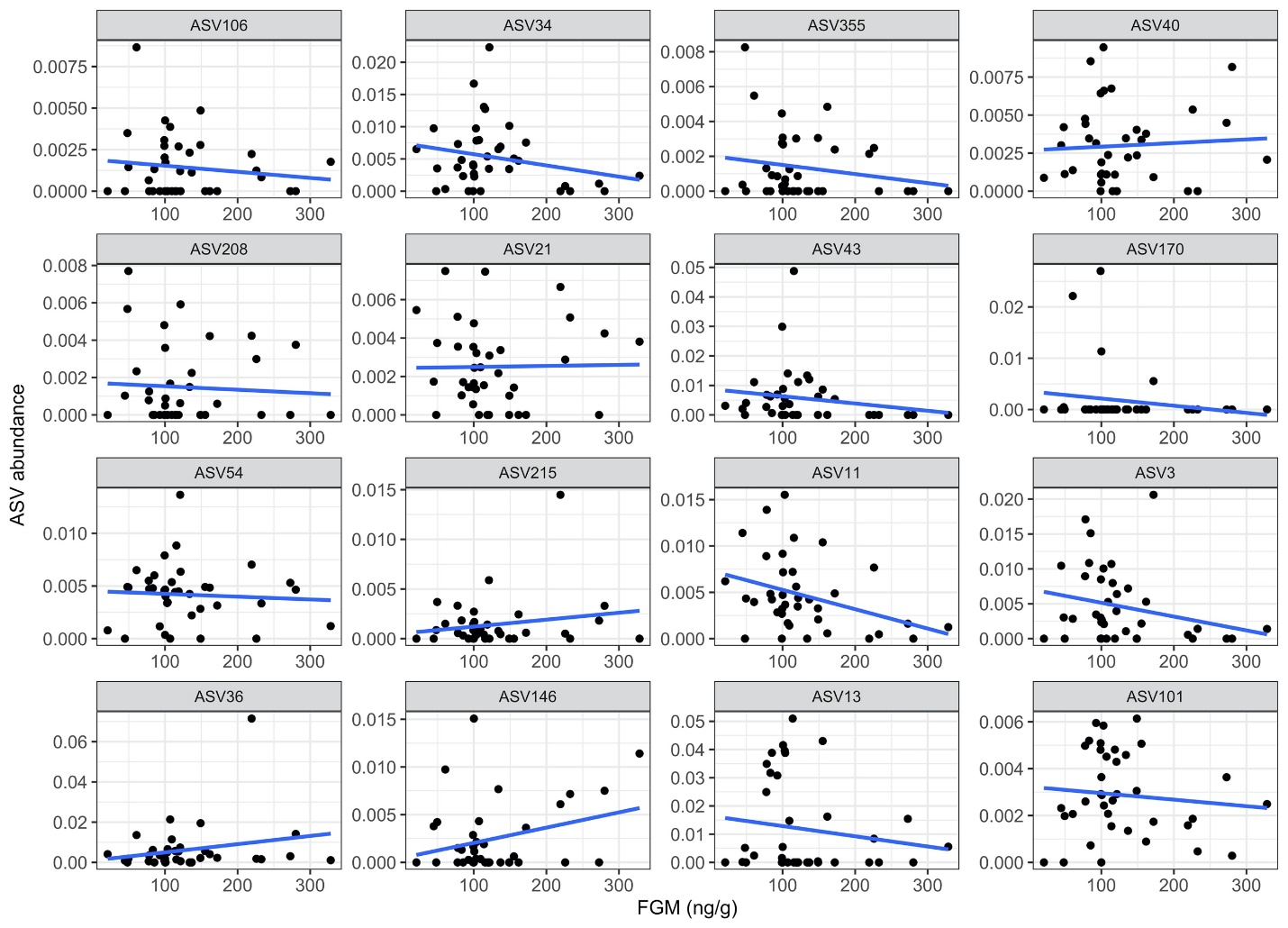


**Figure S17. Bacterial taxa relative abundance correlated with FGM in Asian elephants**

**
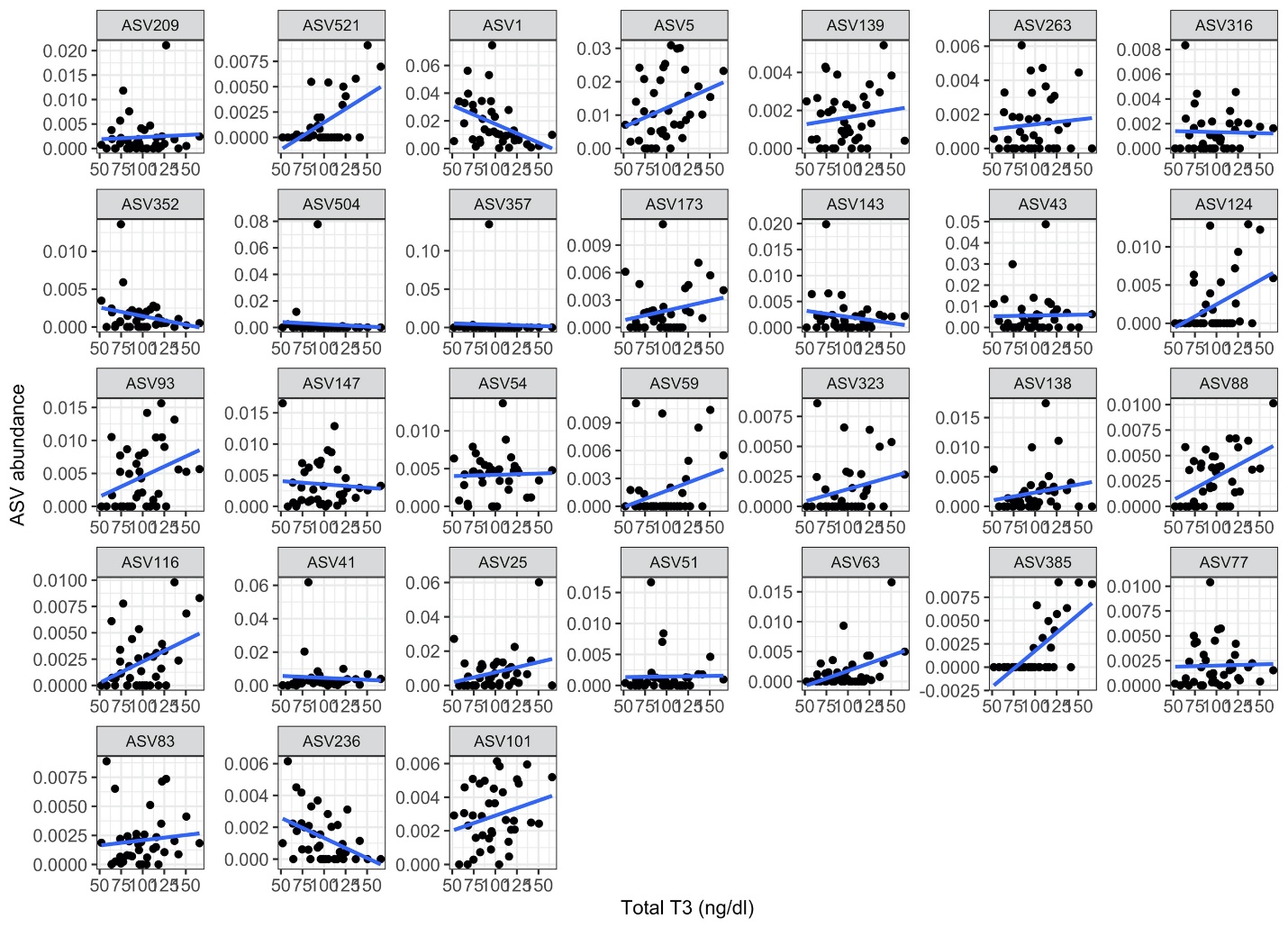
**

**Figure S18. Bacterial taxa relative abundance correlated with total T3 in Asian elephants**

**
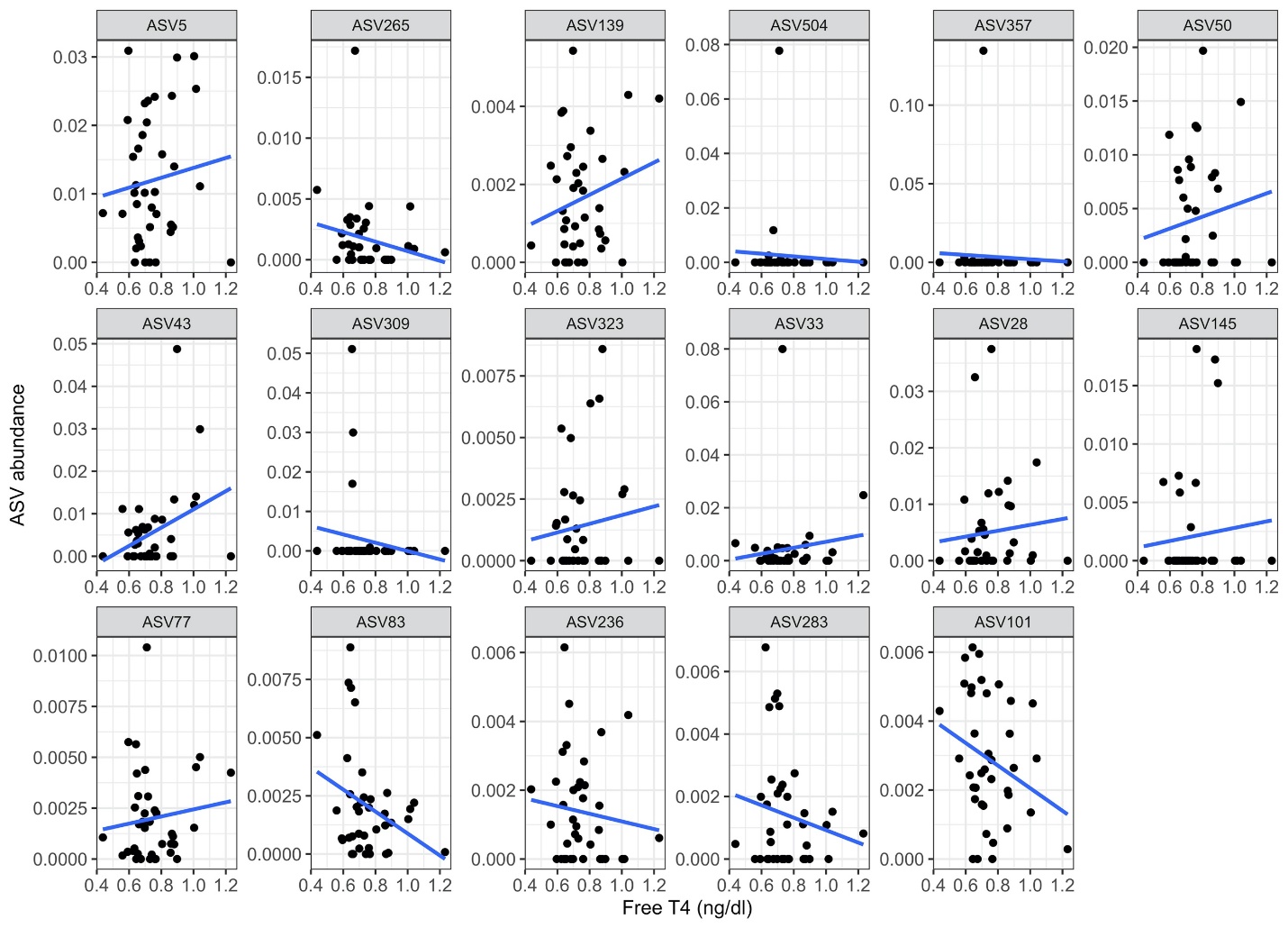
**

**Figure S19. Bacterial taxa relative abundance correlated with free T4 in Asian elephants**

**
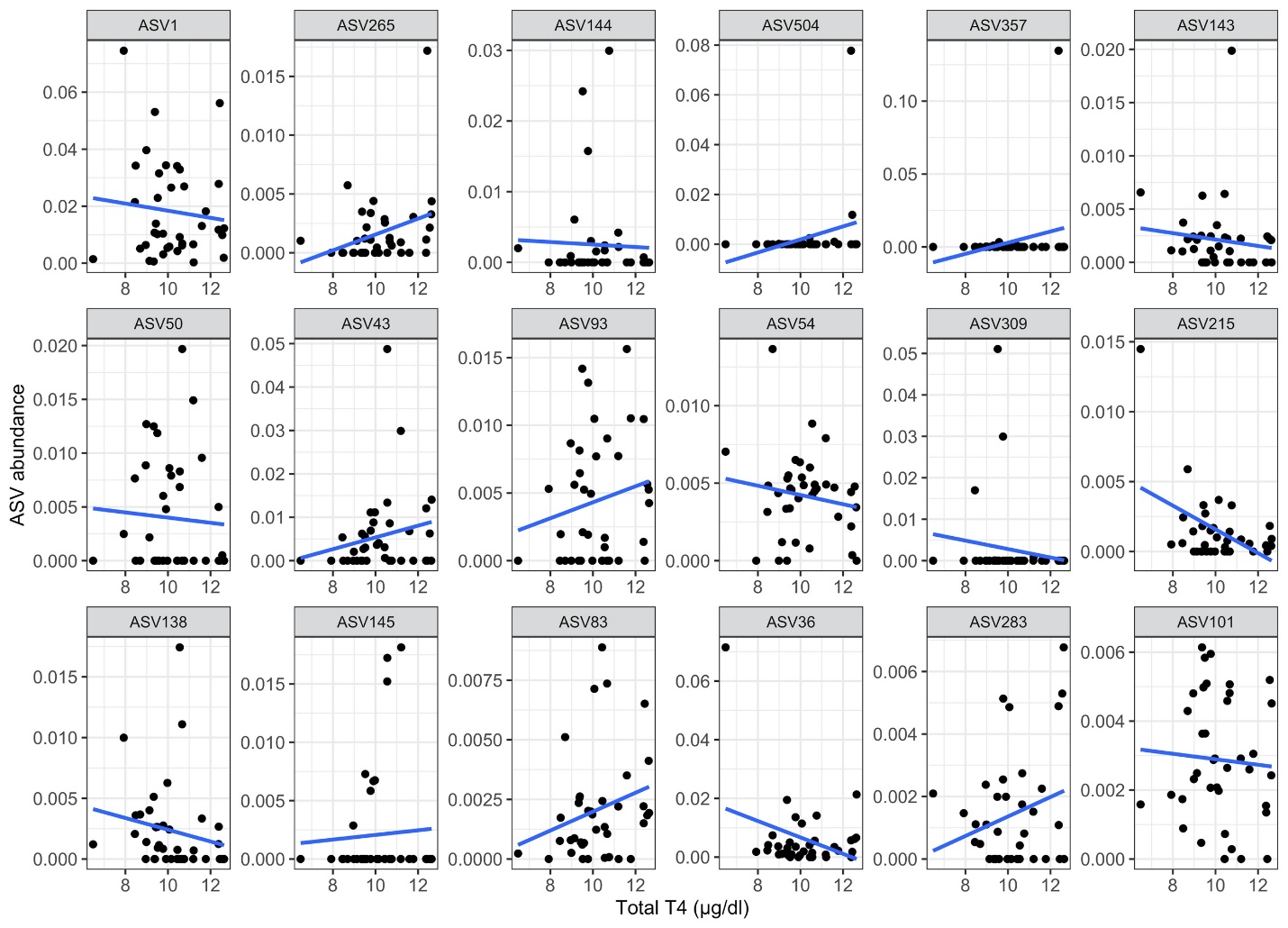
**

**Figure S20. Bacterial taxa relative abundance correlated with total T4 in Asian elephants**

**
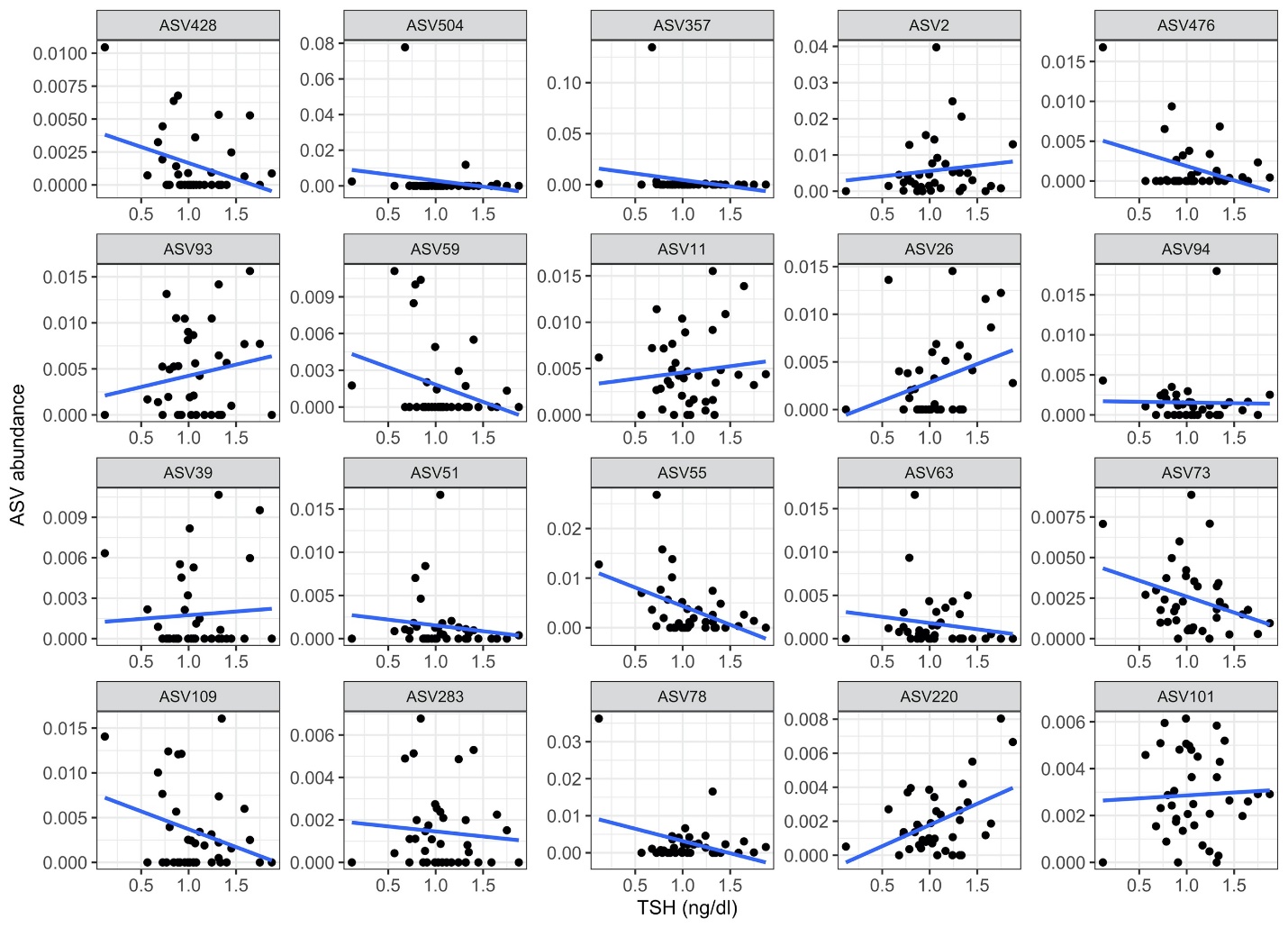
**

**Figure S21. Bacterial taxa relative abundance correlated with TSH in Asian elephants**

**
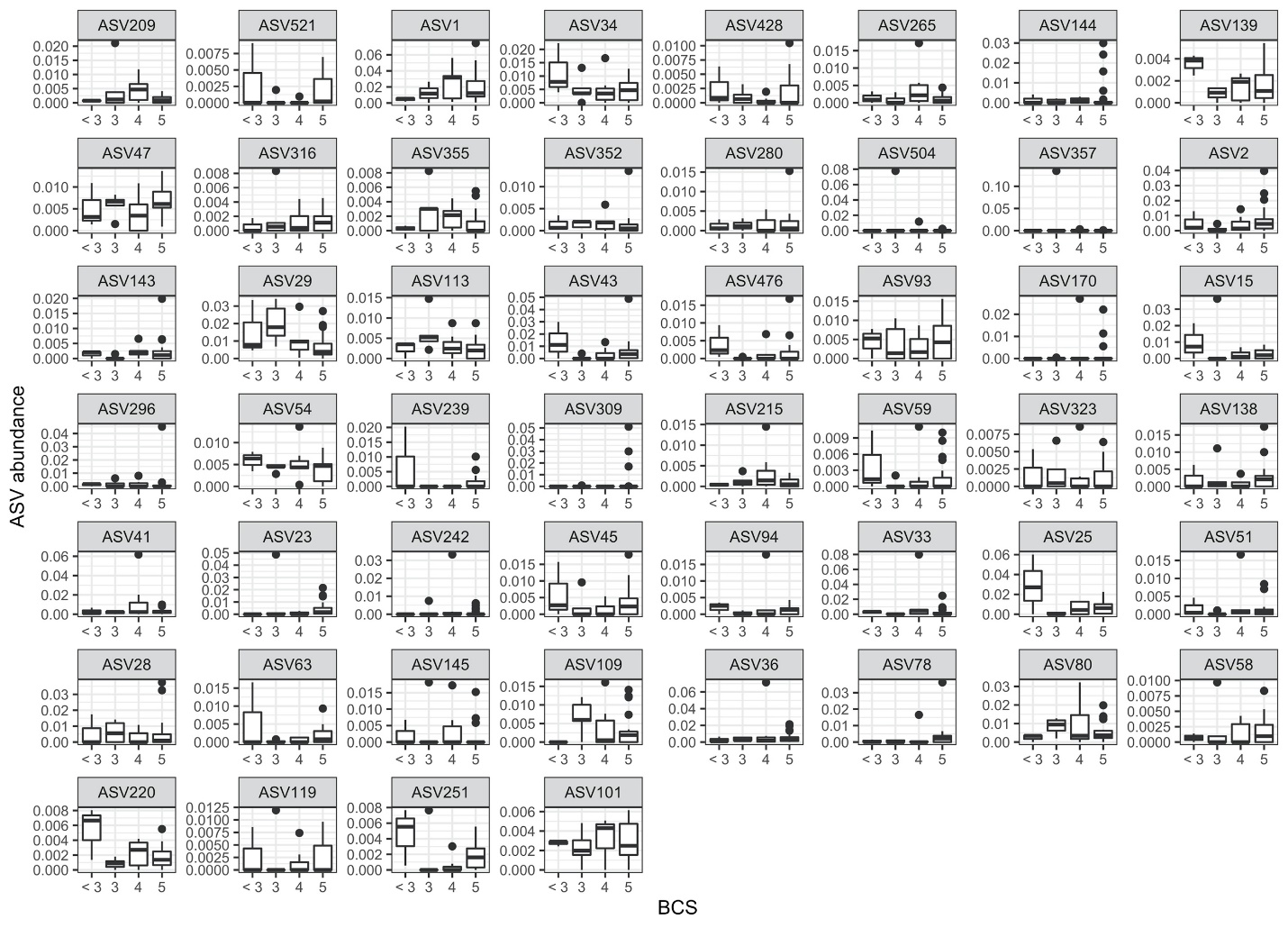
**

**Figure S22. Bacterial taxa relative abundance correlated with BCS in Asian elephants**

**
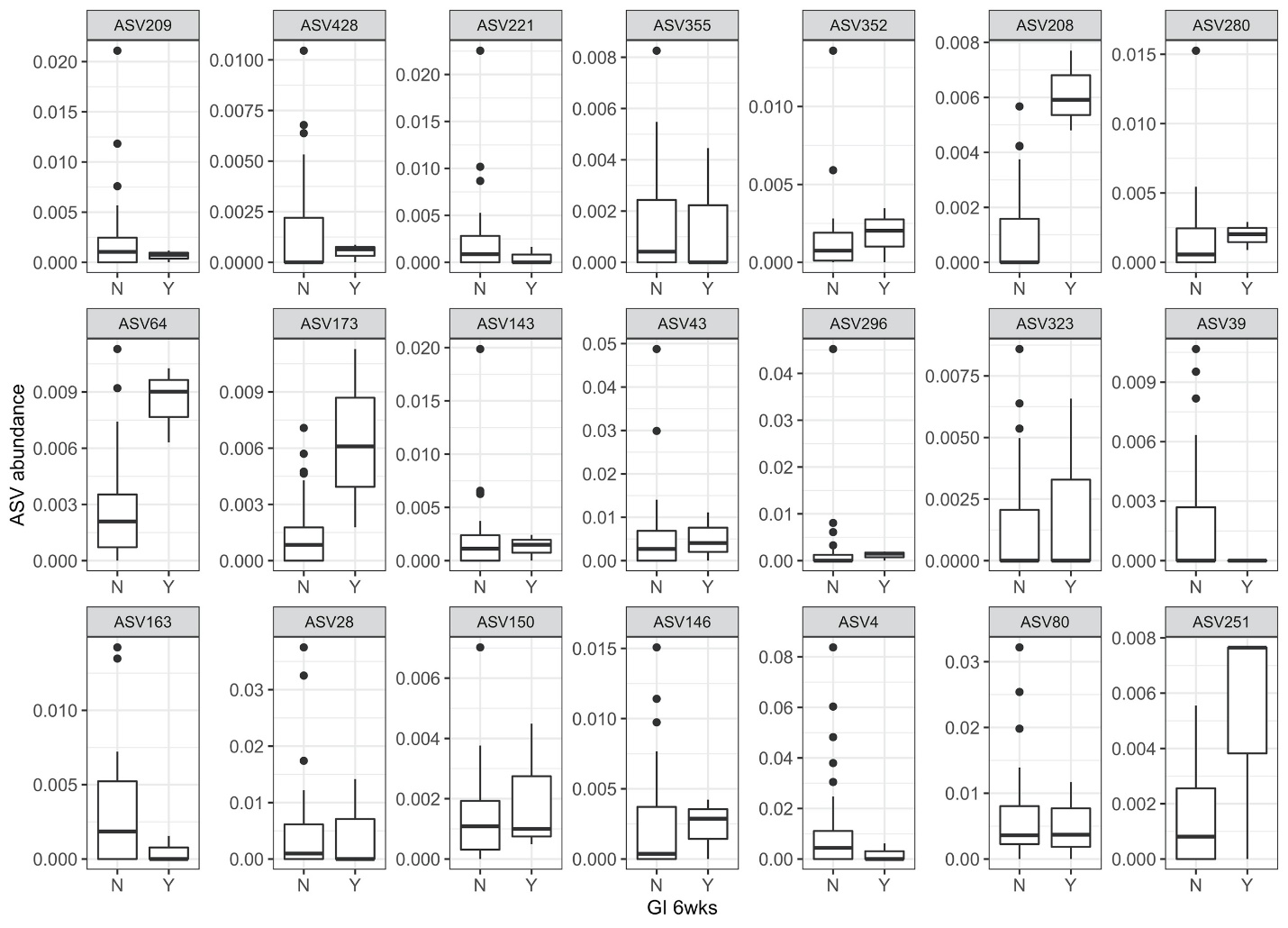
**

**Figure S23. Bacterial taxa relative abundance correlated with recent GI issues in Asian elephants**

**
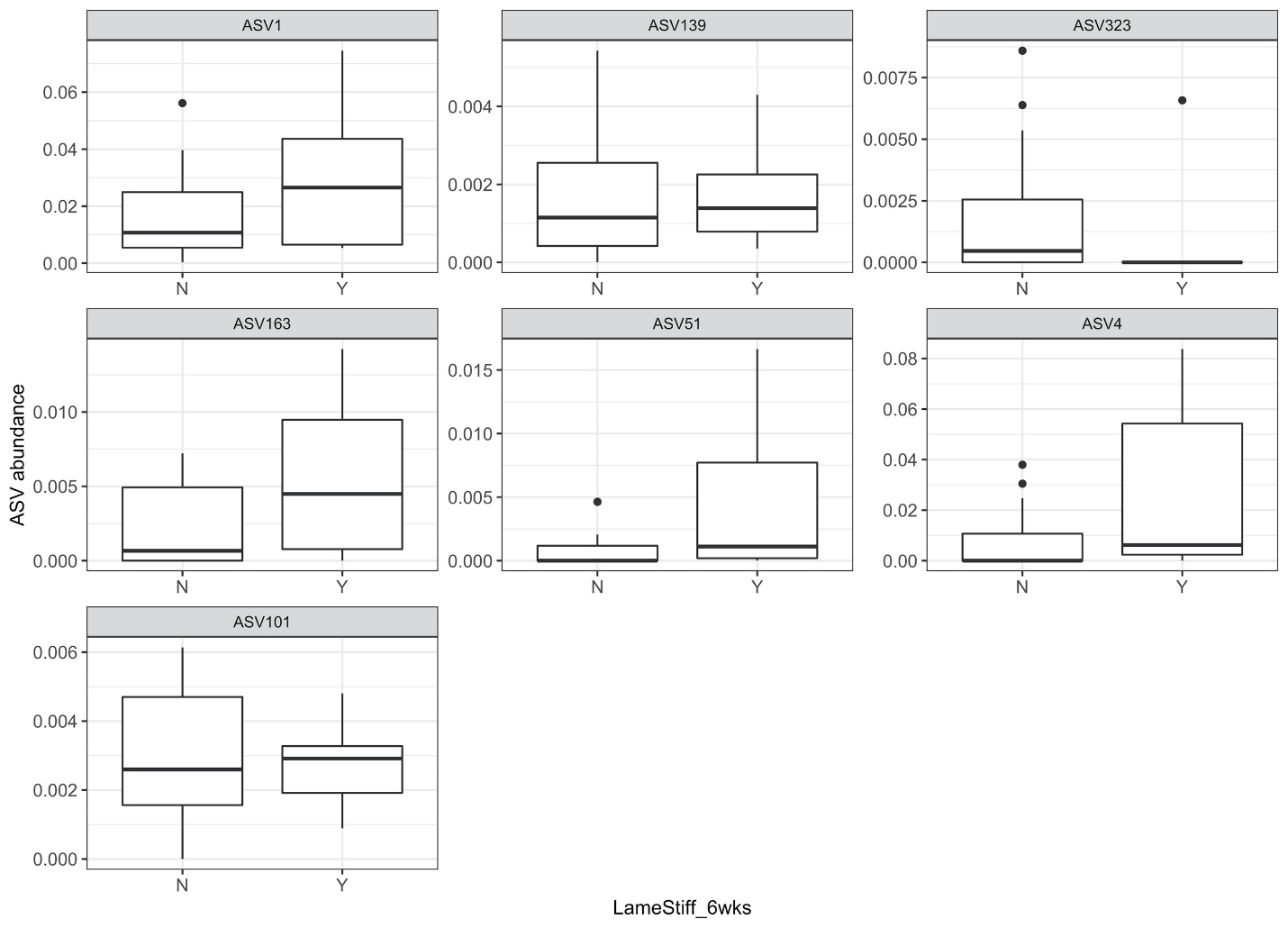
**

**Figure S24. Bacterial taxa relative abundance correlated with recent lameness/stiffness in Asian elephants**

**
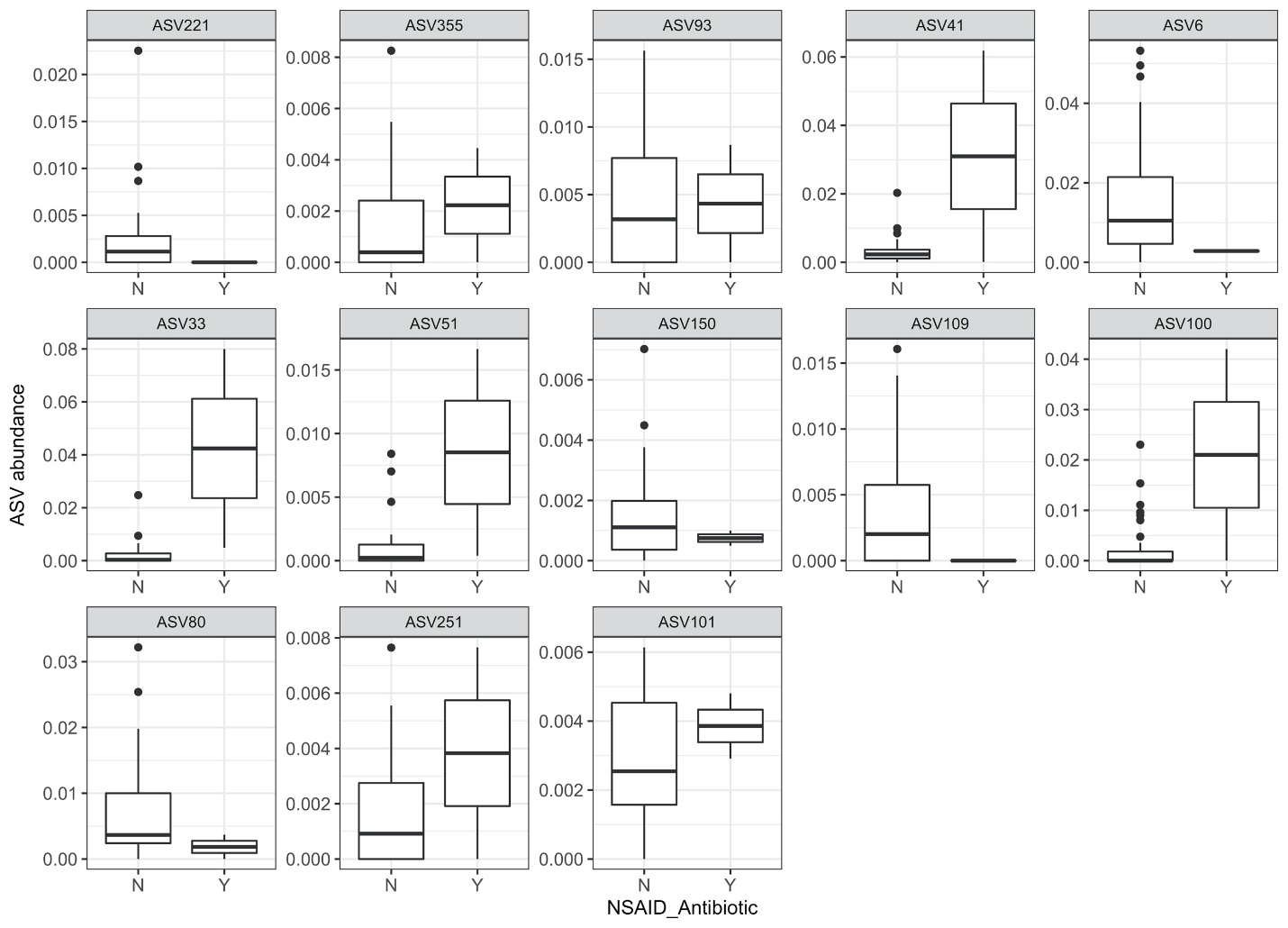
**

**Figure S25. Bacterial taxa relative abundance correlated with recent antibiotics & NSAID use in Asian elephants**

**
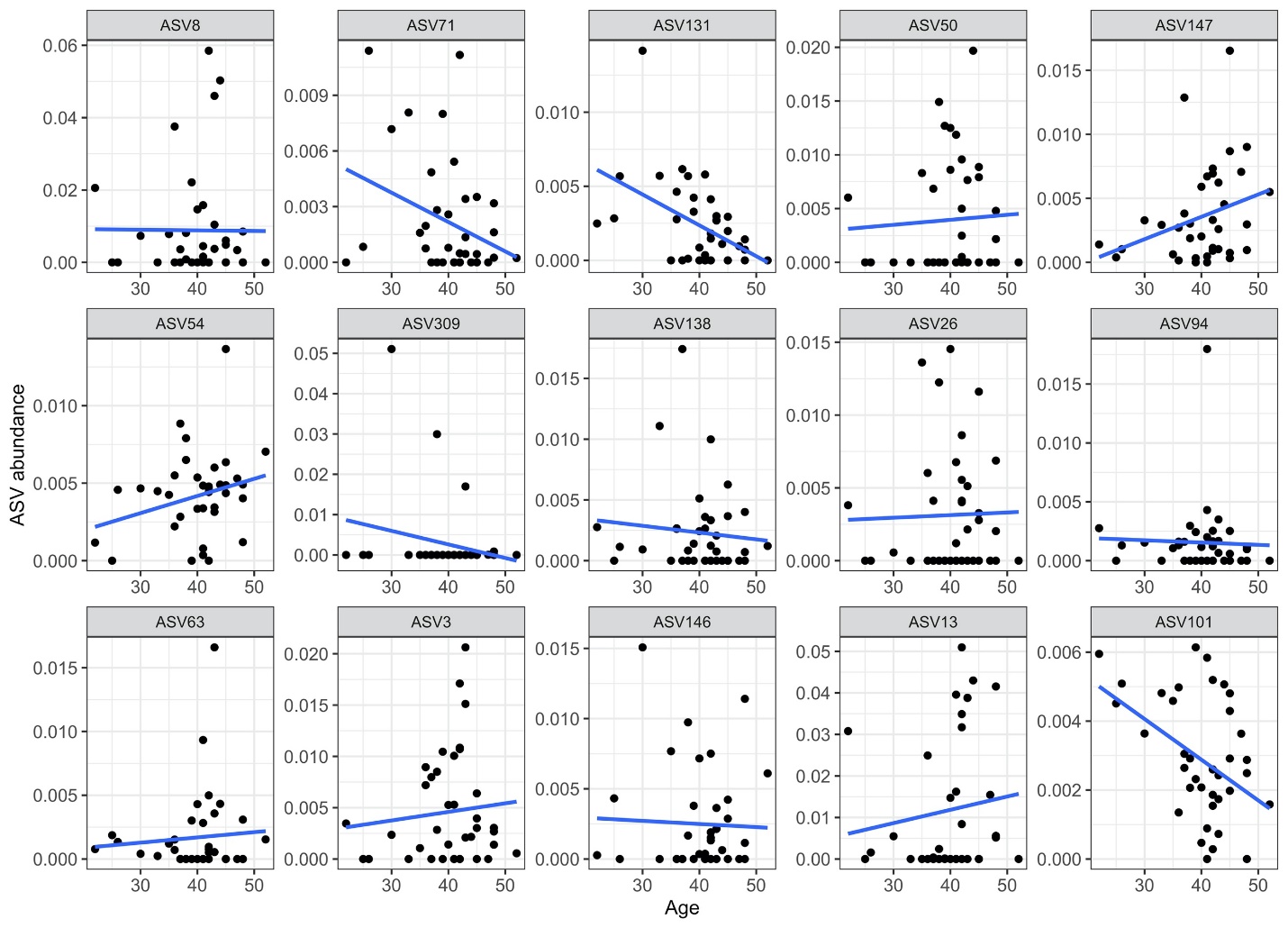
**

**Figure S26. Bacterial taxa relative abundance correlated with age in Asian elephants**


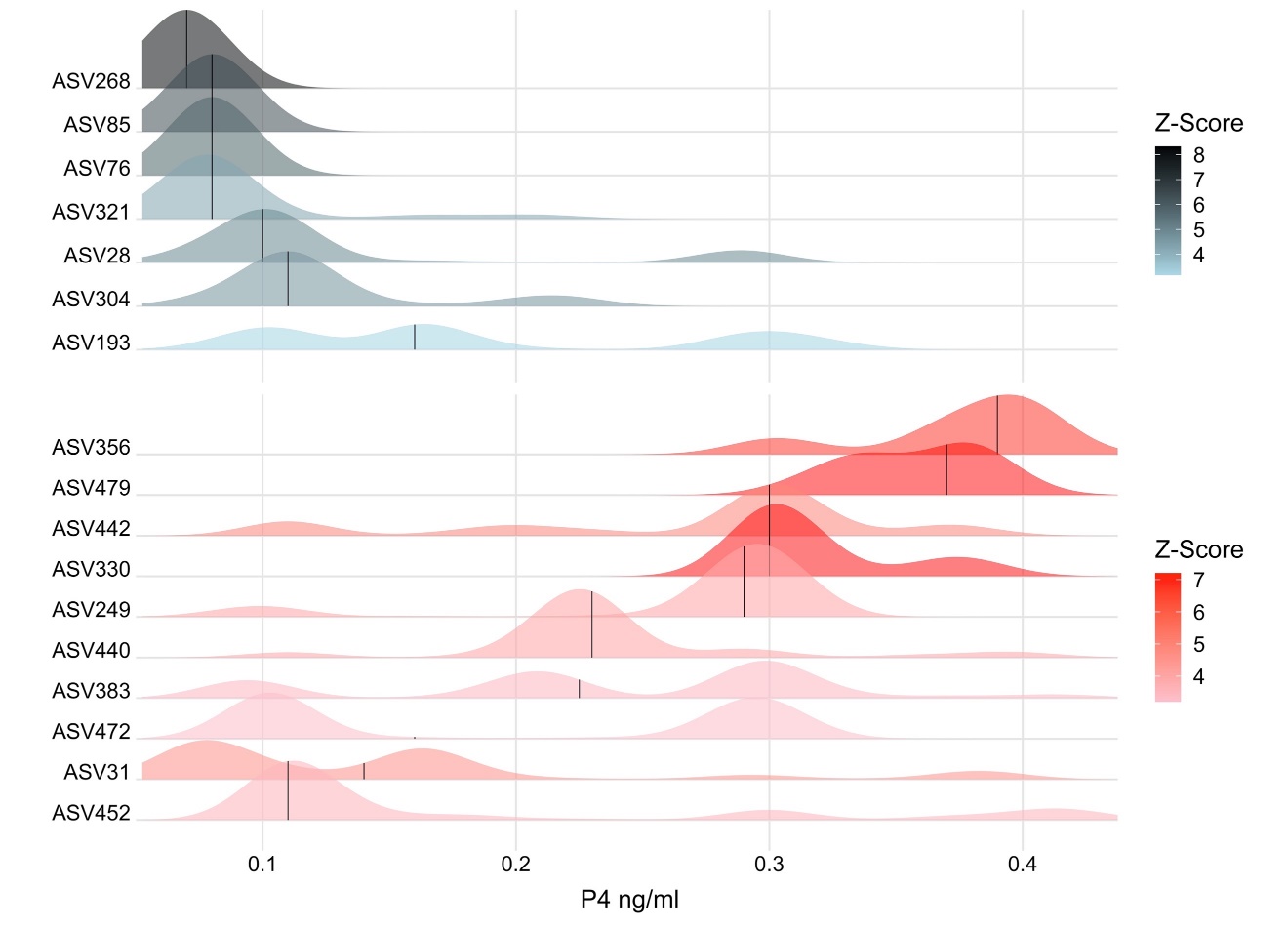


**Figure S27. Non-linear relationships between bacterial ASV relative abundance and progestagen in African elephants**


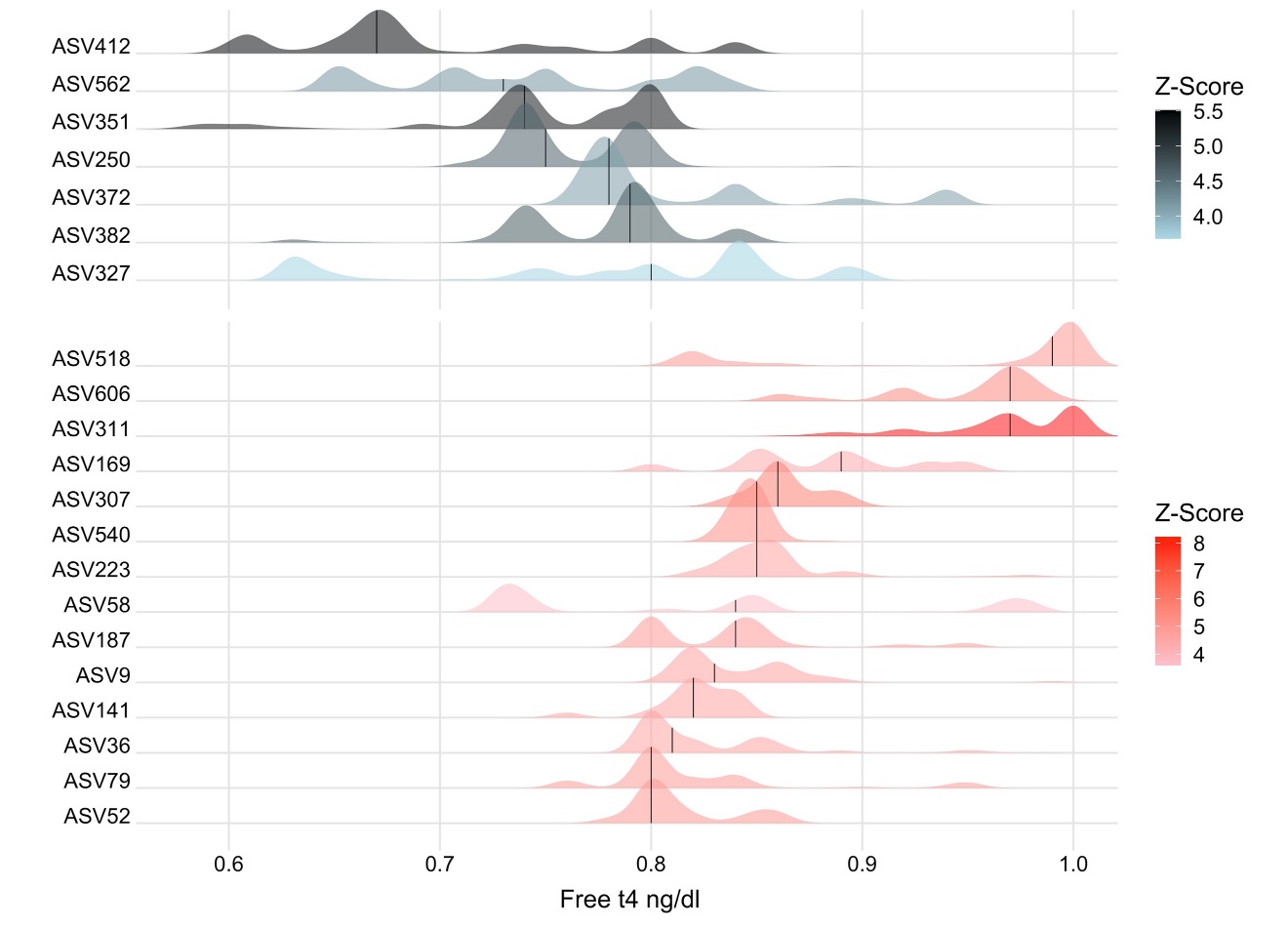


**Figure S28. Non-linear relationships between bacterial ASV relative abundance and free T4 in African elephants**


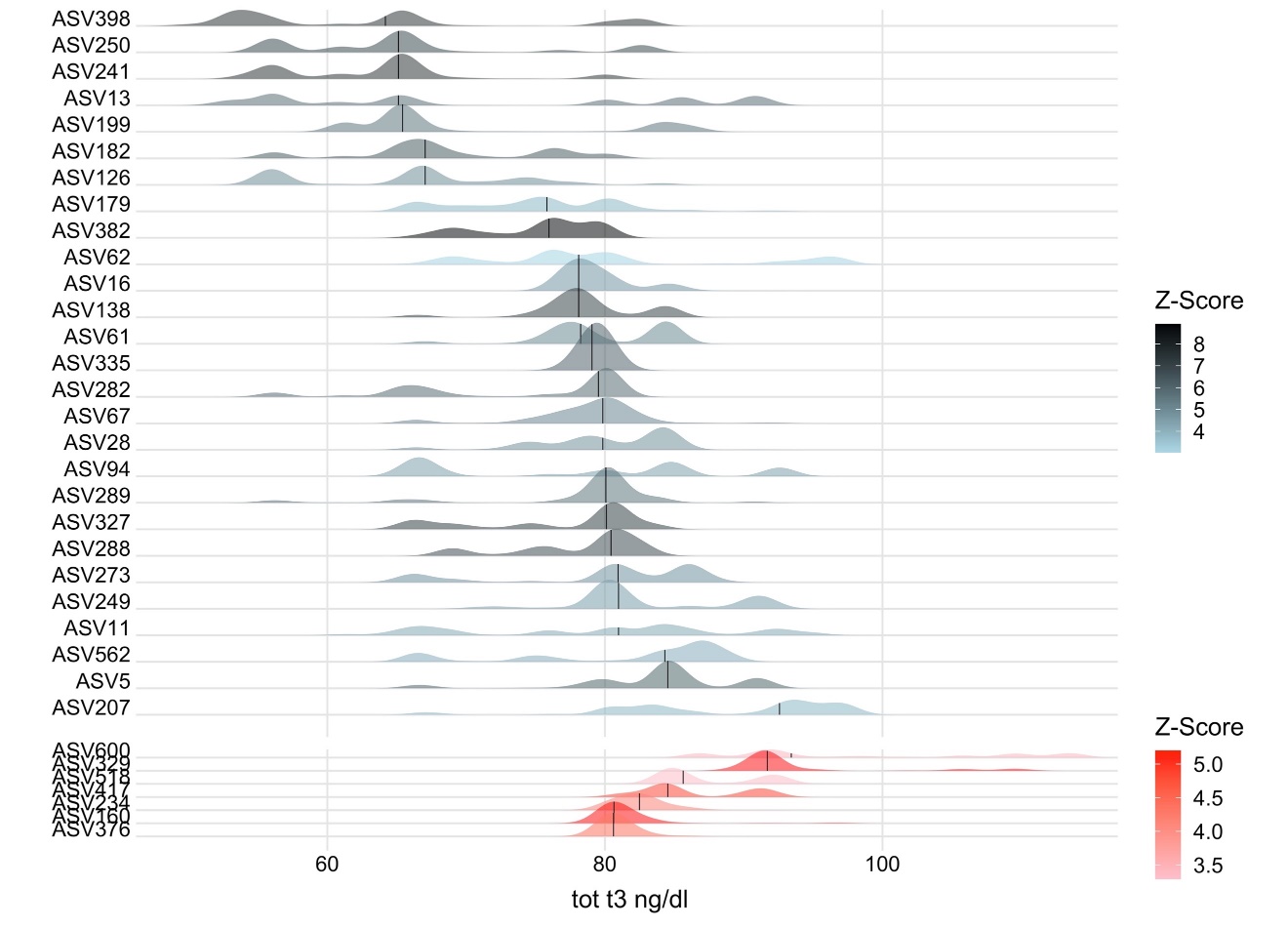


**Figure S29. Non-linear relationships between bacterial ASV relative abundance and total T3 in African elephants**


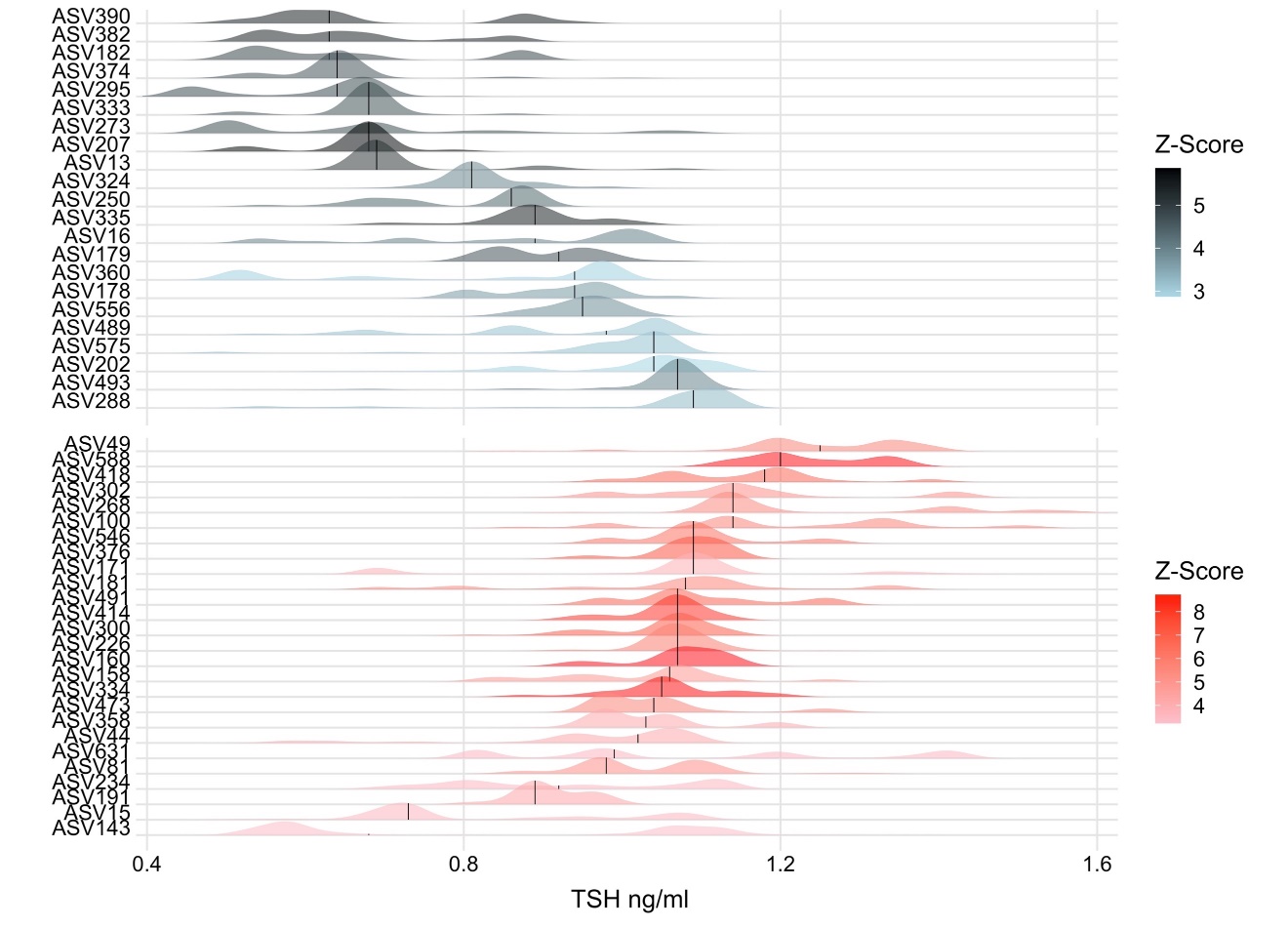


**Figure S30. Non-linear relationships between bacterial ASV relative abundance and total TSH in African elephants**


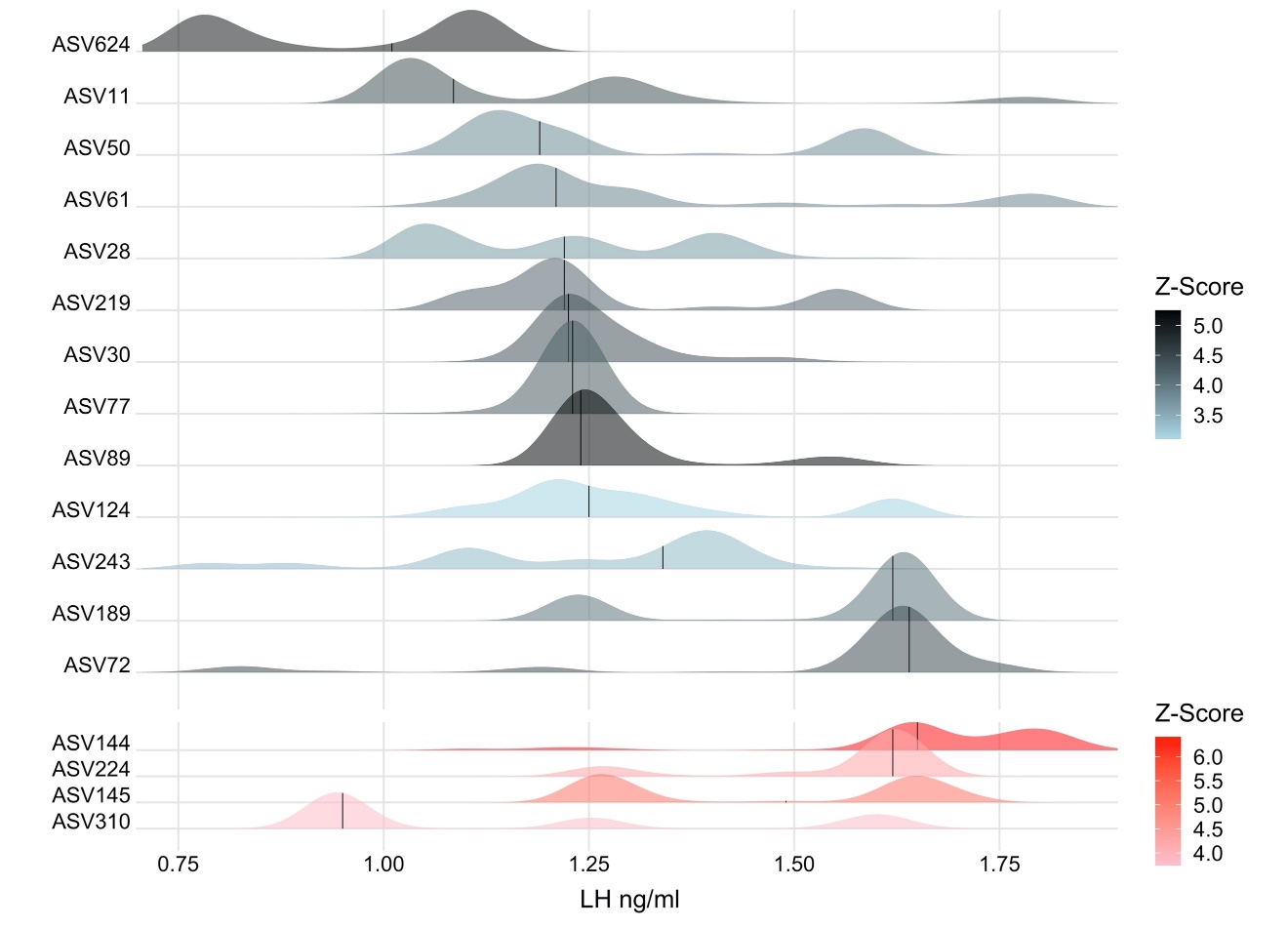


**Figure S31. Non-linear relationships between bacterial ASV relative abundance and LH in Asian elephants**


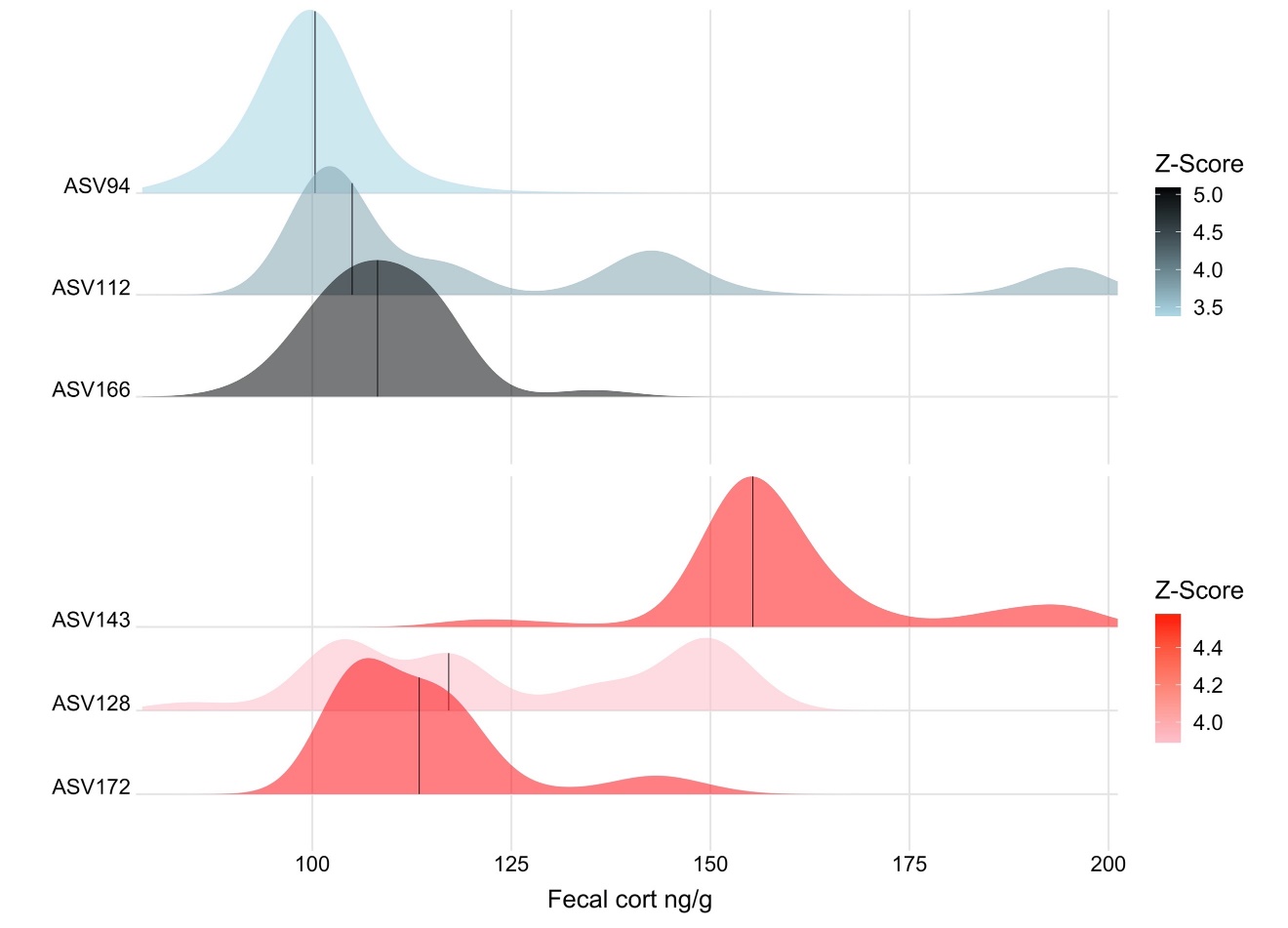


**Figure S32. Non-linear relationships between bacterial ASV relative abundance and FGM in Asian elephants**


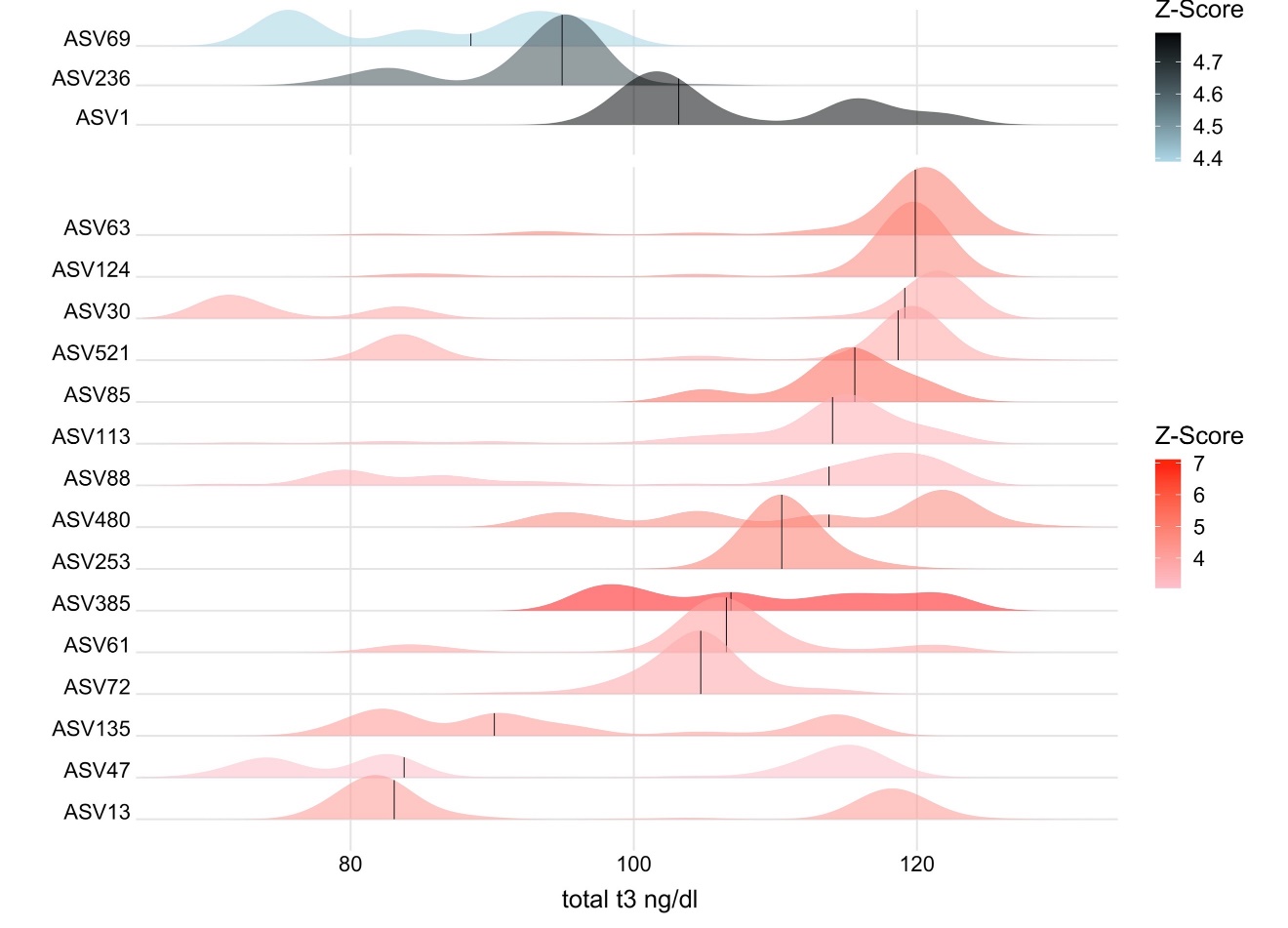


**Figure S33. Non-linear relationships between bacterial ASV relative abundance and total T3 in Asian elephants**


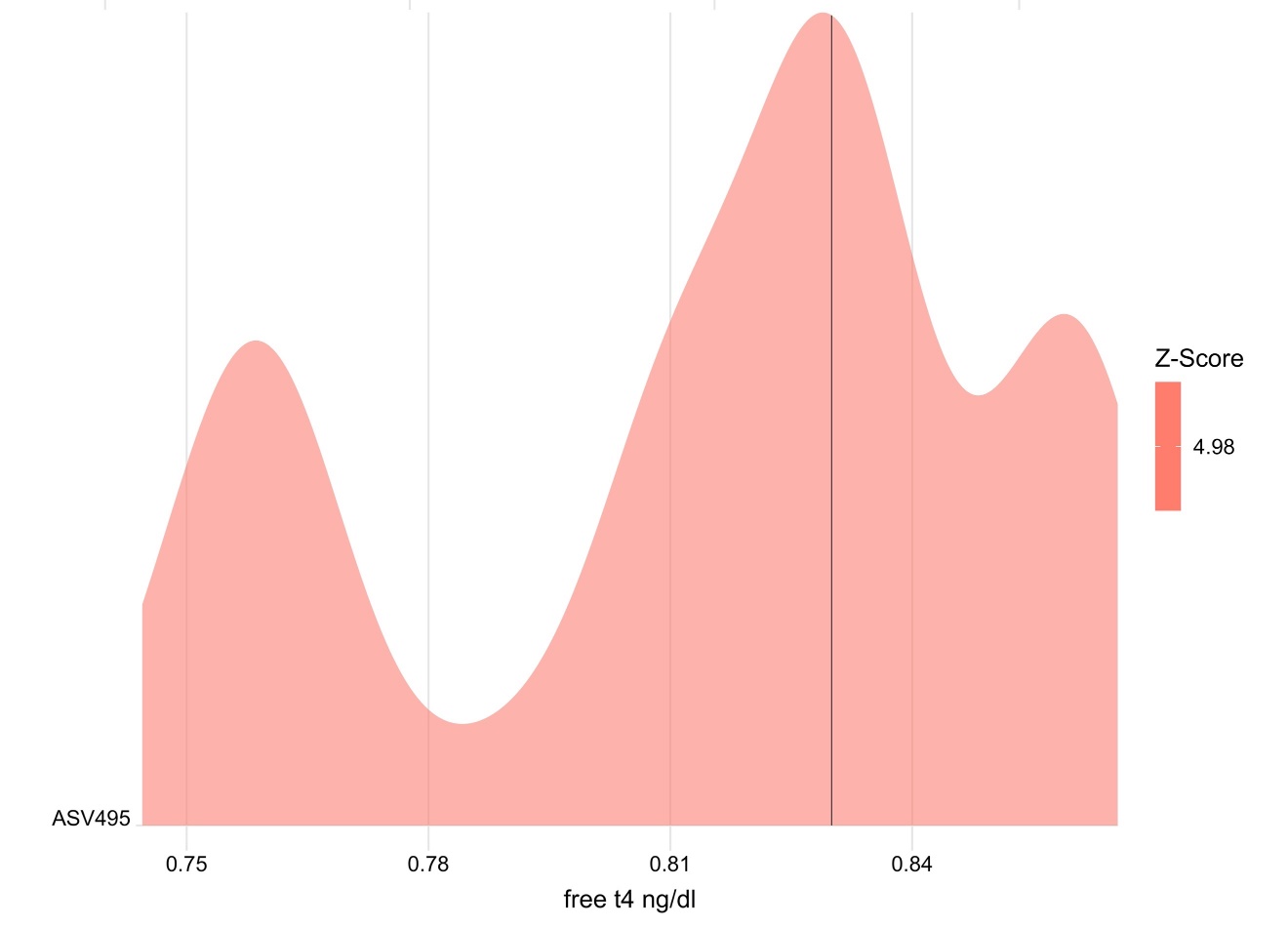


**Figure S34. Non-linear relationships between bacterial ASV relative abundance and free T4 in Asian elephants**
